# The First Spectrum of Manganese, Mn I

**DOI:** 10.6028/jres.068A.003

**Published:** 1964-02-01

**Authors:** Miguel A. Catalán, William F. Meggers, Olga Garcia-Riquelme

## Abstract

In 1894, two short series of threefold spectral terms were discovered in the arc spectrum of manganese, and in 1922 other regularities involving fivefold and sixfold terms were discovered by Catalán who coined the word “multiplet” for the group of related lines resulting from combinations of such complex terms. Multiplet analyses of complex spectra promptly led to the present formal quantum interpretation of all such phenomena, but comparable progress in the analysis of the Mn I spectrum was handicapped by the paucity of experimental data.

New observations of about 2500 wavelengths and intensities plus 440 Zeeman patterns made available in 1948–49 have now been completely exploited to derive additional atomic energy levels and thereby explain more of the observed Mn I lines. The result is that a total of 42 even terms with 125 levels and 60 *g*-values have now been designated and allocated to electron configurations, and 94 odd terms with 266 levels, 164 *g*-values, plus 13 miscellaneous levels. These terms are distributed among four multiplicities (doublets, quartets, sextets, octets), and transitions between even and odd terms account for more than 2030 lines ranging in wavelength from 1785 Å to 17608 Å.

## I. Introduction

The spectra of manganese have interested and engaged scientists for nearly 120 years. In 1910 Kayser [1]^4^ listed 67 publications on this subject since 1845, and in 1934 Kayser and Konen [2] compiled a bibliography of 154 additional items through 1931. In the present paper only a few of the above will be referred to for historical background, but most of the papers published since 1931 that describe or interpret the Mn I spectrum will be mentioned to extend the bibliography.

In 1894 Kayser and Runge [3] reported finding the first regularities among lines in the arc spectrum of manganese; they found five triplets with wave number differences of 173 and 129 K (K=kayser = 1 cm^−1^) and arranged them in two spectral series, one “sharp” and one “diffuse,” like those in the simpler spectra of alkaline-earth elements. (This precious information was presented in a footnote on page 104 of a paper primarily dedicated to the spectra of tin, lead, arsenic, antimony, and bismuth.)

Nearly three decades after Kayser and Runge [3] discovered the first rudimentary series in the arc spectrum of manganese, Catalán [4] extended those series and calculated the first ionization potential of manganese as 7.41 electron volts. Catalán said (p. 146) “Whilst the manganese series of ordinary type were under investigation, it was noted that there was a strong tendency for lines of similar character to appear in groups (of 9, 12, or 15 lines) and that such groups included some of the most intense lines in the spectrum”. For such a group of related spectral lines, “multiplet” was suggested (p. 147). “The accuracy of the separations, some of them being identical with those of the ordinary series, together with the fact that the lines of each group are of the same character, strongly suggests that the multiplets have a real physical significance. Further evidence for their reality is afforded by the occurrence of similar multiplets in the spectra of other elements” (p. 163). In fact, Catalán’s first paper on regularities in the spectrum of manganese reporting triplets and multiplets that reveal terms with five or six levels [4] also reported three multiplets in the spectrum of ionized manganese and three multiplets of the neutral atom of chromium. Even before that paper was printed, a manuscript copy of it inspired Sommerfeld [5] to write on the explanation of complex spectra (manganese, chromium, etc.) according to the method of inner quantum numbers. Almost simultaneously, Back [6] accurately measured the Zeeman effect of 49 lines in the arc- and 12 lines in the spark spectrum of manganese with the object of investigating magnetic term splitting of the complex terms found by Catalán, and Landé [7] provided an explanation of all Zeeman patterns with the aid of magnetic quantum numbers and experimental magnetic splitting factors for different types of spectral terms and multiplicities.

It was then clear that the regularities among 169 lines of Mn I presented by Catalán [4] arose from spectral terms belonging to sextet and octet systems. As a check of Landé’s scheme, and an example of the usefulness of Zeeman effect in analyzing complex spectra, Back [6] found from Zeeman data the first multiplet of the quartet system. Since multiplets usually occur in triplicate (Δ*l* = 0,±1), Catalán [8] promptly found two additional quartet multiplets associated with the one described by Back.

In 1926 McLennan and McLay [9] extended the work of Catalán and Back on Mn I by making absorption experiments, finding 14 additional multiplets, providing energy diagrams for quartets, sextets, and octets, and tables of 90 energy levels and 257 classified lines ranging in wavelength from 1874.7 Å to 17607.5 Å. Although terms of doublet multiplicity were sought, none was found at that time. This list of levels and terms, with some additions from Russell [10] and from Catalán [11], and with modernized notation, appeared in a volume of Atomic Energy States published in 1932 by Bacher and Goudsmit [12]; it presented 99 energy levels distributed in 17 quartet, 16 sextet, and 15 octet, terms then recognized for the Mn I spectrum. The following year, T. Dunham, Jr., [13] found ten new quartet and two sextet terms (but never published them), and Meggers [14] detected one quartet and two sextet terms in new observations of near infrared spectra. Aside from the above, fifteen years passed without progress in the analysis of Mn I. A strong incentive to extend this analysis came in 1946 when C. E. Moore began her critical and comprehensive compilation of Atomic Energy Levels as Derived from the Analyses of Optical Spectra.

In 1949 a major advance in the analysis of Mn I was made by Miss Olga Garcia-Riquelme [15] who was assigned the task as a thesis problem by Professor Catalán at the University of Madrid. By assembling all known energy levels of Mn I and by compiling wavelengths and intensities of manganese lines from many spectroscopic papers (by 15 different authors), Garcia-Riquelme succeeded in finding 58 new energy levels and 15 new terms for Mn I, thus producing a total of 30 even terms (85 levels), 30 odd terms (124 levels), plus 21 miscellaneous odd levels beyond the ionization limit. The permitted transitions between these levels accounted for 711 observed lines ranging in wavelength from 1876.48 A to 17607.5 Å. All the established spectral terms of Mn I were assigned to quartet, sextet, and octet systems; doublet terms were still unrecognized.

In order to complete the bibliography on description and interpretation of Mn I spectra during the period 1931 to 1949, we mention a paper by Slevogt [16] and one by Paul [17]. The former quotes earlier data on 233 lines (between 6024.67 and 8933.03 A) and remeasured the wavelengths of 113 of these; the latter reported observations on 57 Mn I lines with wavelengths between 1923.05 Å and 1085.01 Å absorbed by manganese vapor. Paul explained 10 of these as transitions from the ground state to highly excited levels, but only three of these have been confirmed in this analysis of Mn I.

In 1948 Catalán decided that an improved description of the Mn I spectrum (including reliable wavelengths, realistic relative intensities for several thousand lines, and Zeeman data for hundreds of lines) was prerequisite to a more complete and satisfactory term analysis of this spectrum.

## 2. Experiments

To improve the data of Mn I Catalán spent most of 1948 in the United States, where he recorded the vacuum ultraviolet arc spectrum of manganese at Princeton University, and photographed the arc spectrum throughout the visible and adjacent ultraviolet at the National Bureau of Standards.

The vacuum ultraviolet arc and spark spectra of manganese were photographed with a two-m radius grating at Princeton University through the courtesy of Allen G. Shenstone who previously described [18] the apparatus and procedure. The manganese spectra were recorded in this vacuum spectrograph from 1460 Å to 2180 Å with a scale of 4.8 Å per mm, and all these spectrograms were measured and converted to wavelengths by Catalán.

In 1933, Meggers photographed and measured an ultraviolet range (2100 Å to 2800 Å) of manganese spectra with the largest quartz Littrow spectrograph constructed by Adam Hilger, Ltd., of London. Provided with a quartz lens of three-m focal length and Cornu prisms of 60-cm total base, this spectrograph produced reciprocal dispersions of 0.4 to 1.0 Å/mm at the above-mentioned wavelengths. These results were presented to Catalán in 1948 in addition to several dozen new lines between 10500 Å and 12000 Å recorded by Meggers on Eastman 1–Z photographic plates to extend his earlier observations [14] made with Eastman 1–Q plates.

In 1948 Meggers and Catalán reobserved the arc and spark spectra of manganese between 2200 Å and 7800 Å with the aid of a concave grating supplied by R. W. Wood [19]. In a Wadsworth-type mounting, this grating of 30,000 lines per inch on aluminized pyrex glass with 22 feet radius of curvature provided ghost-free spectra with reciprocal dispersions of 1.7 to 2.4 Å per mm in the first order. The second order spectra with double dispersion were photographed between 2500 Å and 4500 Å. All these spectrograms were measured relative to international standards of wavelength provided by the iron arc, and improved wavelengths and estimated intensities of more than 2000 lines characteristic of Mn I were thus obtained in 1949. Excepting several hundred wavelengths since reported in papers on the Zeeman effect, none of these new data has been published heretofore. To complete this description of the Mn I spectra, we (like others) have quoted 16 infrared wavelengths (12900 Å to 17608 Å) emitted by arcs of 60 to 80 amperes and detected with a thermopile in 1919 by Randall and Barker [20].

This improved description of the Mn I spectrum promptly led to the detection of doublet terms and extension of other multiplicities, so that by 1952 as compared with 1949, the total number of even levels had increased to 118 from 87, the number of odd levels to 217 from 124, and the number of classified lines to 1500 from 711. This improvement in the term analysis of Mn I, including *g* (magnetic splitting) factors for 108 levels, was summarized in 1952 by Charlotte E. Moore [21].

Although Back [6] in 1923 clearly demonstrated the great value of Zeeman effect in spectral term analyses, a quarter of a century elapsed before further determinations of inner quantum numbers and magnetic splitting factors were undertaken for Mn I.

Through the courtesy of George R. Harrison, Catalán was invited, in 1949, to photograph the Zeeman effect in manganese spectra with the Bitter magnet and large concave gratings previously described by Harrison and Bitter [22]. The electrodes were prepared with pure manganese powder mixed with pure silver powder, then pressed and sintered to form solid rods about 3 mm square. These electrodes were ignited in a d-c arc operated in a magnet producing a field of 85,000 oersteds, and the spectra were photographed between 2300 Å and 6500 Å. These spectrograms were measured and computed partly at the National Bureau of Standards in Washington and partly at the Institute of Optics in Madrid.

In those experiments, the magnetic field intensity was determined from the splitting of the resonance lines (3280.7 and 3382.9 Å) of the silver matrix, and/or from the resonance lines (3933.7 Å and 3968.5 Å) of ionized calcium, present as an impurity, assuming that the Zeeman patterns of those lines agree exactly with the Landé predictions [7]. Then all displacements of Zeeman components and magnetic splitting factors were expressed in Lorentz units (the unit displacement characterizing all singlet levels). As a result of these experiments, Zeeman data have been obtained for 440 lines of Mn I, ranging from 2461.0 Å to 6021.8 A, thus providing material for several reports extending over a decade.

The observations of normal Zeeman splitting in Mn I by Back [6] in 1923 were made in magnetic fields of 37,000 oersteds, whereas the Zeeman-effect spectrograms obtained by Catalán in 1949 recorded magnetic splitting in fields of 85,000 oersteds. Catalán and Velasco [23] observed changes in *g*-values of Mn I with magnetic fields above 80,000 oersteds which distort some Zeeman patterns. These distortions in the case of $z^{6}F_{0½}^{{}^{\circ}}$ and $z^{6}F_{1½}^{{}^{\circ}}$ levels were ascribed to repulsions between magnetic levels with equal *M*-values, belonging to adjacent levels of a spectral term, and it was shown that the theory of partial Paschen-Back effects provides a simple rule for obtaining correct *g*-values in spite of asymmetries in Zeeman patterns. This was elaborated by Catalán [24] who discussed in detail a dozen distorted patterns and concluded that the experimental *g*-values agreed with those predicted for *LS* coupling by Landé [7]. Finally, Espinosa [25, 26], in connection with his doctoral thesis, made a complete theoretical interpretation of the very complex Paschen-Back patterns of the *z* ^6^P°—*e* ^6^D and *a* ^6^S—*z* ^6^P° multiplets of Mn I.

The major contributions to *g*-factors for Mn I levels have been published in two papers separated by nine years. The first, by Catalán and Garcia-Riquelme [27], reported measurements of Zeeman patterns for 128 lines ranging in wavelength from 2794.817 Å to 4823.528 Å, and derived *g*-factors for 93 levels, the latter ranging in value from 0 to 67752.84 K. The second, by Riquelme et al. [28], presented measurements and interpretations of the Zeeman patterns of 314 lines (2573 Å to 6022 Å), including derived *g*-values for 105 energy levels. The data for 251 lines were totally interpreted, whereas 63 lines showing some Paschen-Back effect were partially interpreted as confirming the spectral classification. In addition to the above, Garcia-Riquelme [29] has compiled and exploited inferior Zeeman data for about 100 lines of Mn I, most of which appeared as pseudo doublets, triplets, or quartets because they were incompletely observed or resolved. In most cases, the Laudé type of Zeeman pattern could be recognized and for classified lines the observed Zeeman effect confirms the classification quantitatively. Because most of the Zeeman data for Mn I have been published elsewhere [23, 24, 25, 26, 27, 28] they will not be repeated here; only the type numbers [32, 34] will be listed in our list of classified lines, and the average derived *g*-values in our table of spectral terms.

Following the new observations of wavelengths and intensities of more than 2000 spectral lines belonging to Mn I, and the measurement of Zeeman patterns for nearly 500 lines, Catalán and Garcia-Riquelme undertook a complete revision of the analysis and quantum interpretation of this spectrum. A preview of this overall revision was summarized by Moore [21] in 1952 when 368 energy levels were reported to account for more than 1500 lines. This work continued practically until Catalán’s death in 1957, and further progress was made by Garcia-Riquelme until 1962 when the total number of accepted levels rose to 404 and classified lines exceeded 2000. At this point, several hundred unclassified lines, mostly weak, hazy, and without Zeeman data, were sent to the Nat ional Bureau of Standards where Meggers applied an electronic computer to a final search for new energy levels by adding the wave numbers of lines to the wave numbers of levels and seeking constant sums within tolerated limits. Among hundreds of “new energy levels”, only two were accepted as physically real; they constitute the term *a*^2^I with level values of 37148.66 and 37164.25 K. We conclude that the available material on Mn I does not permit further progress in the analysis of this spectrum. This conclusion and the fact that our material is more homogeneous and extensive than any existing description of Mn I justify publication of this paper.

## 3. Results

Our final results for Mn I classified lines are presented in table 1, in which col. 1 contains the observed wavelength (λ) in angstroms (Å); col. 2, the intensity and character; col. 3, the wave number *(σ*) in kaysers (K); col. 4, the observed minus calculated wave number in kaysers (K); col. 5, the symbols for associated energy levels; and col. 6, the Zeeman type. Since the “Air” wavelengths were measured before 1950, they were converted to vacuum wave numbers with the aid of Kayser’s Table [30] which was recently superseded by a new table published by Coleman, Bozman, and Meggers [31]. Because the average differences do not exceed 0.03 K, we have retained the older values.

In col. 2, some intensity numbers are accompanied by literal symbols for line character; these have the following meanings, *c*=complex, *d*=double, *h*=hazy, *H*=very hazy, *l*=shaded long ward, *r*=narrow self-reversal, *R*=wide self-reversal, *s*=shaded short ward, *w=wide.* Excepting self-reversals, the remaining symbols for line character frequently suggest unresolved hyperfine structure because the manganese nucleus has a spin of 5/2 (*h/*2*π*) and a magnetic moment of 3.5 nuclear magnetons.

Table 1 contains 2030 classified lines of Mn I, including 55 accepted double classifications for lack of adequate spectroscopic resolving power. The energy levels derived from these classified levels are symbolized in col. 5, and the difference between their numerical values and the vacuum wave number associated with the measured wavelength is shown in col. 4, O—C. The average O—C for all classified lines is 0.06 K.

The electron configurations, numerical values, and *g*-factors associated with the atomic-energy-level symbols in col. 5 of table 1, will be found in table 2. In the last column of table 1 appear Zeeman-type numbers for 390 classified lines of Mn I according to the description of basic types of Zeeman patterns by Back and Landé [32, 34.] Briefly, types 4, 5, 6 are restricted to even multiplicities (doublets, quartets, sextets, octets) whose energy levels always have half-integral inner-quantum number (*J*-values). In type 4, the level with larger *J* has the smaller *g*, in type 5 the level with larger *J* also has the larger *g* and in type 6, the two combining levels have equal *J* but unequal *g* values. Type 7b is a special case of types 4, 5, 6 in which the *g* values of both combining levels are equal so that the observed pattern is a pseudo triplet with one *p* component and two *n* components. In the case of equal *J* and equal *g*, the type is represented by 6, 7b, and the displacement of the *n* components in Lorentz units expresses both *g* values.

The letter C attached to a dozen type numbers in col. 6 indicates the lines with asymmetrical patterns which Catalán [24] analysed in detail and for which he derived proper *g* values. Finally, instead of type numbers, the letters P—B appear for fifty lines; these are additional lines with Zeeman patterns that exhibit partial Paschen-Back effects partially interpreted by Garcia-Riquelme et al. [28]. The Zeeman data were most helpful in this analysis of Mn I; they have confirmed our interpretation almost completely and have permitted the designation of many terms belonging to the doublet system which was the most difficult to establish. The available Zeeman data for Mn I [6, 21, 23, 24, 27, 28, 29] prove that most of the levels arising from low-energy configurations (*d*^5^*s*^2^, *d*^5^*s*, *d*^5^*sp, d*^6^*p*) exhibit a remarkably pure LS coupling of electrons, and their *g*-factors are usually identical with the theoretical Landé values, within the error of measurement. Some of the upper levels of both the even and the odd configurations present anomalous *g*-values that may be explained by intermediate coupling or by incorrect grouping of levels in designated terms. Many more Zeeman patterns could be observed if a modern light source that promotes the emission of Mn I radiation were used.

The latest information on spectral terms derived from this analysis of Mn I is presented in table 2 where electron configurations (including series limits), spectral-term designations in standard notation for *LS* electron coupling, *J*-values, relative numerical values, intervals, and observed *g*-factors are shown in successive columns. The totals are 42 even terms with 125 levels and 60 *g* values, and 94 odd terms with 266 levels plus 13 miscellaneous and 164 *g* values. In 1952 [21] the totals were 36 even terms with 109 levels and 26 *g* values, and 66 odd terms with 214 levels plus 33 miscellaneous and 81 *g* values, not counting 12 abandoned levels. It is seen that the present analysis of the Mn I spectrum is a considerable improvement over any preceding, but, if a comparison is made of the presently recognized terms with the table of predicted terms [21, pp. XIV, XV], any claim that it is satisfactorily completed is unjustified. In order to show the progress that has rewarded this problem during the past 40 years, we briefly summarize in table 3 the number of classified Mn I lines and derived atomic energy levels at different times during this period.

The present analysis has effectively exhausted all available data on wavelengths, intensities, and Zeeman patterns of Mn I so that no further progress can be made with that material. But now we know that an arc between manganese electrodes at atmospheric pressure, with which all observations were made, is an inferior light source. Because manganese is a relatively light atom (A-55), and the arc has a high temperature (>5000 °C), it produces broad lines which are always very hazy and unsymmetrical when they involve high energy levels. All complex Mn I terms that converge to *a*^7^S_3_ or *a*^5^S_2_ limits in Mn II have very small intervals and are mostly unresolved because of the excessive line widths. Furthermore, the traditional arc in air always produces a strong background of molecular spectra that often masks atomic lines. All of these spectroscopic objections to an arc in air can now be avoided by using highly evacuated electrodeless (quartz tube) lamps containing a trace of metal or halide compound excited with ultrahigh frequency as demonstrated by Meggers and Westfall [33] for mercury and by Corliss and Meggers [34] for hafnium. At elevated temperatures (ca. 800 °C) such lamps favor spectra of neutral atoms, especially important for faint lines and for observing first spectra in strong magnetic fields; we mention this to aid anyone who becomes ambitious to make further progress in the description and quantum interpretation of the Mn I spectrum. That such progress is desirable becomes obvious when the now known spectral terms (table 2) are compared with the predicted ones [21]. Although many electron configurations and spectral terms have been identified, rarely is a configuration represented by all its terms and in some cases the terms themselves are fragmentary or even uncertain.

In 1922, Catalán derived, from spectral series of running *s* electrons, the absolute energy of the ground state of Mn I, and computed the first ionization potential to be 7.41 electron volts. Thirty years later Catalán and Velasco [35] systematically studied the first three ionization potentials of elements in the iron group; for Mn I they obtained an absolute value of 59960 K for the ground state, the equivalent 7.432 eV. These values were quoted in the second volume of Atomic Energy Levels [21]. A decade later, Garcia-Riquelme [36] applied a Ritz formula to a series of ^6^F terms with four members (4f, 5f, 6f, 7f) and found the limit near 59979 K or 7.434 eV. We decided to adopt the average of the last two determinations resulting in a limit of 59970 K or ionization potential of 7.433 eV for normal manganese.

## Figures and Tables

**Table 1:** Mn I—Classified lines

1	2	3	4	5	6
					
*λ*	Int.	*σ*	o—c	Term designation	Zeeman type
					
Å		*K*	*K*		
Vac					
1785.355	7*h*	56011.26	−1.16	*a* ^6^S_2½_—*s* ${\;}^{6}P_{3½}^{{}^{\circ}}$	
1785.465	5*h*	56007.81	−0.10	*a* ^6^S_2½_—*s* ${\;}^{6}P_{2½}^{{}^{\circ}}$	
1785.829	6*h*	55996.40	−0.22	*a* ^6^S_2½_—*s* ${\;}^{6}P_{1½}^{{}^{\circ}}$	
1788.152	2*h*	55923.65	−0.16	*a* ^6^S_2½_— $55923_{2½}^{{}^{\circ}}$	
1875.727	10 *r*	53312.66	1.29	*a* ^6^S_2½_—*t* ${\;}^{6}P_{1½}^{{}^{\circ}}$	
1876.445	15*r*	53292.26	0.68	*a* ^6^S_2½_—*t* ${\;}^{6}P_{2½}^{{}^{\circ}}$	
1877.518	20*r*	53261.80	0.38	*a* ^6^S_2½_—*t* ${\;}^{6}P_{3½}^{{}^{\circ}}$	
1882.366	3	53124.63	0.54	*a* ^6^S_2½_—*y* ${\;}^{4}D_{3½}^{{}^{\circ}}$	
1882.900	1	53109.56	0.35	*a* ^6^S_2½_—*y* ${\;}^{4}D_{2½}^{{}^{\circ}}$	
1883.085	0	53104.34	1.15	*a* ^6^S_2½_—*y* ${\;}^{4}D_{1½}^{{}^{\circ}}$	
1890.962	1	52883.13	−0.74	*a* ^6^S_2½_—*x* ${\;}^{6}D_{2½,1½}^{{}^{\circ}}$	
1891.414	3	52870.49	0.39	*a* ^6^S_2½_— ${\;}^{6}D_{3½}^{{}^{\circ}}$	
1913.752	(15)	52253.57	0.33	*a* ^6^S_2½_—*u* ${\;}^{6}P_{3½}^{{}^{\circ}}$	
1918.328	(15)	52128.73	0.08	*a* ^6^S_2½_—*u* ${\;}^{6}P_{2½}^{{}^{\circ}}$	
1922.516	(12)	52015.17	0.17	*a* ^6^S_2½_—*u* ${\;}^{6}P_{1½}^{{}^{\circ}}$	
1949.100	3	51305.73	0.32	*a* ^6^S_2½_—*x* ${\;}^{4}P_{2½}^{{}^{\circ}}$	
1996.056	50R	50098.79	−0.37	*a* ^6^S_2½_—*v* ${\;}^{6}P_{1½}^{{}^{\circ}}$	
1999.511	100R	50012.23	−0.30	*a* ^6^S_2½_—*v* ${\;}^{6}P_{2½}^{{}^{\circ}}$	
Air					
2003.849	200R	49887.73	−0.35	*a* ^6^S_2½_—*v* ${\;}^{6}P_{3½}^{{}^{\circ}}$	
2017.630	3	49547.04	−0.84	*a* ^6^D_2½_— $66600_{3½}^{{}^{\circ}}$?	
2018.332	4	49529.78	0.00	*a* ^6^D_2½_— $66981_{3½}^{{}^{\circ}}$	
2070.988	5	48270.66	−0.25	*a* ^6^S_2½_—*y* ${\;}^{6}F_{2½}^{{}^{\circ}}$	
2072.917	12	48225.74	−0.25	*a* ^6^S_2½_—*y* ${\;}^{6}F_{3½}^{{}^{\circ}}$	
2092.159	500R	47782.26	−0.17	*a* ^6^S_2½_—*u* ${\;}^{6}P_{1½}^{{}^{\circ}}$	
2092.516	50R	47774.09	−0.43	*a* ^6^S_2½_—*y* ${\;}^{6}D_{3½}^{{}^{\circ}}$	
2093.407	200R	47753.77	−0.22	*a* ^6^S_2½_—*y* ${\;}^{6}D_{2½}^{{}^{\circ}}$	
2097.554	30	47659.38	−0.14	*a* ^6^S_2½_—*w* ${\;}^{6}P_{2½}^{{}^{\circ}}$	
2106.052	100R	47467.10	0.44	*a* ^6^S_2½_—*y* ${\;}^{6}D_{1½}^{{}^{\circ}}$	
2109.585	300R	47387.60	−0.02	*a* ^6^S_2½_—*w* ${\;}^{6}P_{3½}^{{}^{\circ}}$	
2173.195	3*h*	46001.72	0.95	*a* ^6^S_2½_—*y* ${\;}^{8}P_{3½}^{{}^{\circ}}$?	
2174.12	0*h*	45981.2	−0.2	*a* ^6^S_2½_—*y* ${\;}^{8}P_{2½}^{{}^{\circ}}$	
2176.014	2	45941.14	0.21	*a* ^6^S_2½_—*z* ${\;}^{4}D_{2½}^{{}^{\circ}}$	
2182.773	15	45798.90	−0.28	*a* ^6^D_4½_—*w* ${\;}^{6}D_{3½}^{{}^{\circ}}$	
2184.912	10	45754.07	−0.20	*a* ^6^S_2½_—*z* ${\;}^{4}D_{3½}^{{}^{\circ}}$	
2190.884	2	45629.37	−0.39	*a* ^6^D_2½_—*y* ${\;}^{2}D_{2½}^{{}^{\circ}}$	
2191.413	100	45618.36	−0.16	*a* ^6^D_4½_—*w* ${\;}^{6}D_{4½}^{{}^{\circ}}$	
2193.762	20	45569.51	0.04	*a* ^6^D_3½_—*w* ${\;}^{6}D_{3½}^{{}^{\circ}}$	
2196.503	3	45512.65	−0.15	*a* ^6^D_1½_—*y* ${\;}^{2}D_{2½}^{{}^{\circ}}$	
2198.131	10	45478.94	−0.39	*a* ^6^D_3½_—*w* ${\;}^{6}D_{2½}^{{}^{\circ}}$	
2199.41	1*h*	45452.5	−0.5	*a* ^6^D_4½_—*v* ${\;}^{4}F_{3½}^{{}^{\circ}}$	
2201.960	7	45399.88	−0.07	*a* ^6^D_2½_—*w* ${\;}^{6}D_{3½}^{{}^{\circ}}$	
2202.489	2	45388.97	0.16	*a* ^6^D_3½_—*w* ${\;}^{6}D_{4½}^{{}^{\circ}}$	
2205.057	10	45336.11	0.00	*a* ^6^D_2½_—*w* ${\;}^{6}D_{1½}^{{}^{\circ}}$	
2206.343	2	45309.70	−0.11	*a* ^6^D_2½_—*w* ${\;}^{6}D_{2½}^{{}^{\circ}}$	
2208.806	200R	45259.17	0.00	*a* ^6^S_2½_—*x* ${\;}^{6}P_{1½}^{{}^{\circ}}$	
2210.582	8*h*	45222.81	−0.48	*a* ^6^D_3½_—*v* ${\;}^{4}F_{3½}^{{}^{\circ}}$	
2211.720	8	45199.55	−0.13	*a* ^6^D_1½_—*w* ${\;}^{6}D_{0½}^{{}^{\circ}}$	
2212.055	15	45192.70	−0.15	*a* ^6^D_1½_—*w* ${\;}^{6}D_{2½}^{{}^{\circ}}$	
2213.855	300R	45155.96	−0.15	*a* ^6^S_2½_—*x* ${\;}^{6}P_{2½}^{{}^{\circ}}$	
2214.10	10	45150.4	−0.1	*a* ^6^D_0½_—*w* ${\;}^{6}D_{1½}^{{}^{\circ}}$	
2215.086	3	45130.89	−0.12	*a* ^6^D_0½_—*w* ${\;}^{6}D_{0½}^{{}^{\circ}}$	
2218.903	3*h*	45053.24	−0.53	*a* ^6^D_2½_—*v* ${\;}^{4}F_{3½}^{{}^{\circ}}$	
2221.837	500R	44993.76	−0.16	*a* ^6^S_2½_—*x* ${\;}^{6}P_{3½}^{{}^{\circ}}$	
2249.911	5*s*	44432.39	−0.13	*a* ^6^D_3½_—*y* ${\;}^{2}G_{4½}^{{}^{\circ}}$	
2258.714	2	44259.24	−0.22	*a* ^6^D_2½_—*z* ${\;}^{2}F_{3½}^{{}^{\circ}}$	
2262.294	8*s*	44189.21	−0.02	*a* ^6^D_3½_—*w* ${\;}^{4}G_{2½}^{{}^{\circ}}$	
2277.065	0*h*	43902.60	−0.15	*a* ^6^D_1½_—*w* ${\;}^{4}G_{2½}^{{}^{\circ}}$	
2288.449	20	43684.22	−0.41	*a* ^4^D_3½_— $66981_{3½}^{{}^{\circ}}$	
2292.189	30	43612.95	−0.40	*a* ^4^D_3½_— $66910_{2½}^{{}^{\circ}}$	
2293.122	2	43595.21	−0.29	*a* ^6^S_2½_—*z* ${\;}^{6}F_{2½}^{{}^{\circ}}$	
2296.880	5	43523.88	−0.20	*a* ^6^S_2½_—*z* ${\;}^{6}F_{3½}^{{}^{\circ}}$	
2298.876	20	43486.10	−0.28	*a* ^4^D_3½_—*u* ${\;}^{4}F_{3½}^{{}^{\circ}}$	
2300.300	8	43459.18	−0.16	*a* ^4^D_2½_— $67008_{2½}^{{}^{\circ}}$	
2300.728	3H	43451.10	−0.18	*a* ^6^D_2½_—*w* ${\;}^{4}F_{3½}^{{}^{\circ}}$	
2301.260	1*d*	43441.07	−0.08	*a* ^4^D_3½_—*v* ${\;}^{2}G_{3½}^{{}^{\circ}}$	
2301.748	4	43431.84	−0.26	*a* ^4^D_2½_— $66981_{3½}^{{}^{\circ}}$	
2305.518	5	43360.83	0.01	*a* ^4^D_2½_— $66910_{2½}^{{}^{\circ}}$	
2305.703	15H	43357.35	−0.63	*a* ^4^D_3½_— $66654_{2½}^{{}^{\circ}}$	
2309.057	2*h*	43294.38	−0.21	*a* ^4^D_2½_—*u* ${\;}^{4}F_{1½}^{{}^{\circ}}$	
2309.374	10	43288.45	0.01	*a* ^4^D_2½_—*u* ${\;}^{4}F_{2½}^{{}^{\circ}}$	
2312.304	20	43233.59	−0.26	*a* ^4^D_1½_—*u* ${\;}^{4}F_{3½}^{{}^{\circ}}$	
2318.17	1*h*	43124.2	−0.1	*a* ^4^D_1½_—*u* ${\;}^{4}F_{1½}^{{}^{\circ}}$	
2318.501	2*h*	43118.04	−0.08	*a* ^4^D_1½_—*u* ${\;}^{4}F_{2½}^{{}^{\circ}}$	
2321.995	10*h*	43053.16	0.00	*a* ^4^G_4½_—*u* ${\;}^{2}G_{3½}^{{}^{\circ}}$	
2322.106	3*h*	43051.11	$\left\{ \;\begin{array}{l} 0.26\\ 0.14 \end{array}\right.$	*a* ^4^G_3½_—*u* ${\;}^{2}G_{3½}^{{}^{\circ}}$ *a* 4D_2½_— $66600_{3½}^{{}^{\circ}}$	
2323.748	30*h*	43020.70	0.00	*a* ^4^G_5½_—*u* ${\;}^{2}G_{4½}^{{}^{\circ}}$	
2324.803	5*h*	43001.17	0.16	*a* ^4^G_4½_—*u* ${\;}^{2}G_{4½}^{{}^{\circ}}$	
2327.308	2H	42954.89	−0.12	*a* ^4^D_2½_— $66504_{1½}^{{}^{\circ}}$	
2342.088	3H	42683.85	0.02	*a* ^4^G_2½_—*t* ${\;}^{4}G_{2½}^{{}^{\circ}}$	
2342.771	1H	42676.87	−0.26	*a* ^4^G_3½_—*t* ${\;}^{4}G_{2½}^{{}^{\circ}}$	
2346.126	1H	42610.38	0.06	*a* ^4^G_2½_—*t* ${\;}^{4}G_{3½}^{{}^{\circ}}$	
2346.383	2*h*	42605.72	−0.21	*a* ^4^G_4½_—*t* ${\;}^{4}G_{3½}^{{}^{\circ}}$	
2346.497	5*h*	42603.65	0.03	*a* ^4^G_3½_—*t* ${\;}^{6}G_{3½}^{{}^{\circ}}$	
2349.263	3*h*	42553.50	0.07	*a* ^4^G_5½_—*t* ${\;}^{4}G_{4½}^{{}^{\circ}}$	
2350.352	10*h*	42533.78	0.04	*a* ^4^G_4½_—*t* ${\;}^{4}G_{4½}^{{}^{\circ}}$	
2352.937	20*h*	42487.05	−0.05	*a* ^4^G_5½_—*t* ${\;}^{4}G_{5½}^{{}^{\circ}}$	
2354.020	4*h*	42467.51	0.10	*a* ^4^G_4½_—*t* ${\;}^{4}G_{5½}^{{}^{\circ}}$	
2357.899	2*h*	42397.65	−0.02	*a* ^4^D_2½_—*w* ${\;}^{2}D_{2½}^{{}^{\circ}}$	
2362.719	1	42311.17	0.07	*a* ^4^G_5½_—*w* ${\;}^{2}H_{4½}^{{}^{\circ}}$	
2363.823	4	42291.41	0.00	*a* ^4^G_4½_—*w* ${\;}^{2}H_{4½}^{{}^{\circ}}$	
2363.956	1	42289.03	−0.07	*a* ^4^G_3½_—*w* ${\;}^{2}H_{4½}^{{}^{\circ}}$	
2366.575	1	42242.24	−0.14	*a* ^4^D_1½_—*w* ${\;}^{2}D_{1½}^{{}^{\circ}}$	
2366.744	5	42239.22	0.06	*a* ^4^G_5½_—*w* ${\;}^{2}H_{5½}^{{}^{\circ}}$	
2367.851	1	42219.47	0.00	*a* ^4^G_4½_—*w* ${\;}^{2}H_{5½}^{{}^{\circ}}$	
2372.116	10*d*	42143.56	−0.01	*a* ^6^S_2½_—*z* ${\;}^{6}D_{1½}^{{}^{\circ}}$	
2377.183	30R	42053.75	0.02	*a* ^6^S_2½_—*z* ${\;}^{6}D_{2½}^{{}^{\circ}}$	
2382.175	2	41965.63	0.02	*a* ^4^D_3½_—*w* ${\;}^{2}G_{4½}^{{}^{\circ}}$	
2384.049	40R	41932.65	0.01	*a* ^6^S_2½_—*z* ${\;}^{6}D_{3½}^{{}^{\circ}}$	
2397.732	2	41693.37	−0.19	*a* ^4^ G_3½_— $66981_{3½}^{{}^{\circ}}$	
2401.830	2	41622.24	−0.04	*a* ^4^G_3½_— $66910_{2½}^{{}^{\circ}}$	
2403.748	2	41589.03	−0.23	*a* ^4^G_5½_—*u* ${\;}^{4}F_{4½}^{{}^{\circ}}$	
2404.882	5	41569.42	−0.15	*a* ^4^G_4½_—*u* ${\;}^{4}F_{4½}^{{}^{\circ}}$	
2405.274	3	41562.65	−0.10	*a* ^4^G_2½_—*u* ${\;}^{4}F_{1½}^{{}^{\circ}}$	
2405.628	2*h*	41556.53	−0.07	*a* ^4^G_2½_—*u* ${\;}^{4}F_{2½}^{{}^{\circ}}$	
2406.017	2	41549.82	−0.08	*a* ^4^G_3½_—*u* ${\;}^{4}F_{2½}^{{}^{\circ}}$	
2409.198	2	41494.96	−0.35	*a* ^4^G_3½_—*u* ${\;}^{4}F_{3½}^{{}^{\circ}}$	
2411.415	2	41456.81	0.03	*a* ^4^G_2½_—*v* ${\;}^{2}G_{3½}^{{}^{\circ}}$	
2417.502	2	41352.44	−0.09	*a* ^4^D_3½_—*x* ${\;}^{2}G_{3½}^{{}^{\circ}}$	
2417.909	2	41345.48	−0.01	*a* ^4^G_4½_—*v* ${\;}^{2}G_{4½}^{{}^{\circ}}$	
2418.042	4	41343.20	0.02	*a* ^4^G_3½_—*v* ${\;}^{2}G_{4½}^{{}^{\circ}}$	
2420.110	30	41307.88	0.02	*a* ^4^*G*_5½_—*u* ${\;}^{4}G_{5½}^{{}^{\circ}}$	
2420.403	30	41302.88	0.04	*a* ^4^G_5½_—*u* ${\;}^{4}H_{6½}^{{}^{\circ}}$	
2421.254	20*d*	41288.38	$\{\begin{array}{c} 0.21\\ -0.39 \end{array}$	*a* ^4^G_4½_—*u* ${\;}^{4}G_{5½}^{{}^{\circ}}$ *a* 4D_3½_—*x* ${\;}^{2}G_{4½}^{{}^{\circ}}$	
2423.099	6	41256.93	0.05	*a* ^4^G_5½_—*u* ${\;}^{4}G_{4½}^{{}^{\circ}}$	
2424.260	30	41237.17	−0.02	*a* ^4^G_4½_—*u* ${\;}^{4}G_{4½}^{{}^{\circ}}$	
2424.385	1	41235.04	0.16	*a* ^4^G_3½_—*u* ${\;}^{4}G_{4½}^{{}^{\circ}}$	
2428.286	8	41168.81	−0.03	*a* ^4^G_4½_—*u* ${\;}^{4}G_{3½}^{{}^{\circ}}$	
2428.423	25	41166.49	−0.04	*a* ^4^G_3½_—*u* ${\;}^{4}G_{3½}^{{}^{\circ}}$	
2428.586	2*h*	41163.72	−0.02	*a* ^4^D_2½_—*u* ${\;}^{4}D_{2½}^{{}^{\circ}}$	
2429.233	30	41152.76	−0.05	*a* ^4^G_5½_—*u* ${\;}^{4}H_{5½}^{{}^{\circ}}$	
2430.395	35	41133.08	−0.04	*a* ^4^G_4½_—*u* ${\;}^{4}H_{5½}^{{}^{\circ}}$	
2431.520	40	41114.06	−0.09	*a* ^4^G_2½_—*u* ${\;}^{4}G_{2½}^{{}^{\circ}}$	
2431.587	4*h*	41112.92	−0.10	*a* ^4^D_3½_—*u* ${\;}^{4}D_{3½}^{{}^{\circ}}$	
2431.915	10	41107.38	−0.07	*a* ^4^G_3½_—*u* ${\;}^{4}G_{2½}^{{}^{\circ}}$	
2432.360	8	41099.86	−0.14	*a* ^4^D_2½_—*x* ${\;}^{2}G_{3½}^{{}^{\circ}}$	
2432.898	7	41090.77	0.11	*a* ^4^G_5½_—*u* ${\;}^{4}H_{4½}^{{}^{\circ}}$	
2434.071	30	41070.97	0.00	*a* ^4^G_4½_—*u* ${\;}^{4}H_{4½}^{{}^{\circ}}$	
2434.208	35	41068.65	−0.01	*a* ^4^G_3½_—*u* ${\;}^{4}H_{4½}^{{}^{\circ}}$	
2435.137	40	41053.00	−0.43	*a* ^4^G_2½_—*u* ${\;}^{4}H_{3½}^{{}^{\circ}}$	
2435.376	5	41048.96	−0.08	*a* ^4^G_4½_—*u* ${\;}^{4}H_{3½}^{{}^{\circ}}$	
2435.511	20	41046.69	−0.04	*a* ^4^G_3½_—*u* ${\;}^{4}H_{3½}^{{}^{\circ}}$	
2440.415	2	40964.19	−0.24	*a* ^4^D_1½_—*u* ${\;}^{4}D_{1½}^{{}^{\circ}}$	
2446.159	1	40868.03	−0.03	*a* ^4^G_2½_—*u* ${\;}^{2}F_{3½}^{{}^{\circ}}$	
2446.561	2	40861.31	−0.05	*a* ^4^G_3½_—*u* ${\;}^{2}F_{3½}^{{}^{\circ}}$	
2446.610	2	40860.49	0.00	*a* ^4^D_2½_—*u* ${\;}^{4}D_{3½}^{{}^{\circ}}$	
2449.047	2	40819.83	0.02	*a* ^4^D_0½_—*u* ${\;}^{4}D_{0½}^{{}^{\circ}}$	
2453.870	6	40739.62	0.03	*a* ^4^G_2½_—*u* ${\;}^{2}F_{2½}^{{}^{\circ}}$	
2454.262	3	40733.11	0.22	*a* ^4^G_3½_—*u* ${\;}^{2}F_{2½}^{{}^{\circ}}$	
2456.878	*3h*	40689.74	−0.08	*a* ^4^P_2½_—*t* ${\;}^{4}G_{3½}^{{}^{\circ}}$	
2458.312	2	40666.01	0.18	*a* ^4^G_2½_—*w* ${\;}^{2}D_{2½}^{{}^{\circ}}$	
2459.694	50	40643.16	−0.02	*a* ^4^G_5½_—*v* ${\;}^{4}G_{4½}^{{}^{\circ}}$	
2460.887	40	40623.46	−0.03	*a* ^4^G_4½_—*v* ${\;}^{4}G_{4½}^{{}^{\circ}}$	
2461.011	50	40621.41	−0.16	*a* ^4^G_5½_—*v* ${\;}^{4}G_{5½}^{{}^{\circ}}$	6, *7b*
2462.190	7	40601.96	0.08	*a* ^4^G_4½_—*v* ${\;}^{4}G_{5½}^{{}^{\circ}}$	
2462.596	3	40595.27	−0.03	*a* ^4^G_2½_—*v* ${\;}^{4}G_{3½}^{{}^{\circ}}$	
2462.776	20	40592.31	−0.05	*a* ^4^G_2½_—*v* ${\;}^{4}G_{2½}^{{}^{\circ}}$	
2462.863	5	40590.88	−0.03	*a* ^4^G_4½_—v ${\;}^{4}G_{3½}^{{}^{\circ}}$	
2463.005	40	40588.53	−0.07	*a* ^4^G_3½_—*v* ${\;}^{4}G_{3½}^{{}^{\circ}}$	
2463.182	6	40585.61	−0.05	*a* ^4^G_2½_—*v* ${\;}^{4}G_{2½}^{{}^{\circ}}$	
2468.207	2	40502.99	−0.08	*a* ^4^G_5½_— $65768_{4½}^{{}^{\circ}}$	
2469.407	40	40483.31	−0.07	*a* ^4^G_4½_— $65768_{4½}^{{}^{\circ}}$	
2470.330	4	40468.19	−0.04	*a* ^4^D_3½_—*x* ${\;}^{4}D_{2½}^{{}^{\circ}}$	
2478.803	*2h*	40329.87	0.24	*a* ^4^G_3½_—*v* ${\;}^{4}F_{3½}^{{}^{\circ}}$	
2481.431	1	40287.16	−0.01	*a* ^4^D_3½_— $63583_{2½}^{{}^{\circ}}$	
2483.743	8	40249.66	0.03	*a* ^4^D_3½_— $63546_{3½}^{{}^{\circ}}$	
2485.114	10*hw*	40227.46	0.31	*a* ^4^D_3½_— $63523_{2½}^{{}^{\circ}}$	
2485.842	5h	40215.68	−0.02	*a* ^4^D_2½_—*x* ${\;}^{2}D_{2½}^{{}^{\circ}}$	
2491.414	2	40125.75	−0.05	*a* ^4^D_1½_—*x* ${\;}^{2}D_{1½}^{{}^{\circ}}$	
2494.391	20	40077.86	0.00	*a* ^4^D_3½_— $63374_{4½}^{{}^{\circ}}$	
2494.585	5	40074.74	−0.15	*a* ^4^D_3½_— $63371_{2½}^{{}^{\circ}}$	
2496.048	7	40051.25	0.01	*a* ^4^D_3½_—*y* ${\;}^{2}H_{4½}^{{}^{\circ}}$	
2496.415	3	40045.37	−0.01	*a* ^4^D_1½_—*x* ${\;}^{2}D_{2½}^{{}^{\circ}}$	
2497.084	4	40034.64	0.00	*a* ^4^D_2½_— $63583_{2½,\;1½}^{{}^{\circ}}$	
2497.597	4	40026.42	−0.03	*a* ^4^D_0½_—*x* ${\;}^{2}D_{1½}^{{}^{\circ}}$	
2497.725	15	40024.37	0.28	*a* ^4^G_2½_—*w* ${\;}^{2}G_{3½}^{{}^{\circ}}$	
2499.429	8	39997.08	−0.02	*a* ^4^D_2½_— $63546_{3½}^{{}^{\circ}}$	
2500.692	4	39976.88	0.03	*a* ^4^D_4½_—*w* ${\;}^{2}G_{4½}^{{}^{\circ}}$	
2500.840	2	39974.52	$\{\begin{array}{c} -0.02\\ -0.10 \end{array}$	*a* ^4^G_3½_—*w* ${\;}^{2}G_{4½}^{{}^{\circ}}$ *a* ^4^D_2½_— $63523_{2½}^{{}^{\circ}}$	
2507.756	2*h*	39864.28	−0.04	*a* ^4^D_1½_— $63583_{2½,\;1½}^{{}^{\circ}}$	
2511.351	2	39807.21	0.21	*a* ^4^P_2½_— $67008_{2½}^{{}^{\circ}}$	
2511.538	4*h*	39804.26	−0.04	*a* ^4^D_1½_— $63523_{2½}^{{}^{\circ}}$	
2513.086	1	39779.74	−0.02	*a* ^4^P_2½_— $66981_{3½}^{{}^{\circ}}$	
2514.314	40	39760.31	−0.23	*a* ^4^P_1½_— $67008_{2½}^{{}^{\circ}}$	
2517.580	2	39708.74	0.26	*a* ^4^P_2½_— $66910_{2½}^{{}^{\circ}}$	
2517.677	1	39707.21	0.03	*a* ^4^G_2½_—*w* ${\;}^{2}F_{3½}^{{}^{\circ}}$	
2517.960	1	39702.75	−0.04	*a* ^4^G_4½_—*w* ${\;}^{2}F_{3½}^{{}^{\circ}}$	
2521.986	7	39639.37	0.08	*a* ^4^G_2½_—*v* ${\;}^{4}H_{3½}^{{}^{\circ}}$	
2522.185	2	39636.25	0.15	*a* ^4^P_2½_—*u* ${\;}^{4}F_{2½}^{{}^{\circ}}$	
2522.274	4	39634.84	−0.06	*a* ^4^G_4½_—*v* ${\;}^{4}H_{3½}^{{}^{\circ}}$	
2524.472	10	39600.34	0.08	*a* ^4^G_3½_—*v* ${\;}^{4}H_{4½}^{{}^{\circ}}$	
2525.669	8	39581.57	0.06	*a* ^4^P_2½_—*u* ${\;}^{4}F_{3½}^{{}^{\circ}}$	
2528.184	2	39542.20	0.03	*a* ^4^G_2½_—*w* ${\;}^{2}F_{2½}^{{}^{\circ}}$	
2528.700	8	39534.13	0.03	*a* ^4^G_4½_—*v* ${\;}^{4}H_{5½}^{{}^{\circ}}$	
2533.050	20	39466.24	0.10	*a* ^4^G_5½_—*v* ${\;}^{4}H_{6½}^{{}^{\circ}}$	
2533.896	2*h*	39453.07	−0.04	*a* ^4^P_2½_— $66654_{2½}^{{}^{\circ}}$	
2536.878	5H	39406.69	0.04	*a* ^4^P_1½_— $66654_{2½}^{{}^{\circ}}$	
2539.642	9	39363.81	0.04	*a* ^4^G_4½_—*x* ${\;}^{2}G_{3½}^{{}^{\circ}}$	
2539.792	5	39361.48	0.02	*a* ^4^G_3½_—*x* ${\;}^{2}G_{3½}^{{}^{\circ}}$	
2542.491	10	39319.70	0.00	*a* ^4^G_5½_—*x* ${\;}^{2}G_{4½}^{{}^{\circ}}$	
2543.593	2*h*	39302.67	0.00	*a* ^4^P_2½_— $66504_{1½}^{{}^{\circ}}$	
2543.763	4	39300.04	0.03	*a* ^4^G_4½_—*x* ${\;}^{2}G_{4½}^{{}^{\circ}}$	
2546.582	4*h*	39256.54	0.33	*a* ^4^P_1½_— $66504_{1½}^{{}^{\circ}}$	
2548.80	3H	39222.4	0.0	*a* ^4^P_0½_— $66504_{1½}^{{}^{\circ}}$	
2557.040	3	39096.00	−0.15	*a* ^4^D_3½_—*v* ${\;}^{4}F_{4½}^{{}^{\circ}}$	
2565.952	4*h*	38960.22	0.09	*a* ^6^D_4½_—*s* ${\;}^{6}P_{3½}^{{}^{\circ}}$	
2566.230	1*h*	38956.00	−0.09	*a* ^4^D_2½_—*v* ${\;}^{4}F_{3½}^{{}^{\circ}}$	
2566.783	2	38947.61	0.05	*a* ^4^P_2½_—*v* ${\;}^{2}F_{3½}^{{}^{\circ}}$	
2567.40	1H	38938.3	0.1	*a* ^4^D_2½_—*v* ${\;}^{4}F_{2½}^{{}^{\circ}}$	
2572.755	50 R	38857.20	−0.06	*z* ${\;}^{8}P_{3½}^{{}^{\circ}}$—*e* ^8^P_4½_	
2575.509	20R	38815.66	−0.03	*z* ${\;}^{8}P_{2½}^{{}^{\circ}}$—*e* ^8^P_3½_	
2577.470	6	38786.13	−0.04	*a* ^4^G_5½_—*x* ${\;}^{2}H_{5½}^{{}^{\circ}}$	
2578.358	2	38772.77	0.14	*a* ^4^P_1½_—*u* ${\;}^{2}F_{2½}^{{}^{\circ}}$	
2578.548	2	38769.92	−0.02	*a* ^4^G_4½_—*x* ${\;}^{2}H_{4½}^{{}^{\circ}}$	
2578.695	*2h*	38767.71	−0.13	*a* ^4^D_1½_—*v* ${\;}^{4}F_{2½}^{{}^{\circ}}$	
2579.18	1	38760.4	0.0	*a* ^4^D_2½_—*w* ${\;}^{2}D_{1½}^{{}^{\circ}}$	
2580.180	3	38745.40	0.07	*a* ^4^P_2½_—*w* ${\;}^{2}D_{2½}^{{}^{\circ}}$	
2581.183	2*h*	38730.34	−0.08	*a* ^6^D_3½_—*s* ${\;}^{6}P_{3½}^{{}^{\circ}}$	
2581.478	*Sh*	38725.91	0.00	*a* ^6^D_3½_—*s* ${\;}^{6}P_{2½}^{{}^{\circ}}$	
2582.270	5	38714.04	0.14	*a* ^4^P_1½_—*w* ${\;}^{2}D_{1½}^{{}^{\circ}}$	
2583.275	7	38698.98	0.11	*a* ^4^P_1½_—*w* ${\;}^{2}D_{2½}^{{}^{\circ}}$	
2584.100	10	38686.62	0.11	*^z^* ${\;}^{8}P_{3½}^{{}^{\circ}}$—*e* ^8^P_3½_	
2584.302	100R	38683.60	$\{\begin{array}{c} -0.27\\ 0.07 \end{array}$	*z* ${\;}^{8}P_{2½}^{{}^{\circ}}$—*e* ^8^P_2½_ *z* ${\;}^{8}P_{4½}^{{}^{\circ}}$—*e* ^8^P_4½_	*7 b*
2584.540	2	38680.04	−0.01	*a* ^4^P_0½_—*w* ${\;}^{2}D_{1½}^{{}^{\circ}}$	
2587.100	1*h*	38641.77	−0.04	*^a^* ^6^D_3½_— $55923_{2½}^{{}^{\circ}}$	
2592.298	6	38564.29	0.01	*a* ^4^G_2½_—*x* ${\;}^{2}D_{1½}^{{}^{\circ}}$	
2592.944	60R	38554.68	−0.01	*^z^* ${\;}^{8}P_{3½}^{{}^{\circ}}$—*e* ^8^P_2½_	4
2595.763	80 R	38512.81	0.03	*z* ${\;}^{8}P_{4½}^{{}^{\circ}}$—*e* ^8^P_3½_	
2597.722	2	38483.77	−0.09	*a* ^4^G_2½_—*x* ${\;}^{2}D_{2½}^{{}^{\circ}}$	
2598.172	6	38477.11	−0.05	*a* ^4^G_3½_—*x* ${\;}^{2}D_{2½}^{{}^{\circ}}$	
2600.220	8H*w*	38446.80	0.00	*a* ^6^D_4½_—*y* ${\;}^{8}F_{5½}^{{}^{\circ}}$	
2600.650	6H*w*	38440.45	0.00	*a* ^6^D_4½_—*v* ${\;}^{6}F_{5½}^{{}^{\circ}}$	
2601.486	2	38428.09	−0.05	*a* ^6^D_1½_—*s* ${\;}^{6}P_{1½}^{{}^{\circ}}$	
2603.487	1H*w*	38398.57	−0.3	*z* ${\;}^{8}P_{2½}^{{}^{\circ}}$—*h* ^8^D	
2606.137	1H	38359.52	0.05	*a* ^6^D_0½_—*s* ${\;}^{6}P_{1½}^{{}^{\circ}}$	
2612.233	5H*w*	38270.01	0.2	*z* ${\;}^{8}P_{3½}^{{}^{\circ}}$—*h* ^8^D	
2612.860	20	38260.83	0.08	*a* ^4^G_3½_—*z* ${\;}^{2}H_{4½}^{{}^{\circ}}$	
2613.006	1	38258.69	0.13	*a* ^4^G_3½_— $63546_{3½}^{{}^{\circ}}$	
2614.550	3	38236.10	0.02	*a* ^4^G_3½_— $63523_{2½}^{{}^{\circ}}$	
2615.850	5H*w*	38217.10	0.01	*a* ^6^D_3½_—*y* ${\;}^{8}F_{4½}^{{}^{\circ}}$	
2616.300	4H*w*	38210.52	0.00	*a* ^6^D_3½_—*v* ${\;}^{6}F_{4½}^{{}^{\circ}}$	
2617.564	1	38192.07	−0.04	*a* ^4^G_5½_—*w* ${\;}^{4}H_{5½}^{{}^{\circ}}$	
2618.470	4	38178.86	−0.01	*a* ^4^G_5½_—*w* ${\;}^{4}H_{4½}^{{}^{\circ}}$	
2618.911	20	38172.43	0.01	*a* ^4^G_4½_—*w* ${\;}^{4}H_{5½}^{{}^{\circ}}$	
2619.510	25	38163.70	0.00	*a* ^4^G_4½_—*y* ${\;}^{2}H_{5½}^{{}^{\circ}}$	
2619.819	5	38159.20	0.02	*a* ^4^G_4½_—*w* ${\;}^{4}H_{4½}^{{}^{\circ}}$	
2619.980	10	38156.86	−0.01	*a* ^4^G_3½_—*w* ${\;}^{4}H_{4½}^{{}^{\circ}}$	
2622.895	25	38114.45	0.04	*a* ^4^G_2½_—*w* ${\;}^{4}H_{3½}^{{}^{\circ}}$	
2623.284	8	38108.80	0.01	*a* ^4^G_5½_— $63374_{4½}^{{}^{\circ}}$	
2623.362	5	38107.67	−0.04	*a* ^4^G_3½_—*w* ${\;}^{4}H_{3½}^{{}^{\circ}}$	
2624.043	50	38097.78	−0.02	*a* ^4^G_5½_—*w* ${\;}^{4}H_{6½}^{{}^{\circ}}$	4
2624.466	1H*w*	38096.2	0.2	*z* ${\;}^{8}P_{4½}^{{}^{\circ}}$—*h* ^8^D	
2624.642	2	38089.08	−0.02	*a* ^4^G_4½_— $63374_{4½}^{{}^{\circ}}$	
2624.800	10	38086.79	0.00	*a* ^4^G_3½_— $63374_{4½}^{{}^{\circ}}$	
2625.120	3	38082.15	−0.02	*a* ^4^G_5½_—*y* ${\;}^{2}H_{4½}^{{}^{\circ}}$	
2626.635	20	38060.19	0.02	*a* ^4^D_3½_—*y* ${\;}^{2}H_{4½}^{{}^{\circ}}$	
2627.48	1H*w*	38048.0	0.0	*a* ^6^D_2½_—*y* ${\;}^{8}F_{3½}^{{}^{\circ}}$	
2627.990	} 2H*w*	38040.56	0.00	*a* ^6^D_2½_—*v* ${\;}^{6}F_{3½}^{{}^{\circ}}$	
2628.100		38038.97	0.16	*a* ^4^G_2½_— $63319_{3½}^{{}^{\circ}}$	
2630.260	8	38007.74	0.00	*a* ^4^G_2½_—*x* ${\;}^{2}F_{3½}^{{}^{\circ}}$	
2630.565	25	38003.33	−0.02	*a* ^4^G_4½_—*z* ${\;}^{2}H_{5½}^{{}^{\circ}}$	
2630.721	2	38001.08	0.04	*a* ^4^G_3½_—*x* ${\;}^{2}F_{3½}^{{}^{\circ}}$	
2635.551	4H*w*	37931.42	0.00	*a* ^6^D_1½_—*y* ${\;}^{8}F_{2½}^{{}^{\circ}}$	
2636.131	2H*w*	37923.09	0.00	*a* ^6^D_1½_—*v* ${\;}^{6}F_{2½}^{{}^{\circ}}$	
2640.340	1H*w*	37862.6	0.0	*a* ^6^D_0½_—*y* ${\;}^{8}F_{1½}^{{}^{\circ}}$	
2640.619	4	37858.64	−0.02	*a* ^4^G_2½_—*x* ${\;}^{2}F_{2½}^{{}^{\circ}}$	
2640.887	1H*w*	37854.80	0.00	*a* ^6^D_0½_—*v* ${\;}^{6}F_{1½}^{{}^{\circ}}$	
2642.403	3	37833.08	0.01	*a* ^4^G_2½_—*y* ${\;}^{2}D_{1½}^{{}^{\circ}}$	
2645.168	1	37793.54	0.00	*a* ^4^G_3½_—*y* ${\;}^{2}D_{2½}^{{}^{\circ}}$	
2648.800	3H*w*	37741.72	0.02	*z* ${\;}^{8}P_{2½}^{{}^{\circ}}$—*g* ^8^S_3½_	
2654.824	1	37656.09	0.09	*a* ^6^D_2½_—*x* ${\;}^{4}D_{3½}^{{}^{\circ}}$	
2655.787	10*h*	37642.43	0.13	*a* ^4^D_3½_—*w* ${\;}^{4}F_{4½}^{{}^{\circ}}$	
2657.528	1	37617.77	0.08	*a* ^6^D_1½_—*x* ${\;}^{4}D_{2½}^{{}^{\circ}}$	
2657.898	8H*l*	37612.54	−0.05	*z* ${\;}^{8}P_{3½}^{{}^{\circ}}$ —*g* ${\;}^{8}S_{3½}^{{}^{\circ}}$	
3658.349	2	37606.16	0.03	*a* ^4^D_3½_—*w* ${\;}^{4}F_{3½}^{{}^{\circ}}$	
2661.20	2 *h*	37566.3	0.3	*a* ^4^G_4½_—*w* ${\;}^{6}D_{3½}^{{}^{\circ}}$	
2665.064	4*h*	37511.41	0.01	*a* ^4^P_2½_—*u* ${\;}^{4}D_{2½}^{{}^{\circ}}$	
2667.263	1	37480.49	0.20	*a* ^4^G_2½_—*w* ${\;}^{6}D_{2½}^{{}^{\circ}}$	
2667.751	1	37473.63	0.04	*a* ^4^G_3½_—*w* ${\;}^{6}D_{2½}^{{}^{\circ}}$	
2667.882	1	37471.79	0.04	*b* ^4^D_2½_—*t* ${\;}^{4}G_{3½}^{{}^{\circ}}$	
2668.370	3*h*	37464.94	$\{\begin{array}{c} 0.00\\ -0.02 \end{array}$	*a* ^4^P_1½_—*u* ${\;}^{4}D_{2½}^{{}^{\circ}}$ *b* ^4^D_3½_—*t* ${\;}^{4}G_{4½}^{{}^{\circ}}$	
2670.237	4H*w*	37438.74	−0.05	*z* ${\;}^{8}P_{4½}^{{}^{\circ}}$—*g* ${\;}^{8}S_{3½}^{{}^{\circ}}$	
2670.435	4H*w*	37435.97	0.02	*a* ^4^P_1½_—*u* ${\;}^{4}D_{1½}^{{}^{\circ}}$	
2672.85	1	37402.2	0.1	*a* ${\;}^{4}P_{1½}^{{}^{\circ}}$—*u* ${\;}^{4}D_{1½}^{{}^{\circ}}$	
2673.651	1	37390.95	0.27	*a* ^4^P_1½_—*u* ${\;}^{4}D_{0½}^{{}^{\circ}}$	
2676.090	3	37356.87	0.04	*a* ^4^P_0½_—*u* ${\;}^{4}D_{0½}^{{}^{\circ}}$	
2676.326	10	37353.57	− 0.03	*a* ^4^D_2½_—*w* ${\;}^{4}F_{3½}^{{}^{\circ}}$	
2682.244	2	37271.16	0.01	*a* ^4^D_2½_—*w* ${\;}^{4}F_{2½}^{{}^{\circ}}$	
2685.941	8H	37219.86	0.00	*a* ^4^G_4½_—*v* ${\;}^{4}F_{3½}^{{}^{\circ}}$	
2686.777	6	37208.28	0.13	*a* ^4^G_2½_—*u* ${\;}^{4}D_{3½}^{{}^{\circ}}$	
2687.400	8*h*	37199.66	0.04	*a* ^4^G_3½_—*v* ${\;}^{4}F_{2½}^{{}^{\circ}}$	
2688.078	2	37190.28	0.06	*a* ^4^D_2½_—*z* ${\;}^{2}G_{3½}^{{}^{\circ}}$	
2690.977	2	37150.21	−0.05	*z* ${\;}^{8}P_{3½}^{{}^{\circ}}$—*h* ^6^D_4½_	
2692.655	20	37127.06	−0.02	*a* ^4^G_5½_—*v* ${\;}^{8}F_{4½}^{{}^{\circ}}$	
2693.757	2	37109.12	−0.04	*a* ^4^G_2½_—*v* ${\;}^{4}F_{1½}^{{}^{\circ}}$	
2694.560	8	37100.82	−0.01	*a* ^4^D_1½_—*w* ${\;}^{4}F_{2½}^{{}^{\circ}}$	
2698.890	2	37041.30	−0.05	*a* ^4^D_1½_—*w* ${\;}^{4}F_{1½}^{{}^{\circ}}$	
2703.129	20H *w*	36983.21	0.48	*z* ${\;}^{8}P_{4½}^{{}^{\circ}}$—*h* ^6^D_3½_	
2703.658	50H *w*	36975.96	−0.57	*z* ${\;}^{8}P_{4½}^{{}^{\circ}}$—*h* ^6^D_4½_	
2703.92	40H *w*	36972.4	0.1	*z* ${\;}^{8}P_{2½}^{{}^{\circ}}$—*g* ^8^D_3½_	
2706.142	5	36942.04	0.04	*a* ^4^D_0½_— *w* ${\;}^{4}F_{1½}^{{}^{\circ}}$	
2713.320	100H *w*	36844.31	0.25	*z* ${\;}^{8}P_{3½}^{{}^{\circ}}$—*g* ^8^D_4½_	
2717.028	4	36794.04	0.06	*a* ^4^G_2½_—*y* ${\;}^{2}F_{2½}^{{}^{\circ}}$	
2720.387	2*h*	36748.61	0.00	*a* ^4^G_4½_—*y* ${\;}^{2}F_{3½}^{{}^{\circ}}$	
2720.550	*5h*	36746.41	0.11	*a* ^4^G_3½_—*y* ${\;}^{2}F_{3½}^{{}^{\circ}}$	
2726.13	100H*w*	36671.2	−0.1	*z* ${\;}^{8}P_{4½}^{{}^{\circ}}$—*g* ^8^D_5½_	
2727.381	1	36654.37	0.04	*b* ^4^D_3½_— $67008_{2½}^{{}^{\circ}}$	
2729.420	10	36627.00	−0.09	*b* ^4^D_3½_— $66981_{3½}^{{}^{\circ}}$	
2732.260	2	36588.93	0.00	*b* ^4^D_2½_— $67008_{2½}^{{}^{\circ}}$	
2733.167	1	36576.78	0.01	*a* ^4^D_0½_—*z* ${\;}^{2}D_{1½}^{{}^{\circ}}$	
2734.164	1	36563.45	$\{\begin{array}{c} 0.09\\ -0.02 \end{array}$	*a* ^4^P_2½_—*x* ${\;}^{2}D_{2½}^{{}^{\circ}}$ *a* ^4^P_0½_—*x* ${\;}^{2}D_{1½}^{{}^{\circ}}$	
2734.297	1	36561.67	−0.02	*b* ^4^D_2½_— $66981_{3½}^{{}^{\circ}}$	
2734.736	15	36555.80	−0.01	*b* ^4^D_3½_— $66910_{2½}^{{}^{\circ}}$	
2734.997	4	36552.31	−0.14	*a* ^4^D_2½_—*z* ${\;}^{2}D_{2½}^{{}^{\circ}}$	
2735.310	1*hw*	36548.13	−0.07	*z* ${\;}^{8}P_{2½}^{{}^{\circ}}$—*g* ^6^D_2½_	
2737.640	2	36517.03	0.13	*a* ^4^P_1½_—*x* ${\;}^{2}D_{2½}^{{}^{\circ}}$	
2738.552	5	36504.87	−0.03	*a* ^4^G_2½_—*y* ${\;}^{2}G_{3½}^{{}^{\circ}}$	
2738.861	25	36500.75	−0.04	*b* ^4^D_3½_—*u* ${\;}^{4}F_{4½}^{{}^{\circ}}$	
2739.627	4	36490.54	0.13	*b* ^4^D_2½_— $66910_{2½}^{{}^{\circ}}$	
2740.161	1*h*	36483.43	0.00	*b* ^4^D_3½_—*u* ${\;}^{4}F_{2½}^{{}^{\circ}}$	
2740.546	3H*w*	36478.31	0.01	*a* ^4^G_5½_— $61744_{5½}^{{}^{\circ}}$	
2742.735	15	36449.19	0.41	*a* ^4^G_5½_—*y* ${\;}^{2}G_{4½}^{{}^{\circ}}$?	
2742.930	1	36446.60	0.18	*a* ^4^G_2½_—*z* ${\;}^{2}F_{2½}^{{}^{\circ}}$	
2743.446	10	36439.75	0.03	*a* ^4^G_3½_—*z* ${\;}^{2}F_{2½}^{{}^{\circ}}$	
2743.790	5	36435.18	−0.09	*a* ^4^D_3½_—*x* ${\;}^{4}G_{4½}^{{}^{\circ}}$	
2744.023	9	36432.08	0.03	*b* ^4^D_0½_—*u* ${\;}^{4}F_{1½}^{{}^{\circ}}$	
2744.268	6*h*	36428.84	$\{\begin{array}{c} -0.25\\ 0.00 \end{array}$	*a* ^4^G_4½_—*y* ${\;}^{2}G_{4½}^{{}^{\circ}}$ *b* ^4^D_3½_—*u* ${\;}^{4}F_{3½}^{{}^{\circ}}$	
2744.519	6	36425.51	−0.04	*a* ^4^G_4½_—*z* ${\;}^{2}F_{3½}^{{}^{\circ}}$	
2744.748	1	36422.46	0.00	*a* ^4^D_1½_—*w* ${\;}^{4}F_{0½}^{{}^{\circ}}$	
2745.082	3	36418.03	$\{\begin{array}{c} 0.00\\ -0.05 \end{array}$	*b* ^4^D_2½_—*u* ${\;}^{4}F_{2½}^{{}^{\circ}}$ *b* ^4^D_1½_—*u* ${\;}^{4}F_{1½}^{{}^{\circ}}$	
2745.549	10	36411.84	−0.09	*b* ^4^D_1½_—*u* ${\;}^{4}F_{2½}^{{}^{\circ}}$	
2745.913	2*h*	36407.01	0.09	*z* ${\;}^{8}P_{3½}^{{}^{\circ}}$—*g* ^6^D_4½_	
2747.785	4*d*	36382.21	$\{\begin{array}{c} 0.08\\ -0.09 \end{array}$	*a* ^4^D_1½_—*z* ${\;}^{2}D_{2½}^{{}^{\circ}}$ *a* ^4^P_2½_— $63583_{2½,\;1½}^{{}^{\circ}}$	
2749.205	20*h*	36363.42	−0.02	*b* ^4^D_2½_—*u* ${\;}^{4}F_{3½}^{{}^{\circ}}$	
2750.602	3	36344.95	0.19	*a* ^4^P_2½_— $63546_{3½}^{{}^{\circ}}$	
2752.259	3	36323.07	−0.04	*a* ^4^D_0½_—*w* ${\;}^{4}D_{0½}^{{}^{\circ}}$	
2752.322	7	36322.24	−0.04	*a* ^4^P_2½_— $63523_{2½}^{{}^{\circ}}$	
2752.635	3	36318.10	−0.11	*b* ^4^D_2½_—*v* ${\;}^{2}G_{3½}^{{}^{\circ}}$	
2753.851	3	36302.07	0.08	*a* ^4^P_0½_— $63583_{2½,\;1½}^{{}^{\circ}}$	
2753.995	3H*w*	36300.18	−0.26	*b* ^4^D_3½_— $66654_{2½}^{{}^{\circ}}$	
2755.777	2	36276.70	−0.01	*b* ^4^D_3½_—*v* ${\;}^{2}G_{4½}^{{}^{\circ}}$	
2755.839	2	36275.88	0.06	*a* ^4^P_1½_— $63523_{2½}^{{}^{\circ}}$	
2756.267	4	36270.25	0.00	*a* ^4^D_1½_—*w* ${\;}^{4}D_{1½}^{{}^{\circ}}$	
2758.950	4	36234.98	$\{\begin{array}{c} -0.06\\ -0.13 \end{array}$	*b* ^4^D_2½_— $66654_{2½}^{{}^{\circ}}$ *a* ^4^D_2½_—*x* ${\;}^{4}G_{3½}^{{}^{\circ}}$	
2760.920	100 *hl*	36209.13	0.00	*a* ^6^D_4½_—*t* ${\;}^{6}P_{3½}^{{}^{\circ}}$	
2761.350	4	36203.50	0.03	*a* ^4^G_5½_—*w* ${\;}^{4}G_{5½}^{{}^{\circ}}$	
2761.797	3	36197.64	0.04	*a* ^4^G_3½_—*w* ${\;}^{4}G_{4½}^{{}^{\circ}}$	
2761.985	2*h*	36195.17	0.00	*a* ^4^G_4½_—*w* ${\;}^{4}G_{3½}^{{}^{\circ}}$	
2762.853	3*h*	36183.80	0.02	*a* ^4^G_4½_—*w* ${\;}^{4}G_{5½}^{{}^{\circ}}$	
2763.665	3	36173.17	−0.30	*a* ^4^G_3½_—*v* ${\;}^{4}D_{3½}^{{}^{\circ}}$	
2763.87	3	36170.4	−0.5	*a* ^4^D_0½_—*w* ${\;}^{4}D_{1½}^{{}^{\circ}}$	
2763.907	8*h*	36170.00	−0.02	*a* ^4^P_2½_— $63371_{2½}^{{}^{\circ}}$	
2764.027	1	36168.44	0.03	*b* ^4^D_3½_—*u* ${\;}^{4}G_{4½}^{{}^{\circ}}$	
2767.450	10	36123.70	0.14	*a* ^4^P_1½_— $63371_{2½}^{{}^{\circ}}$	
2769.410	2	36098.13	−0.05	*a* ^4^D_1½_—*x* ${\;}^{4}G_{2½}^{{}^{\circ}}$	
2770.242	3	36087.30	0.06	*a* ^4^P_2½_—*x* ${\;}^{2}F_{3½}^{{}^{\circ}}$	
2771.430	30	36071.83	0.03	*a* ^6^D_4½_—*y* ${\;}^{4}D_{3½}^{{}^{\circ}}$	
2772.032	2	36063.99	−0.06	*a* ^4^D_3½_—*x* ${\;}^{4}F_{2½}^{{}^{\circ}}$	
2773.021	5	36051.13	−0.02	*a* ^4^D_2½_—*w* ${\;}^{4}D_{2½}^{{}^{\circ}}$	
2773.659	10	36042.84	−0.04	*a* ^4^D_3½_—*w* ${\;}^{4}D_{3½}^{{}^{\circ}}$	
2774.289	1	36034.66	0.00	*b* ^4^D_2½_—*u* ${\;}^{4}G_{3½}^{{}^{\circ}}$	
2776.218	80 *hl*	36009.62	0.04	*a* ^6^D_3½_—*t* ${\;}^{6}P_{2½}^{{}^{\circ}}$	
2777.464	6	35993.47	−0.09	*a* ^4^D_3½_—*x* ${\;}^{4}F_{3½}^{{}^{\circ}}$	
2778.544	60*h*	35979.47	0.05	*a* ^6^D_3½_—*t* ${\;}^{6}P_{3½}^{{}^{\circ}}$	
2779.993	40	35960.72	−0.05	*a* ^4^D_3½_—*x* ${\;}^{4}F_{4½}^{{}^{\circ}}$	
2781.733	1	35938.23	0.07	*a* ^4^p_2½_—*x* ${\;}^{2}F_{2½}^{{}^{\circ}}$	
2782.259	1	35931.44	−0.16	*a* ^4^D_2½_—*v* ${\;}^{4}D_{2½}^{{}^{\circ}}$	
2782.711	50H	35925.60	0.00	*a* ^6^D_4½_—*w* ${\;}^{6}F_{5½}^{{}^{\circ}}$	
2783.080	10	35920.84	−0.10	*a* ^4^D_2½_—*v* ${\;}^{4}F_{3½}^{{}^{\circ}}$	
2785.334	1	35891.77	0.07	*a* ^4^D_1½_—*x* ${\;}^{2}F_{2½}^{{}^{\circ}}$	
2786.185	3	35880.81	−0.02	*a* ^4^D_1½_—*w* ${\;}^{4}D_{2½}^{{}^{\circ}}$	
2786.266	3	35879.77	0.03	*a* ^4^P_2½_—*y* ${\;}^{2}D_{2½}^{{}^{\circ}}$	
2787.264	2	35866.93	−0.02	*a* ^4^D_2½_—*x* ${\;}^{4}F_{1½}^{{}^{\circ}}$	
2787.813	15*h*	35859.86	0.01	*a* ^6^D_2½_—*t* ${\;}^{6}P_{1½}^{{}^{\circ}}$	
2788.682	3	35848.68	−0.09	*a* ^4^D_1½_—*u* ${\;}^{4}P_{0½}^{{}^{\circ}}$	
2789.192	25	35842.13	0.04	*a* ^6^D_3½_—*y* ${\;}^{4}D_{3½}^{{}^{\circ}}$	6
2789.355	15*hw*	35840.03	−0.03	*a* ^6^D_2½_—*t* ${\;}^{6}P_{2½}^{{}^{\circ}}$	
2789.731	3	35835.20	−0.05	*a* ^4^D_2½_—*u* ${\;}^{4}P_{1½}^{{}^{\circ}}$	
2790.353	30	35827.21	0.00	*a* ^6^D_3½_—*y* ${\;}^{4}D_{2½}^{{}^{\circ}}$	
2790.925	4	35819.87	−0.06	*a* ^4^D_3½_—*u* ${\;}^{4}P_{2½}^{{}^{\circ}}$	
2791.085	20	35817.82	0.01	*a* ^6^D_4½_—*x* ${\;}^{6}D_{3½}^{{}^{\circ}}$	
2791.584	4	35811.42	−0.10	*a* ^4^D_2½_—*x* ${\;}^{4}F_{2½}^{{}^{\circ}}$	
2791.707	*2h*	35809.84	−0.06	*a* ^6^D_2½_—*t* ${\;}^{6}P_{3½}^{{}^{\circ}}$	
2793.24	6*h*	35790.2	−0.2	*a* ^4^D_2½_—*w* ${\;}^{4}D_{3½}^{{}^{\circ}}$	
2794.817	10000R	35769.99	0.02	*a* ^6^S_2½_—*y* ${\;}^{6}P_{3½}^{{}^{\circ}}$	4
2796.938	5	35742.88	−0.01	*a* ^6^D_1½_—*t* ${\;}^{6}P_{1½}^{{}^{\circ}}$	
2797.194	3	35740.88	−0.15	*a* ^4^D_2½_—*x* ${\;}^{4}F_{3½}^{{}^{\circ}}$	
2798.270	8000R	35725.86	0.01	*a* ^6^S_2½_—*y* ${\;}^{6}P_{2½}^{{}^{\circ}}$	6
2799.841	50	35705.81	−0.01	*a* ^6^D_4½_—*x* ${\;}^{6}D_{4½}^{{}^{\circ}}$	6,7 *b*
2800.63	6H	35695.75	0.00	*a* ^6^D_3½_—*w* ${\;}^{6}F_{4½}^{{}^{\circ}}$	
2801.084	6000R	35689.97	−0.01	*a* ^6^S_2½_—*y* ${\;}^{6}P_{1½}^{{}^{\circ}}$	4
2802.168	10	35676.16	−0.01	*a* ^4^G_2½_—*x* ${\;}^{2}H_{3½}^{{}^{\circ}}$	
2802.399	5	35673.22	−0.01	*a* ^4^G_5½_—*w* ${\;}^{4}F_{4½}^{{}^{\circ}}$	
2802.454	10	35672.51	−0.06	*a* ^4^D_2½_—*y* ${\;}^{4}D_{3½}^{{}^{\circ}}$	
2802.619	3	35670.42	−0.03	*a* ^4^G_4½_—*x* ${\;}^{4}H_{4½}^{{}^{\circ}}$	
2802.697	2	35669.43	−0.04	*a* ^4^G_3½_—*x* ${\;}^{4}H_{3½}^{{}^{\circ}}$	
2802.805	15	35668.06	−0.08	*a* ^4^G_3½_—*x* ${\;}^{4}H_{4½}^{{}^{\circ}}$	
2803.623	10	35657.65	−0.04	*a* ^6^D_2½_—*y* ${\;}^{4}D_{2½}^{{}^{\circ}}$	
2803.946	2	35653.54	0.00	*a ^4^G*_4½_—*w* ${\;}^{4}F_{4½}^{{}^{\circ}}$	
2804.095	20	35651.64	−0.03	*a* ^6^D_2½_—*y* ${\;}^{4}D_{1½}^{{}^{\circ}}$	
2804.216	2	35649.98	0.05	*a* ^4^P_2½_—*w* ${\;}^{6}D_{3½}^{{}^{\circ}}$	
2804.363	15	35648.24	−0.06	*a* ^4^G_4½_—*x* ${\;}^{4}H_{5½}^{{}^{\circ}}$	
2804.929	6	35641.05	−0.15	*a* ^4^D_1½_—*x* ${\;}^{4}F_{2½}^{{}^{\circ}}$	
2806.136	30	35625.72	−0.02	*a* ^4^G_5½_—*x* ${\;}^{4}H_{6½}^{{}^{\circ}}$	
2806.794	10	35617.37	0.00	*a* ^4^G_4½_—*w* ${\;}^{4}F_{3½}^{{}^{\circ}}$	
2806.977	4	35615.04	−0.02	*a* ^4^G_3½_—*w* ${\;}^{4}F_{3½}^{{}^{\circ}}$	
2808.015	20	35601.88	0.01	*a* ^6^D_3½_—*x* ${\;}^{6}D_{2½}^{{}^{\circ}}$	
2808.385	8	35597.19	−0.09	*a* ^4^D_0½_—*x* ${\;}^{4}F_{1½}^{{}^{\circ}}$	
2809.103	25	35588.09	−0.01	*a* ^6^D_3½_—*x* ${\;}^{6}D_{3½}^{{}^{\circ}}$	
2811.337	4	35559.81	0.02	*a* ^4^P_2½_—*w* ${\;}^{6}D_{2½}^{{}^{\circ}}$	
2812.090	1	35550.29	0.13	*b* ^4^D_0½_—*w* ${\;}^{2}D_{1½}^{{}^{\circ}}$	
2812.840	20	35540.82	0.09	*a* ^6^D_1½_—*y* ${\;}^{4}D_{2½}^{{}^{\circ}}$	
2812.933	2	35539.64	0.01	*a* ^4^P_1½_—*w* ${\;}^{6}D_{1½}^{{}^{\circ}}$	
2813.489	20	35532.61	$\{\begin{array}{c} -0.23\\ 0.00 \end{array}$	*a* ^6^D_1½_—*y* ${\;}^{4}D_{0½}^{{}^{\circ}}$ *a* ^4^G_3½_—*w* ${\;}^{4}F_{2½}^{{}^{\circ}}$	
2813.989	1211	35526.30	0.00	*a* ^6^D_2½_—*w* ${\;}^{6}F_{3½}^{{}^{\circ}}$	
2814.462	2	35520.33	0.17	*a* ^4^P_1½_—*w* ${\;}^{6}D_{0½}^{{}^{\circ}}$	
2815.018	8	35513.32	−0.01	*a* ^4^P_1½_—*w* ${\;}^{6}D_{2½}^{{}^{\circ}}$	
2815.609	8*h*	35505.86	0.08	*a* ^4^P_0½_—*w* ${\;}^{6}D_{1½}^{{}^{\circ}}$	
2817.164	*5h*	35486.27	−0.04	*a* ^4^P_0½_—*w* ${\;}^{6}D_{0½}^{{}^{\circ}}$	
2817.667	10	35479.93	0.10	*a* ^4^G_2½_—*w* ${\;}^{4}F_{1½}^{{}^{\circ}}$	
2817.969	30*l*	35476.13	0.02	*a* ^6^D_3½_—*x* ${\;}^{6}D_{4½}^{{}^{\circ}}$	
2818.770	20	35466.05	0.01	*a* ^6^G_0½_—*y* ${\;}^{4}D_{1½}^{{}^{\circ}}$	
2818.919	10	35464.17	0.00	*a* ^6^D_0½_—*y* ${\;}^{4}D_{0½}^{{}^{\circ}}$	
2819.326	4	35458.42	0.04	*a* ^4^G_2½_—*z* ${\;}^{2}G_{3½}^{{}^{\circ}}$	
2819.727	4	35454.01	0.02	*a* ^4^G_4½_—*z* ${\;}^{2}G_{3½}^{{}^{\circ}}$	
2821.452	20	35432.34	−0.01	*a* ^6^D_2½_—*x* ${\;}^{6}D_{2½,\;1½}^{{}^{\circ}}$	
2822.275	2*h*	35422.00	0.00	*a* ^4^H_6½_—*x* ${\;}^{2}I_{6½}^{{}^{\circ}}$	
2822.549	30	35418.57	−0.01	*a* ^6^D_2½_—*x* ${\;}^{6}D_{3½}^{{}^{\circ}}$	
2823.268	5H	35409.55	0.00	*a* ^6^D_1½_—*w* ${\;}^{6}F_{2½}^{{}^{\circ}}$	
2823.813	1	35402.71	−0.04	*a* ^4^G_5½_—*z* ${\;}^{6}G_{4½}^{{}^{\circ}}$	
2825.552	2	35380.86	0.11	*a* ^4^G_3½_—*z* ${\;}^{2}G_{4½}^{{}^{\circ}}$	
2828.762	6H	35340.78	0.00	*a* ^6^D_0½_—*w* ${\;}^{6}F_{1½}^{{}^{\circ}}$	
2830.793	20	35315.42	0.03	*a* ^6^D_1½_—*x* ${\;}^{6}D_{2½\;,1½}^{{}^{\circ}}$	
2833.171	3*h*	35285.78	−0.04	*a* ^4^P_2½_—*v* ${\;}^{4}F_{2½}^{{}^{\circ}}$	
2836.310	20	35246.74	0.02	*a* ^6^D_0½_—*x* ${\;}^{6}D_{1½}^{{}^{\circ}}$	
2836.898	4*h*	35239.43	0.07	*a* ^6^P_1½_—*v* ${\;}^{4}F_{2½}^{{}^{\circ}}$	
2839.997	15	35200.98	0.03	*a* ^6^D_4½_—*u* ${\;}^{6}P_{3½}^{{}^{\circ}}$	
2840.983	1	35188.76	0.10	*a* ^4^P_2½_—*v* ${\;}^{4}F_{1½}^{{}^{\circ}}$	
2844.764	2	35141.97	−0.23	*a* ^4^P_1½_—*v* ${\;}^{4}F_{1½}^{{}^{\circ}}$	
2858.655	30	34971.24	0.00	*a* ^6^D_3½_—*u* ${\;}^{6}P_{3½}^{{}^{\circ}}$	6
2863.827	2*h*	34908.09	0.02	*b* ^4^D_3½_—*w* ${\;}^{2}G_{4½}^{{}^{\circ}}$	
2866.646	2H	34873.76	0.28	*a* ^4^P_2½_—*y* ${\;}^{2}F_{2½}^{{}^{\circ}}$?	
2868.880	7	34846.60	−0.05	*a* ^6^D_3½_—*u* ${\;}^{6}P_{2½}^{{}^{\circ}}$	
2870.464	1*h*	34827.37	0.35	*a* ^4^P_1½_—*y* ${\;}^{2}F_{2½}^{{}^{\circ}}$?	
2871.583	1*h*	34813.78	0.21	*a* ^4^D_3½_—*y* ${\;}^{4}G_{4½}^{{}^{\circ}}$	
2872.583	30	34801.69	−0.03	*a* ^6^D_2½_—*u* ${\;}^{6}P_{3½}^{{}^{\circ}}$	5
2880.270	1*h*	34708.81	0.08	*a* ^4^G_2__½_—*w* ${\;}^{4}D_{1½}^{{}^{\circ}}$	
2882.899	20	34677.16	0.03	*a* ^6^D_2__½_—*u* ${\;}^{6}P_{2½}^{{}^{\circ}}$	
2890.388	1*h*	34587.31	−0.18	*a* ^4^D_2__½_—*y* ${\;}^{4}G_{3½}^{{}^{\circ}}$	
2891.945	2*h*	34568.69	0.08	*b* ^4^D_2__½_—*w* ${\;}^{2}F_{3½}^{{}^{\circ}}$	
2892.388	2	34563.39	−0.09	*a* ^6^D_2__½_—*u* ${\;}^{6}P_{1½}^{{}^{\circ}}$	
2892.493	5	34562.13	−0.01	*a* ^4^G_5__½_—*z* ${\;}^{2}I_{5½}^{{}^{\circ}}$	
2892.657	20	34560.19	0.02	*a* ^6^D_1__½_—*u* ${\;}^{6}P_{2½}^{{}^{\circ}}$	5
2894.625	10	34536.69	0.03	*a* ^4^G_2__½_—*x* ${\;}^{4}G_{2½}^{{}^{\circ}}$	
2895.188	8	34529.97	0.01	*a* ^4^G_3__½_—*x* ${\;}^{4}G_{2½}^{{}^{\circ}}$	
2897.428	5	34503.28	0.01	*a* ^4^G_2__½_—*x* ${\;}^{4}G_{3½}^{{}^{\circ}}$	
2897.651	1	34500.63	−0.09	*b* ^4^D_2__½_—*v* ${\;}^{4}H_{3½}^{{}^{\circ}}$	
2897.797	15	34498.89	0.01	*a* ^4^G_4__½_—*x* ${\;}^{4}G_{3½}^{{}^{\circ}}$	
2897.990	10	34496.59	0.02	*a* ^4^G_3__½_—*x* ${\;}^{4}G_{3½}^{{}^{\circ}}$	
2900.545	20	34466.20	0.00	*a* ^4^G_5__½_—*x* ${\;}^{4}G_{4½}^{{}^{\circ}}$	5
2902.203	25	34446.51	$\{\begin{array}{c} -0.01\\ 0.00 \end{array}$	*a* ^6^D_1__½_—*u* ${\;}^{6}P_{1½}^{{}^{\circ}}$ *a* ^4^G_4__½_—*X* ${\;}^{4}G_{4½}^{{}^{\circ}}$	
2902.399	5	34444.19	−0.01	*a* ^4^G_3__½_—*x* ${\;}^{4}G_{4½}^{{}^{\circ}}$	
2905.825	1*h*	34403.58	−0.02	*b* ^4^D_2__½_—*w* ${\;}^{2}F_{2½}^{{}^{\circ}}$	
2906.340	4*h*	34397.49	−0.01	*b* ^4^D_1__½_—*w* ${\;}^{3}F_{2½}^{{}^{\circ}}$	
2907.214	40	34387.15	−0.01	*a* ^4^G_5__½_—*x* ${\;}^{4}G_{5½}^{{}^{\circ}}$	6
2907.993	15	34377.93	0.08	*a* ^6^D_0__½_—*u* ${\;}^{6}P_{1½}^{{}^{\circ}}$	
2908.878	10	34367.47	0.00	*a* ^4^G_4__½_—*x* ${\;}^{4}G_{5½}^{{}^{\circ}}$	
2909.630	1	34358.59	−0.14	*b* ^4^D_3__½_—*u* ${\;}^{4}D_{2½}^{{}^{\circ}}$	
2910.242	3	34351.37	−0.01	*a* ^4^G_5__½_—*z* ${\;}^{2}I_{6½}^{{}^{\circ}}$	
2911.839	1*h*	34332.53	−0.02	*z* ${\;}^{8}P_{2½}^{{}^{\circ}}$—*f* ^6^D_1__½_	
2912.000	2*h*	34330.63	−0.13	*z* ${\;}^{8}P_{2½}^{{}^{\circ}}$—*f* ${\;}^{6}D_{2½}^{{}^{\circ}}$	
2912.226	3*h*	34327.97	0.02	*z* ${\;}^{8}P_{2½}^{{}^{\circ}}$—*f* ^6^D_3__½_	
2913.518	1	34312.74	0.13	*a* ^4^G_3__½_—*w* ${\;}^{4}D_{2½}^{{}^{\circ}}$	
2914.599	600*hw*	34300.02	0.00	*z* ${\;}^{8}P_{2½}^{{}^{\circ}}$—*f* ^8^D	
2916.375	1*h*	34279.13	0.07	*a* ^4^P_2__½_—*w* ${\;}^{4}G_{3½}^{{}^{\circ}}$	
2917.637	1	34264.31	−0.03	*b* ^4^D_2__½_—*u* ${\;}^{4}D_{1½}^{{}^{\circ}}$	
2919.122	8	34246.88	0.03	*a* ^4^G_2__½_—*v* ${\;}^{4}D_{1½}^{{}^{\circ}}$	
2920.458	8	34231.21	−0.02	*b* ^4^D_3__½_—*x* ${\;}^{2}G_{4½}^{{}^{\circ}}$	4
2920.599	5	34229.56	−0.03	*b* ^4^D_2__½_—*x* ${\;}^{2}G_{3½}^{{}^{\circ}}$	
2921.112	1	34223.55	0.32	*a* ^4^P_1__½_—*w* ${\;}^{4}G_{2½}^{{}^{\circ}}$	
2923.145	2	34199.75	−0.01	*a* ^4^G_2__½_—*v* ${\;}^{4}D_{2½}^{{}^{\circ}}$	
2923.229	2*h*	34198.76	−0.01	*z* ${\;}^{8}P_{3½}^{{}^{\circ}}$—*f* ^6^D_3__½_	
2923.577	3*h*	34194.69	−0.06	*z* ${\;}^{8}P_{3½}^{{}^{\circ}}$—*f* ^6^D_4__½_	
2923.715	10	34193.08	0.02	*a* ^4^G_3__½_—*v* ${\;}^{4}D_{2½}^{{}^{\circ}}$	
2924.430	10	34184.72	0.01	*a* ^4^G_4__½_—*v* ${\;}^{4}D_{3½}^{{}^{\circ}}$	
2924.629	2	34182.39	−0.01	*a* ^4^G_3__½_—*v* ${\;}^{4}D_{3½}^{{}^{\circ}}$	
2925.58	500*hw*	34171.3	−0.2	*z* ${\;}^{8}P_{3½}^{{}^{\circ}}$—*f* ^8^D_4__½_	P–B
2928.678	40	34135.14	0.03	*a* ^4^G_2__½_—*x* ${\;}^{4}F_{1½}^{{}^{\circ}}$	
2930.245	20	34116.89	0.00	*a* ^6^D_4__½_—*x* ${\;}^{6}F_{5½}^{{}^{\circ}}$	4
2933.442	3	34079.71	0.03	*a* ^4^G_2__½_—*x* ${\;}^{4}F_{2½}^{{}^{\circ}}$	
2934.020	30	34072.99	0.01	*a* ^4^G_3__½_—*x* ${\;}^{4}F_{2½}^{{}^{\circ}}$	
2935.643	15	34054.15	0.03	*a* ^4^G_4__½_—*w* ${\;}^{4}D_{3½}^{{}^{\circ}}$	4
2935.844	3	34051.82	0.01	*a* ^4^G_3__½_—*w* ${\;}^{4}D_{3½}^{{}^{\circ}}$	
2936.156	10	34048.20	0.00	*a* ^6^D_4__½_—*x* ${\;}^{6}F_{4½}^{{}^{\circ}}$	6
2938.496	2*h*	34021.09	0.07	*z* ${\;}^{8}P_{4½}^{{}^{\circ}}$—*f* ^6^D_4__½_	
2939.904	20	34004.80	0.00	*a* ^4^G_4__½_—*x* ${\;}^{4}F_{3½}^{{}^{\circ}}$	
2940.331	400*hw*	33999.86	0.00	*z* ${\;}^{8}P_{4½}^{{}^{\circ}}$—*f* ^8^D_5__½_	
2940.483	2	33997.91	0.2	*z* ${\;}^{8}P_{4½}^{{}^{\circ}}$—*f* ^8^D_4__½_	
2941.038	40	33991.69	−0.01	*a* ^4^G_5__½_—*x* ${\;}^{4}F_{4½}^{{}^{\circ}}$	4
2941.681	5	33984.26	−0.04	*a* ^6^D_1__½_—*x* ${\;}^{4}P_{0½}^{{}^{\circ}}$	
2942.740	8	33972.03	0.02	*a* ^4^G_4__½_—*x* ${\;}^{4}F_{4½}^{{}^{\circ}}$	
2943.550	1	33962.68	0.02	*a* ^6^D_4_**_½_—***x* ${\;}^{6}F_{3½}^{{}^{\circ}}$	
2947.634	3	33915.63	0.00	*a* ^6^D_0½_—*x* ${\;}^{4}P_{0½}^{{}^{\circ}}$	
2950.979	3	33877.19	0.02	*a* ^6^D_1__½_—*x* ${\;}^{4}P_{1½}^{{}^{\circ}}$	
2953.008	10	33853.91	0.02	*a ^6^*D_2__½_—*x* ${\;}^{4}P_{2½}^{{}^{\circ}}$	
2956.101	20	33818.49	0.00	*a* ^6^D_3__½_**—***x* ${\;}^{6}F_{4½}^{{}^{\circ}}$	4
2956.971	10	33808.54	0.04	*a* ^6^D_0__½_—*x* ${\;}^{4}P_{1½}^{{}^{\circ}}$	
2963.250	10	33736.90	−0.03	*a* ^6^D_1__½_—*x* ${\;}^{4}P_{2½}^{{}^{\circ}}$	6
2963.606	20	33732.85	−0.10	*a* ^6^D_3__½_—*x* ${\;}^{6}F_{3½}^{{}^{\circ}}$	
2966.374	1	33701.38	0.12	*a* ^4^P_2__½_—*w* ${\;}^{4}F_{3½}^{{}^{\circ}}$	
2970.956	4	33649.40	−0.02	*a* ^6^D_3__½_—*x* ${\;}^{6}F_{2½}^{{}^{\circ}}$	
2974.089	20	33613.96	0.00	*a* ^4^H_6__½_—*t* ${\;}^{4}G_{5½}^{{}^{\circ}}$	4
2976.588	1	33585.74	−0.01	*a* ^4^G_5__½_—*z* ${\;}^{4}I_{5½}^{{}^{\circ}}$	
2976.987	1	33581.23	0.00	*a* ^4^G_4__½_—*z* ${\;}^{4}I_{4½}^{{}^{\circ}}$	
2977.190	1	33578.95	0.03	*a* ^4^G_3__½_—*z* ${\;}^{4}I_{4½}^{{}^{\circ}}$	
2977.303	3	33577.67	0.02	*a* ^4^G_5__½_—*z* ${\;}^{4}I_{6½}^{{}^{\circ}}$	
2977.755	1	33572.57	0.22	*a* ^4^P_1__½_—*w* ${\;}^{4}F_{2½}^{{}^{\circ}}$	
2977.922	1	33570.69	0.3	*a* ^4^D_3__½_—*u* ^6^F°	
2978.114	8*h*	33568.53	−0.12	*a*^4^H_5½_—*t* ${\;}^{4}G_{4½}^{{}^{\circ}}$	
2978.333	2	33566.06	0.00	*a* ^4^G_4__½_—*z* ${\;}^{4}I_{5½}^{{}^{\circ}}$	
2978.566	15	33563.44	0.01	*a* ^6^D_2__½_—*x* ${\;}^{6}F_{3½}^{{}^{\circ}}$	
2979.994	8*h*	33547.35	−0.11	*a* ^4^H_4__½_—*t* ${\;}^{4}G_{3½}^{{}^{\circ}}$	
2980.493	2*h*	33541.74	0.14	*a* ^4^H_3__½_—*t* ${\;}^{4}G_{2½}^{{}^{\circ}}$	
2984.002	2	33502.29	−0.03	*a* ^4^H_5__½_—*t* ${\;}^{4}G_{5½}^{{}^{\circ}}$	
2985.992	20	33479.97	0.07	*a* ^6^D_2__½_—*x* ${\;}^{6}F_{2½}^{{}^{\circ}}$	6
2986.407	2*h*	33475.32	0.05	*a* ^4^H_4__½_—*t* ${\;}^{4}G_{4½}^{{}^{\circ}}$	
2987.052	1*h*	33468.10	0.01	*a* ^4^H_3__½_—*t* ${\;}^{4}G_{3½}^{{}^{\circ}}$	
2990.135	1	33433.58	0.00	*b* ^4^D_0½_—*_x_* ${\;}^{2}D_{1½}^{{}^{\circ}}$	
2991.387	1	33419.59	−0.02	*b* ^4^D_1__½_—*x* ${\;}^{2}D_{1½}^{{}^{\circ}}$	
2992.109	5	33411.53	0.00	*a* ^6^D_2__½_—*x* ${\;}^{6}F_{1½}^{{}^{\circ}}$	
2996.183	2	33366.10	0.08	*a* ^4^H_6__½_—*w* ${\;}^{2}H_{5½}^{{}^{\circ}}$	
2996.470	10	33362.90	−0.04	*a* ^6^D_1__½_—*x* ${\;}^{6}F_{2½}^{{}^{\circ}}$	4
2997.826	6H	33347.81	0.07	*a* ^4^F_4__½_—*u* ${\;}^{2}G_{4½}^{{}^{\circ}}$	6, 7*b*
2998.056	1	33345.25	−0.04	*b* ^4^D_2__½_*—x* ${\;}^{2}D_{2½}^{{}^{\circ}}$	
2998.600	1*h*	33339.20	0.01	*b* ^4^D_1__½_—*x* ^2^D_2__½_	
3002.378	2*hw*	33297.25	0.03	*a* ^4^F_3__½_—*u* ${\;}^{2}G_{3½}^{{}^{\circ}}$	
3002.616	20	33294.61	0.04	*a* ^6^D_1½_—*x* ${\;}^{6}F_{1½}^{{}^{\circ}}$	6
3003.125	2	33288.97	−0.04	*a* ^6^D_4__½_—*y* ${\;}^{4}F_{4½}^{{}^{\circ}}$	
3006.629	4	33250.18	0.02	*a* ^6^D_1__½_—*x* ${\;}^{6}F_{0½}^{{}^{\circ}}$	
3007.102	7H	33244.95	−0.12	*a* ^4^F_3__½_—*u* ${\;}^{2}G_{4½}^{{}^{\circ}}$	
3007.650	80	33238.89	0.03	*a* ^4^G_2__½_—*y* ${\;}^{4}H_{3½}^{{}^{\circ}}$	5
3008.050	3	33234.47	0.00	*a* ^4^G_4__½_—*y* ${\;}^{4}H_{3½}^{{}^{\circ}}$	
3008.258	40	33232.17	0.01	*a* ^4^G_3__½_—*y* ${\;}^{4}H_{3½}^{{}^{\circ}}$	
3008.822	4	33225.95	0.05	*a* ^6^D_0__½_—*x* ${\;}^{6}F_{1½}^{{}^{\circ}}$	
3009.378	5	33219.80	0.02	*a* ^4^G_5__½_—*y* ${\;}^{4}H_{4½}^{{}^{\circ}}$	5
3011.165	50	33200.09	0.00	*a* ^4^G_4__½_—*y* ${\;}^{4}H_{4½}^{{}^{\circ}}$	
3011.376	70	33197.76	−0.02	*a* ^4^G_3__½_—*y* ${\;}^{4}H_{4½}^{{}^{\circ}}$	6
3012.854	8	33181.48	−0.01	*a* ^6^D_0__½_—*x* ${\;}^{6}F_{0½}^{{}^{\circ}}$	
3013.933	1*h*	33169.60	−0.01	*b* ^4^D_3__½_— $63523_{2½}^{{}^{\circ}}$	6
3014.666	70	33161.54	−0.02	*a* ^4^G_5__½_—*y* ${\;}^{4}H_{5½}^{{}^{\circ}}$	
3015.183	1	33155.85	0.04	*b ^4^*P_2__½_— $66981_{3½}^{{}^{\circ}}$	
3015.915	2	33147.80	$\{\;\begin{array}{c} 0.16\\ 0.2 \end{array}$	*a* ^4^P_1½_—*z* ${\;}^{2}D_{1½}^{{}^{\circ}}$ *a* ^4^D_1__½_—*u* ^6^F°	
3016.454	100	33141.88	0.01	*a* ^4^G_4__½_—*y* ${\;}^{4}H_{5½}^{{}^{\circ}}$	5
3019.018	2	33113.74	−0.05	*a* ^4^P_0½_—*z* ${\;}^{2}D_{1½}^{{}^{\circ}}$	
3021.673	2*h*	33084.64	0.11	*b* ^4^P_2__½_— $66910_{2½}^{{}^{\circ}}$	
3022.743	120	33072.93	0.00	*a* ^4^G_5__½_—*y* ${\;}^{4}H_{6½}^{{}^{\circ}}$	4
3023.993	1*h*	33059.26	−0.04	*a* ^6^D_3½_—*y* ${\;}^{4}F_{4½}^{{}^{\circ}}$	
3027.836	1*h*	33017.30	−0.05	*b* ^4^D_3½_— $63371_{2½}^{{}^{\circ}}$	
3033.324	1*h*	32957.57	0.01	*b* ^4^P_2½_—*u* ${\;}^{4}F_{3½}^{{}^{\circ}}$	
3034.408	1*h*	32945.80	−0.05	*b* ^4^D_1½_— $63371_{2½}^{{}^{\circ}}$	
3038.616	3	32900.17	$\{\;\begin{array}{c} -0.07\\ 0.06 \end{array}$	*b* ^4^D_2½_— $63319_{3½}^{{}^{\circ}}$ *a* ^4^P_2½_—*z* ${\;}^{2}D_{2½}^{{}^{\circ}}$	
3039.169	1	32849.19	0.21	*a* ^4^P_1½_—*w* ${\;}^{4}D_{0½}^{{}^{\circ}}$	
3040.600	100	32878.71	0.02	*a* ^4^G_2__½_—*y* ${\;}^{4}G_{2½}^{{}^{\circ}}$	
3041.220	30	32872.00	0.01	*a* ^4^G_3½_—*y* ${\;}^{4}G_{2½}^{{}^{\circ}}$	P—B
3041.483	1	32869.16	−0.01	*b* ^4^D_2__½_—*x* ${\;}^{2}F_{3½}^{{}^{\circ}}$	
3042.319	3	32860.13	0.00	*a* ^4^P_0½_—*w* ${\;}^{4}D_{0½}^{{}^{\circ}}$	
3042.734	30	32855.65	0.00	*a* ^4^G_2½_—*y* ${\;}^{4}G_{3½}^{{}^{\circ}}$	
3042.905	2	32853.80	0.15	*a* ^4^P_1½_—*z* ${\;}^{2}D_{2½}^{{}^{\circ}}$	
3043.139	15	32851.28	0.02	*a* ^4^G_4½_—*y* ${\;}^{4}G_{3½}^{{}^{\circ}}$	P—B
3043.355	60	32848.95	0.00	*a* ^4^G_3½_—*y* ${\;}^{4}G_{3½}^{{}^{\circ}}$	P—B
3043.768	20	32844.49	−0.01	*a ^4^*G_5½_—*y* ${\;}^{4}G_{4½}^{{}^{\circ}}$	
3044.566	500*r*	32835.88	0.09	*a* ^6^D_4½_—*v* ${\;}^{6}P_{3½}^{{}^{\circ}}$	4
3045.590	80	32824.84	0.03	*a ^4^*G_4½_—*y* ${\;}^{4}G_{4½}^{{}^{\circ}}$	P—B
3045.804	30	32822.53	0.03	*a* ^4^G_3½_—*y* ${\;}^{4}G_{4½}^{{}^{\circ}}$	P—B
3046.588	4	32814.09	−0.05	*a* ^4^F_4½_—*t* ${\;}^{4}G_{5½}^{{}^{\circ}}$	
3047.032	150	32809.31	−0.01	*a* ^4^G_5½_—*y* ${\;}^{4}G_{5½}^{{}^{\circ}}$	6, 7*b*
3048.860	40	32789.64	0.01	*a* ^4^G_4½_—*y* ${\;}^{4}G_{5½}^{{}^{\circ}}$	P—B
3049.967	2*h*	32777.73	−0.07	*a* ^4^F_3½_—*t* ${\;}^{4}G_{4½}^{{}^{\circ}}$	
3050.100	3*h*	32776.31	−0.07	*a* ^4^F_2½_—*t* ^4^G_3½_	
3053.296	2	32742.00	0.23	*a* ^4^P_1½_—*w* ${\;}^{4}D_{1½}^{{}^{\circ}}$	
3054.362	400*r*	32730.57	0.04	*a* ^6^D_3½_—*v* ${\;}^{6}P_{2½}^{{}^{\circ}}$	4
3055.915	2	32713.94	−0.05	*b* ^4^D_1½_—*x* ${\;}^{2}F_{2½}^{{}^{\circ}}$	
3056.471	4	32707.99	0.07	*a* ^4^P_0½_—*w* ${\;}^{4}D_{1½}^{{}^{\circ}}$	
3056.992	4	32702.41	0.04	*b* ^4^D_0½_—*y* ${\;}^{2}D_{1½}^{{}^{\circ}}$	
3057.733	1	32694.49	−0.01	*b* ^4^D_2½_—*y* ${\;}^{2}D_{1½}^{{}^{\circ}}$	
3058.305	2	32688.38	−0.02	*b* ^4^D_1½_—*y* ${\;}^{2}D_{1½}^{{}^{\circ}}$	
3059.217	5H*w*	32678.63	−0.09	*b* ^4^P_2½_— $66504_{1½}^{{}^{\circ}}$	
3062.120	200	32647.65	0.01	*a* ^6^D_2½_—*v* ${\;}^{6}P_{1½}^{{}^{\circ}}$	4
3064.045	2*h*	32627.14	0.00	*a* ^4^D_3½_— $55923_{2½}^{{}^{\circ}}$	
3066.022	250	32606.11	0.03	*a* ^6^D_3½_—*v* ${\;}^{6}P_{3½}^{{}^{\circ}}$	6
3067.858	1	32586.59	−0.06	*z* ${\;}^{6}P_{3½}^{{}^{\circ}}$—*e* ^8^P_4½_	
3070.270	300	32560.99	−0.02	*a* ^6^D_2½_—*v* ${\;}^{6}P_{2½}^{{}^{\circ}}$	6
3073.180	300	32530.69	0.01	*a* ^6^D_1½_—*v* ${\;}^{6}P_{1½}^{{}^{\circ}}$	6
3079.635	200	32461.98	−0.03	*a* ^6^D_0½_—*v* ${\;}^{6}P_{1½}^{{}^{\circ}}$	4
3081.340	100	32444.02	−0.03	*a* ^6^D_1½_—*v* ${\;}^{6}P_{2½}^{{}^{\circ}}$	5
3082.060	40	32436.44	−0.12	*a* ^6^D_2__½_ *v* ${\;}^{6}P_{2½}^{{}^{\circ}}$	5
3082.246	2	32434.48	−0.24	*a* ^4^H_6½_—*u* ${\;}^{4}G_{5½}^{{}^{\circ}}$	
3082.507	2H*w*	32431.74	−0.12	*b* ^4^D_2½_—*w* ${\;}^{6}D_{3½}^{{}^{\circ}}$	
3082.713	15	32429.57	−0.13	*a* ^4^H_6½_—*u* ${\;}^{4}H_{6½}^{{}^{\circ}}$	6, 7*b*
3084.025	1*h*	32415.77	−0.13	*z* ${\;}^{6}P_{3½}^{{}^{\circ}}$—*e* ^8^P_3½_	
3084.879	2*h*	32406.80	−0.32	*b* ^4^D_3½_—*w* ${\;}^{6}D_{2½}^{{}^{\circ}}$?	
3087.378	1	32380.57	0.17	*a* ^4^H_5½_—*v* ${\;}^{2}G_{4½}^{{}^{\circ}}$	
3087.791	3*h*	32376.24	0.35	*b* ^4^D_0½_—*w* ${\;}^{6}D_{1½}^{{}^{\circ}}$?	
3087.944	2*h*	32374.64	0.03	*a* ^4^D_2½_— $55923_{2½}^{{}^{\circ}}$	
3089.686	1H*w*	32356.38	−0.04	*b* ^4^D_0½_—*w* ${\;}^{6}D_{0½}^{{}^{\circ}}$	
3091.098	3*h*	32341.60	−0.12	*b* ^4^D_2½_—*w* ${\;}^{6}D_{2½}^{{}^{\circ}}$	
3091.673	4*h*	32335.59	−0.03	*b* ^4^D_1½_—*w* ${\;}^{6}D_{2½}^{{}^{\circ}}$	
3092.549	7	32326.43	0.08	*a* ^4^P_2½_—*v* ${\;}^{4}D_{1½}^{{}^{\circ}}$	
3092.872	10	32323.05	−0.03	*a* ^4^H_5½_—*u* ${\;}^{4}G_{5½}^{{}^{\circ}}$	
3093.121	2	32320.45	0.16	*a* ^4^P_1½_—*u* ${\;}^{4}P_{0½}^{{}^{\circ}}$	
3093.352	4	32318.04	−0.02	*a* ^4^H_5__½_—*u* ${\;}^{4}H_{6½}^{{}^{\circ}}$	
3093.467	1	32316.84	0.24	*b* ^4^D_3__½_—*w* ${\;}^{6}D_{4½}^{{}^{\circ}}$	
3093.689	2	32314.52	−0.03	*a* ^4^H_3½_—*v* ${\;}^{2}G_{3½}^{{}^{\circ}}$	
3094.818	1	32302.73	0.01	*b* ^4^G_5½_—*v* ${\;}^{2}H_{5½}^{{}^{\circ}}$	
3095.244	1	32298.29	0.01	*z* ${\;}^{6}P_{2½}^{{}^{\circ}}$—*e* ^8^P_2½_	
3096.384	6	32286.39	−0.05	*a* ^4^P_0½_—*u* ${\;}^{4}P_{0½}^{{}^{\circ}}$	
3097.010	20	32279.87	$\{\;\begin{array}{c} -0.02\\ 0.20 \end{array}$	*a* ^4^P_1½_—*v* ${\;}^{4}D_{1½}^{{}^{\circ}}$ *a* ^4^H_6½_—*u* ${\;}^{4}H_{5½}^{{}^{\circ}}$	
3097.063	30	32279.31	0.05	*a* ^4^P_2½_—*v* ${\;}^{4}D_{2½}^{{}^{\circ}}$	
3097.758	5	32272.07	−0.03	*a* ^4^H_5½_—*u* ${\;}^{4}G_{4½}^{{}^{\circ}}$	
3098.092	10	32268.57	−0.03	*a* ^4^P_2½_—*v* ${\;}^{4}D_{3½}^{{}^{\circ}}$	
3099.301	1	32256.01	−0.26	*a* ^4^H_4½_— $66600_{3½}^{{}^{\circ}}$	
3100.267	8	32245.96	−0.08	*a* ^4^P_0½_—*v* ${\;}^{4}D_{1½}^{{}^{\circ}}$	
3100.310	10	322 15.51	0.00	*a* ^4^P_0½_—*v* ${\;}^{4}D_{0½}^{{}^{\circ}}$	
3101.529	30	32232.84	0.04	*a* ^4^P_1½_—*v* ${\;}^{4}D_{2½}^{{}^{\circ}}$	4
3101.836	2	32229.65	−0.05	*a* ^4^H_4½_—*u* ${\;}^{4}G_{3½}^{{}^{\circ}}$	
3103.280	1	32214.65	0.04	*a* ^4^P_2½_—*x* ${\;}^{4}F_{1½}^{{}^{\circ}}$	
3106.336	2	32182.96	0.05	*a* ^4^P_2½_—*u* ${\;}^{4}P_{1½}^{{}^{\circ}}$	
3106.748	10	32178.69	−0.03	*a* ^4^H_4½_—*u* ${\;}^{4}G_{4½}^{{}^{\circ}}$	6
3107.776	20	32168.05	$\{\;\begin{array}{c} -0.10\\ \;0.02 \end{array}$	*a* ^4^P_1½_—*x* ${\;}^{4}F_{1½}^{{}^{\circ}}$ *a* ^4^H_5½_—*u* ${\;}^{4}H_{5½}^{{}^{\circ}}$	6
3108.634	10	32159.17	−0.01	*a* ^4^P_2½_—*x* ${\;}^{4}F_{2½}^{{}^{\circ}}$	6
3109.435	441 *w*	32150.89	−0.19	*b* ^4^D_3½_—*v* ${\;}^{4}F_{3½}^{{}^{\circ}}$	
3110.681	50	32138.01	0.00	*a* ^4^P_2½_—*w* ${\;}^{4}D_{3½}^{{}^{\circ}}$	4
3111.147	2	32133.20	0.05	*b* ^4^D_3__½_—*v* ${\;}^{4}F_{2½}^{{}^{\circ}}$	
3113.118	10	32112.85	0.13	*a* ^4^P_1½_—*x* ${\;}^{4}F_{2½}^{{}^{\circ}}$	4
3113.362	4	32110.33	−0.04	*a* ^4^H_4½_—*u* ${\;}^{4}G_{3½}^{{}^{\circ}}$	5
3113.800	15	32105.81	−0.07	*a* ^4^H_5½_—*u* ${\;}^{4}H_{4½}^{{}^{\circ}}$	5
3114.115	3	32102.57	−0.03	*a* ^4^P_0½_*—u* ${\;}^{4}P_{1½}^{{}^{\circ}}$	
3114.440	1	32099.22	−0.13	*a* ^4^H_3½_—*u* ${\;}^{4}G_{4½}^{{}^{\circ}}$	
3115.462	40	32088.69	0.00	*a* ^4^P_2½_—*x* ${\;}^{4}F_{3½}^{{}^{\circ}}$	4
3115.754	3H *w*	32085.68	0.00	*b* ^4^D_2½_—*v* ${\;}^{4}F_{3½}^{{}^{\circ}}$	
3116.822	2	32074.68	0.03	*a* ^4^H_4½_—*u* ${\;}^{4}H_{5½}^{{}^{\circ}}$	
3117.505	3H *w*	32067.66	−0.09	*b* ^4^D_2½_—*v* ${\;}^{4}F_{2½}^{{}^{\circ}}$	
3118.099	8H *w*	32061.55	−0.10	*b* ^4^D_1½_—*v* ${\;}^{4}F_{2½}^{{}^{\circ}}$	
3120.337	30 *h*	32038.56	−0.05	*b* ^4^D_3½_—*v* ${\;}^{4}F_{4½}^{{}^{\circ}}$	4
3121.072	6	32031.02	0.02	*a* ^4^H_3½_—*u* ${\;}^{4}G_{3½}^{{}^{\circ}}$	6
3121.914	1 *hw*	32022.38	0.3	*Z* ${\;}^{6}P_{1½}^{{}^{\circ}}$—*h* ^8^D	
3122.880	10	32012.47	−0.03	*a* ^4^H_4½_—*u* ${\;}^{4}H_{4½}^{{}^{\circ}}$	6
3125.013	8	31990.62	0.05	*a* ^4^H_4½_—*u* ${\;}^{4}H_{3½}^{{}^{\circ}}$	5
3126.192	2	31978.56	0.10	*b* ^4^D_0½_—*v* ${\;}^{4}F_{1½}^{{}^{\circ}}$	
3126.846	10	31971.87	−0.05	*a* ^4^H_3½_—*u* ${\;}^{4}G_{2½}^{{}^{\circ}}$	5
3129.971	3	31939.95	0.02	*a* ^4^F_3½_— $66981_{3½}^{{}^{\circ}}$	
3130.624	2	31933.29	0.16	*a* ^4^H_3½_—*u* ${\;}^{4}H_{4½}^{{}^{\circ}}$	
3132.284	15 *h*	31916.36	0.06	*a* ^4^F_4½_—*u* ${\;}^{4}F_{4½}^{{}^{\circ}}$	6, 7*b*
3132.789	10	31911.22	0.02	*a* ^4^H_3½_—*u* ${\;}^{4}H_{3½}^{{}^{\circ}}$	6
3134.922	4 *h*	31889.51	0.01	*a* ^4^D_3½_—*x* ${\;}^{4}D_{2½}^{{}^{\circ}}$	
3135.188	2	31886.80	0.06	*z* ${\;}^{6}P_{1½}^{{}^{\circ}}$—*i* ^6^D_0½_	7*b*?
3136.958	10	31868.82	0.22	*a* ^4^P_1½_—*u* ${\;}^{4}P_{2½}^{{}^{\circ}}$	4
3138.222	5	31855.98	0.04	*a* ^4^D_2½_—*w* ${\;}^{4}P_{2½}^{{}^{\circ}}$	
3141.555	1	31822.18	−0.13	*z* ${\;}^{6}P_{1½}^{{}^{\circ}}$—*e* ^4^D_1½_	
3141.821	5	31819.49	0.03	*a* ^4^D_2½_—*w* ${\;}^{4}P_{1½}^{{}^{\circ}}$	
3142.401	1	31813.61	$\{\;\begin{array}{c} -0.02\\ \;0.03 \end{array}$	*a* ^4^F_3½_—*u* ${\;}^{4}F_{4½}^{{}^{\circ}}$ *z* ${\;}^{6}P_{2½}^{{}^{\circ}}$—*e* ^4^D_1½_	
3142.669	20*w*	31810.90	0.05	*a* ^4^D_3½_—*x* ${\;}^{4}D_{3½}^{{}^{\circ}}$	6, 7*b*
3144.119	1	31776.23	−0.04	*a* ^4^F_3½_—*u* ${\;}^{4}F_{2½}^{{}^{\circ}}$	
3144.222	1	31795.19	0.15	*a* ^4^F_2½_— $66910_{2½}^{{}^{\circ}}$	
3144.872	3*h*	31788.62	0.01	*z* ${\;}^{6}P_{1½}^{{}^{\circ}}$—*i* ^6^D_1½_	
3145.458	2*h*	31782.69	0.06	*z* ${\;}^{6}P_{1½}^{{}^{\circ}}$—*e* ^4^D_2½_	
3145, 727	1*h*	31779.98	0.10	*z* ${\;}^{6}P_{2½}^{{}^{\circ}}$—*i* ^6^D_1½_	
3146.324	6*h*	31773.95	0.05	*z* ${\;}^{6}P_{2½}^{{}^{\circ}}$—*e* ^4^D_2½_	
3148.182	200*h*	31755.20	0.03	*z* ${\;}^{8}P_{2½}^{{}^{\circ}}$—*f* ^8^S_3½_	4
3148.857	8	31748.39	−0.04	*a* ^4^H_6½_—*v* ${\;}^{4}G_{5½}^{{}^{\circ}}$	4
3149.529	6*h*	31741.62	−0.06	*a* ^4^F_3½_—*u* ${\;}^{4}F_{3½}^{{}^{\circ}}$	
3149.928	5	31737.60	−0.08	*a* ^4^D_1½_—*w* ${\;}^{4}P_{0½}^{{}^{\circ}}$	4
3150.616	2	31730.67	−0.04	*a* ^4^D_2½_—*x* ${\;}^{4}D_{1½}^{{}^{\circ}}$	
3150.800	1	31728.81	0.00	*a* ^4^F_2½_—*u* ${\;}^{4}F_{1½}^{{}^{\circ}}$	
3151.415	10*h*	31722.62	−0.04	*a* ^4^F_2½_—*u* ${\;}^{4}F_{2½}^{{}^{\circ}}$	
3152.248	5	31714.24	−0.02	*a* ^2^H_5½_—*v* ${\;}^{2}H_{5½}^{{}^{\circ}}$	6, 7*b*
3152.517	4H*w*	31711.53	0.06	*z* ${\;}^{6}P_{1½}^{{}^{\circ}}$—*i* ^6^D_2½_	
3153.393	7H*w*	31702.72	−0.02	*z* ${\;}^{6}P_{2½}^{{}^{\circ}}$—*i* ^6^D_2½_	
3155.094	1	31685.63	0.01	*a* ^4^D_1½_—*w* ${\;}^{4}P_{2½}^{{}^{\circ}}$	
3155.781	10	31678.73	−0.01	*a* ^4^F_1½_—*u* ${\;}^{4}F_{1½}^{{}^{\circ}}$	6, 7*b*
3156.245	2*h*	31674.07	0.04	*z* ${\;}^{6}P_{2½}^{{}^{\circ}}$—*e* ^4^D_3½_	
3156.392	1*h*	31672.60	0.01	*a* ^4^F_1½_—*u* ${\;}^{4}F_{2½}^{{}^{\circ}}$	
3157.658	3*h*	31659.90	0.07	*z* ${\;}^{6}P_{3½}^{{}^{\circ}}$—*e* ^4^D_3½_	
3157.811	3	31658.37	−0.03	*a* ^4^H_5½_—*v* ${\;}^{4}G_{4½}^{{}^{\circ}}$	
3158.723	10	31649.12	−0.02	*a* ^4^D_1½_—*w* ${\;}^{4}P_{1½}^{{}^{\circ}}$	6
3159.825	4	31638.19	−0.14	*a* ^4^D_0½_—*w* ${\;}^{4}P_{0½}^{{}^{\circ}}$	
3159.952	20	31636.92	−0.05	*a* ^4^D_2½_—*x* ${\;}^{4}D_{2½}^{{}^{\circ}}$	6, 7*b*
3160.155	6	31634.89	−0.01	*a* ^4^F_4½_—*u* ${\;}^{4}G_{5½}^{{}^{\circ}}$	4
3161.050	200*h*	31625.93	−0.06	*z* ${\;}^{8}P_{3½}^{{}^{\circ}}$—*f* ^8^S_3½_	6
3162.210	1*h*	31614.33	−0.10	*b* ^4^D_2½_—*y* ${\;}^{2}F_{3½}^{{}^{\circ}}$	
3163.365	1	31602.79	0.01	*a* ^2^H_4½_—*v* ${\;}^{2}H_{5½}^{{}^{\circ}}$	
3163.534	1	31601.10	−0.3	*a* ^4^G_5½_—*u* ^6^F°	
3165.253	1	31583.94	0.02	*a* ^4^F_4½_—*u* ${\;}^{4}G_{4½}^{{}^{\circ}}$	
3166.827	10*h*	31568.24	0.08	*z* ${\;}^{6}P_{2½}^{{}^{\circ}}$—*i* ^6^D_3½_	
3167.153	2	31564.99	−0.03	*a* ^4^H_4½_—*v* ${\;}^{4}G_{4½}^{{}^{\circ}}$	
3167.619	3	31560.35	−0.04	*a* ^4^D_1½_—*x* ${\;}^{4}D_{1½}^{{}^{\circ}}$	
3167.827	8*h*	31558.28	−0.04	*a* ^4^D_2½_—*x* ${\;}^{4}D_{3½}^{{}^{\circ}}$	5
3167.935	1	31557.20	−0.06	*b* ^4^P_1½_—*u* ${\;}^{2}F_{2½}^{{}^{\circ}}$	
3168.254	4*h*	31554.02	0.06	*z* ${\;}^{6}P_{3½}^{{}^{\circ}}$—*i* ^6^D_3½_	
3168.680	2	31549.78	−0.01	*a* ^4^D_0½_—*w* ${\;}^{4}P_{1½}^{{}^{\circ}}$	
3169.356	4	31543.06	0.04	*a* ^2^H_4½_—*v* ${\;}^{2}H_{4½}^{{}^{\circ}}$	6, 7
3170.427	4	31532.40	−0.04	*a* ^4^H_4½_—*v* ${\;}^{2}G_{3½}^{{}^{\circ}}$	
3173.845	3	31498.44	−0.09	*b* ^4^P_1½_—*w* ${\;}^{2}D_{1½}^{{}^{\circ}}$	
3174.746	20H*w*	31489.50	−0.10	*a* ^4^F_1½_— $66654_{2½}^{{}^{\circ}}$	
3175.355	3*h*	31483.47	−0.03	*b* ^4^P_1½_—*w* ${\;}^{2}D_{2½}^{{}^{\circ}}$	
3175.576	12	31481.28	0.03	*a* ^4^F_3½_—*u* ${\;}^{4}G_{4½}^{{}^{\circ}}$	4
3175.713	10	31479.91	0.06	*a* ^4^F_4½_—*u* ${\;}^{4}H_{5½}^{{}^{\circ}}$	
3177.044	10	31466.72	0.07	*a* ^4^D_1½_—*x* ${\;}^{4}D_{2½}^{{}^{\circ}}$	5
3177.616	5	31461.06	0.02	*a* ^4^D_0½_—*x* ${\;}^{4}D_{1½}^{{}^{\circ}}$	5
3178.501	200	31452.30	0.04	*z* ${\;}^{8}P_{4½}^{{}^{\circ}}$—*f* ^8^S_3½_	4
3178.730	2	31450.03	−0.10	*a* ^4^H_3½_—*v* ${\;}^{4}G_{2½}^{{}^{\circ}}$	
3181.269	1	31424.94	0.03	*a* ^4^H_4½_— $65768_{4½}^{{}^{\circ}}$	
3181.990	1	31417.82	0.12	*a* ^4^F_4½_—*u* ${\;}^{4}H_{4½}^{{}^{\circ}}$	
3182.498	2	31412.90	0.00	*a* ^4^F_3½_—*u* ${\;}^{4}G_{3½}^{{}^{\circ}}$	
3185.096	10*h*	31387.18	−0.02	*z* ${\;}^{6}P_{3½}^{{}^{\circ}}$—*i* ^6^D_4½_	
3186.507	1*h*	31373.28	0.03	*b* ^4^D_3½_—*z* ${\;}^{2}F_{2½}^{{}^{\circ}}$	
3187.213	2	31366.33	0.00	*b* ^4^D_2½_—*y* ${\;}^{2}G_{3½}^{{}^{\circ}}$	
3189.959	15	31339.33	0.04	*a* ^4^F_2½_—*u* ${\;}^{4}G_{3½}^{{}^{\circ}}$	4
3192.242	10	31315.11	0.08	*a* ^4^F_3½_—*u* ${\;}^{4}H_{4½}^{{}^{\circ}}$	
3193.788	1	31301.76	0.01	*b* ^4^D_1½_—z ${\;}^{2}F_{2½}^{{}^{\circ}}$	
3194.663	1*h*	31293.19	0.09	*a* ^4^F_3½_—*u* ${\;}^{4}H_{3½}^{{}^{\circ}}$	
3194.855	1*h*	31291.31	−0.06	*b* ^4^D_2½_—*z* ${\;}^{2}F_{3½}^{{}^{\circ}}$	
3195.988	2	31280.22	0.01	*a* ^4^F_2½_—*u* ${\;}^{4}G_{2½}^{{}^{\circ}}$	
3201.113	10	31230.14	0.00	*a* ^4^F_1½_—*u* ${\;}^{4}G_{2½}^{{}^{\circ}}$	5
3202.205	4	31219.49	0.00	*a* ^4^F_2½_—*u* ${\;}^{4}H_{3½}^{{}^{\circ}}$	
3203.131	4	31210.47	0.07	*a* ^4^F_4½_—*u* ${\;}^{2}F_{3½}^{{}^{\circ}}$	
3206.910	80	31173.69	−0.01	*a* ^6^D_4½_—*y* ${\;}^{6}F_{3½}^{{}^{\circ}}$	5
3211.270	4*h*	31131.36	0.23	*b* ^4^D_3½_—*w* ${\;}^{4}G_{4½}^{{}^{\circ}}$	
3212.887	200	31115.70	−0.02	*a* ^6^D_4½_—*y* ${\;}^{6}F_{4½}^{{}^{\circ}}$	6
3216.947	100*r*	31076.42	0.00	*a* ^6^S_2½_—*z* ${\;}^{4}P_{1½}^{{}^{\circ}}$	5
3224.758	150R	31001.15	0.00	*a* ^6^S_2½_—*z* ${\;}^{4}P_{2½}^{{}^{\circ}}$	6
3226.048	100	30988.76	−0.15	*a* ^6^D_3½_—*y* ${\;}^{6}F_{2½}^{{}^{\circ}}$	5
3227.039	2	30979.24	−0.02	*a* ^4^F_3½_—*u* ${\;}^{2}F_{2½}^{{}^{\circ}}$	
3228.092	1000R	30969.14	0.00	*a* ^6^D_4½_—*y* ${\;}^{6}F_{5½}^{{}^{\circ}}$	4
3230.231	10	30948.64	0.03	*a* ^4^F_4½_—*v* ${\;}^{4}G_{5½}^{{}^{\circ}}$	
3230.716	500R	30943.98	−0.01	*a* ^6^D_3½_—*y* ${\;}^{6}F_{3½}^{{}^{\circ}}$	5
3233.939	} 200H	$\left\{ \;\begin{array}{l} 30913.12\\ 30912.67\\ 30911.46 \end{array}\right.$	0.04	*z* ${\;}^{6}P_{1½}^{{}^{\circ}}$—*h* ^6^D_0½_	
3233.997			0.09	*z* ${\;}^{6}P_{1½}^{{}^{\circ}}$—*h* ^6^D_1½_	
3234.112			−0.02	*z* ${\;}^{6}P_{1½}^{{}^{\circ}}$—*h* ^6^D_2½_	
3234.737	4	30905.53	0.03	*a* ^4^F_3½_—*w* ${\;}^{2}D_{2½}^{{}^{\circ}}$	4
3234.912	10*h*	30903.82	−0.03	*z* ${\;}^{6}P_{2½}^{{}^{\circ}}$—*h* ^6^D_1½_	
3235.025	200*h*	30902.74	−0.01	*z* ${\;}^{6}P_{2½}^{{}^{\circ}}$—*h* ^6^D_2½_	P–B
3235.307	300*h*	30900.05	0.00	*z* ${\;}^{6}P_{1½}^{{}^{\circ}}$—*h* ^6^D_3½_	P–B
3236.515	5*h*	30888.55	0.00	*z* ${\;}^{6}P_{3½}^{{}^{\circ}}$—*h* ^6^D_2½_	
3236.778	1000	30886.04	$\{\;\begin{array}{c} 0.03\\ 0.19 \end{array}$	*a* ^6^D_3½_—*y* ${\;}^{6}F_{4½}^{{}^{\circ}}$ (*z* ${\;}^{6}P_{3½}^{{}^{\circ}}$—*h* ^6^D_3½_)	4
3237.019	1	30883.74	0.03	*z* ${\;}^{8}P_{3½}^{{}^{\circ}}$—*f* ^6^S_2½_	
3237.443	500H*w*	30879.67	0.02	*z* ${\;}^{6}P_{3½}^{{}^{\circ}}$—*h* ^6^D_4½_	P–B
3238.720	5	30867.51	−0.04	*a* ^4^F_3½_—*v* ${\;}^{4}G_{4½}^{{}^{\circ}}$	4
3240.408	150	30851.52	0.01	*a* ^6^D_4½_—*y* ${\;}^{6}D_{4½}^{{}^{\circ}}$	6, 7*b*
3240.613	100	30849.49	0.03	*a* ^6^D_2½_—*y* ${\;}^{6}F_{1½}^{{}^{\circ}}$	P–B
3240.882	6	30846.93	0.01	*a* ^4^F_2½_—*w* ${\;}^{2}D_{1½}^{{}^{\circ}}$	
3242.139	1	30834.97	0.00	*a* ^4^F_3½_—*v* ${\;}^{4}G_{3½}^{{}^{\circ}}$	
3242.463	2*h*	30831.89	0.00	*a* ^4^F_2½_—*w* ${\;}^{2}D_{2½}^{{}^{\circ}}$	
3243.777	500	30819.40	0.01	*a* ^6^D_2½_—*y* ${\;}^{6}F_{2½}^{{}^{\circ}}$	6
3246.153	3	30796.84	−0.01	*a* ^4^F_1½_—*w* ${\;}^{2}D_{1½}^{{}^{\circ}}$	
3248.512	700*r*	30774.48	0.01	*a* ^6^D_2½_—*y* ${\;}^{6}F_{3½}^{{}^{\circ}}$	4
3249.894	6	30761.40	0.04	*a* ^4^F_2½_—*v* ${\;}^{4}G_{3½}^{{}^{\circ}}$	P–B
3251.134	150	30749.66	0.02	*a* ^6^D_1½_—*y* ${\;}^{6}F_{0½}^{{}^{\circ}}$	5
3252.949	500*r*	30732.50	0.00	*a* ^6^D_1½_—*y* ${\;}^{6}F_{1½}^{{}^{\circ}}$	6
3254.037	100	30722.23	0.00	*a* ^6^D_4½_—*y* ${\;}^{6}D_{3½}^{{}^{\circ}}$	4
3255.508	5	30708.35	0.00	*a* ^4^F_1½_—*v* ${\;}^{4}G_{2½}^{{}^{\circ}}$	P–B
3256.137	500*r*	30702.42	−0.01	*a* ^6^D_1½_—*y* ${\;}^{6}F_{2½}^{{}^{\circ}}$	4
3258.414	400*r*	30680.96	−0.01	*a* ^6^D_0½_—*y* ${\;}^{6}F_{0½}^{{}^{\circ}}$	6
3260.238	300*r*	30663.80	−0.03	*a* ^6^D_0½_—*y* ${\;}^{6}F_{1½}^{{}^{\circ}}$	4
3262.333	3	30644.10	−0.22	*a* ^4^H_4½_—*w* ${\;}^{2}F_{3½}^{{}^{\circ}}$	
3263.037	2	30637.49	0.01	*a* ^4^H_5½_—*v* ${\;}^{4}H_{4½}^{{}^{\circ}}$	
3264.710	300	30621.79	−0.01	*a* ^6^D_3½_—*y* ${\;}^{6}D_{4½}^{{}^{\circ}}$	4
3267.789	80	30592.94	−0.06	*a* ^4^H_6½_—*v* ${\;}^{4}H_{6½}^{{}^{\circ}}$	6, 7*b*
3268.720	70	30584.23	0.03	*b* ^4^P_2½_—*u* ${\;}^{4}D_{3½}^{{}^{\circ}}$	4
3270.353	60	30568.96	−0.05	*a* ^4^H_5½_—*v* ${\;}^{4}H_{5½}^{{}^{\circ}}$	6, 7*b*
3270.781	5*h*	30564.96	0.01	*a* ^4^H_3½_—*w* ${\;}^{2}F_{3½}^{{}^{\circ}}$	
3273.016	50	30544.09	−0.01	*a* ^4^H_4½_—*v* ${\;}^{4}H_{4½}^{{}^{\circ}}$	6, 7*b*
3278.062	15*h*	30497.07	0.01	*a* ^4^H_3½_—*v* ${\;}^{4}H_{3½}^{{}^{\circ}}$	
3278.551	100	30492.52	0.00	*a* ^6^D_3½_— *y* ${\;}^{6}D_{3½}^{{}^{\circ}}$	6, 7*b*
3279.751	5	30481.37	0.01	*a* ^4^H_5½_—*v* ${\;}^{4}H_{6½}^{{}^{\circ}}$	
3280.370	5	30475.62	−0.01	*a* ^4^H_4½_—*v* ${\;}^{4}H_{5½}^{{}^{\circ}}$	
3280.763	100	30471.96	−0.03	*a* ^6^D_3½_—*y* ${\;}^{6}D_{2½}^{{}^{\circ}}$	
3281.415	1	30465.91	−0.23	*b* ^4^D_3½_—*w* ${\;}^{4}F_{2½}^{{}^{\circ}}$	
3281.532	2	30464.82	0.09	*a* ^4^H_3½_—*v* ${\;}^{4}H_{4½}^{{}^{\circ}}$	
3285.482	1*h*	30428.20	0.02	*a* ^2^I_5½_—*w* ${\;}^{2}H_{5½}^{{}^{\circ}}$	
3288.548	1H	30399.83	−0.11	*a* ^4^H_3½_—*w* ${\;}^{6}F_{2½}^{{}^{\circ}}$	
3288.644	2H	30398.94	0.01	*b* ^4^G_5½_—*t* ${\;}^{4}G_{4½}^{{}^{\circ}}$	
3289.106	1	30394.67	0.03	*b* ^4^D_1½_—*w* ${\;}^{4}F_{2½}^{{}^{\circ}}$	
3290.969	25	30377.46	−0.06	*a* ^6^D_3½_—*w* ${\;}^{6}P_{2½}^{{}^{\circ}}$	4
3294.030	1*h*?	30349.24	0.11	*b* ^4^D_0½_—*w* ${\;}^{4}F_{1½}^{{}^{\circ}}$	
3294.934	1*h*	30340.91	−0.35	*b* ^4^D_2½_—*w* ${\;}^{4}F_{1½}^{{}^{\circ}}$	
3295.840	20	30332.57	−0.03	*b* ^4^G_5½_—*t* ${\;}^{4}G_{5½}^{{}^{\circ}}$	6, 7*b*
3296.025	40	30330.88	−0.03	*a* ^6^D_2½_—*w* ${\;}^{6}P_{1½}^{{}^{\circ}}$	4
3296.879	150	30323.01	0.01	*a* ^6^D_2½_—*y* ${\;}^{6}D_{3½}^{{}^{\circ}}$	4
3298.228	120	30310.61	−0.01	*a* ^4^P_2½_—*y* ${\;}^{4}S_{1½}^{{}^{\circ}}$	4
3300.943	10	30285.68	0.14	*a* ^4^P_2½_—*v* ${\;}^{4}P_{2½}^{{}^{\circ}}$	6, 7*b*
3303.280	100	30264.25	0.09	*a* ^4^P_1½_—*y* ${\;}^{4}S_{1½}^{{}^{\circ}}$	6
3303.681	1*h*	30260.58	−0.16	*b* ^4^G_4½_—*t* ${\;}^{4}G_{3½}^{{}^{\circ}}$	
3304.898	15*h*	30249.44	−0.13	*b* ^4^P_1½_—*u* ${\;}^{4}D_{2½}^{{}^{\circ}}$	
3306.004	2	30239.32	0.24	*a* ^4^P_1½_—*v* ${\;}^{4}P_{2½}^{{}^{\circ}}$	
3306.998	40	30230.23	−0.08	*a* ^4^P_0½_—*y* ${\;}^{4}S_{1½}^{{}^{\circ}}$	4
3308.065	8*h*	30220.48	−0.10	*b* ^4^P_1½_—*u* ${\;}^{4}D_{1½}^{{}^{\circ}}$	6
3308.778	40	30213.97	0.02	*a* ^6^D_1½_—*w* ${\;}^{6}P_{1½}^{{}^{\circ}}$	6
3309.428	2	30208.04	0.04	*a* ^6^D_2½_—*w* ${\;}^{6}P_{2½}^{{}^{\circ}}$	
3311.586	6*hw*	30188.35	−0.20	*b* ^4^G_4½_—*t* ${\;}^{4}G_{4½}^{{}^{\circ}}$	
3311.895	100*l*	30185.54	0.03	*a* ^6^D_1½_—*y* ${\;}^{6}D_{2½}^{{}^{\circ}}$	
3313.050	1H?	30175.01	0.07	*b* ^4^G_2½_—*t* ${\;}^{4}G_{2½}^{{}^{\circ}}$	
3313.200	50*hl*	30173.65	−0.05	*z* ${\;}^{6}P_{1½}^{{}^{\circ}}$—*g* ^6^D_1½_	P–B
3313.458	10*h*	30171.30	−0.04	*z* ${\;}^{6}P_{1½}^{{}^{\circ}}$—*g* ^6^D_2½_	
3313.560	40*h*	30170.36	−0.22	*z* ${\;}^{6}P_{1½}^{{}^{\circ}}$—*g* ^6^D_0½_	P–B
3314.146	10*hl*	30165.03	0.06	*z* ${\;}^{6}P_{2½}^{{}^{\circ}}$—*g* ^6^D_1½_	P–B
3314.415	50*hl*	30162.59	−0.02	*z* ${\;}^{6}P_{2½}^{{}^{\circ}}$—*g* ^6^D_2½_	P–B
3314.889	100*hl*	30158.27	0.23	*z* ${\;}^{6}P_{2½}^{{}^{\circ}}$—*g* ^6^D_3½_	P–B
3315.343	4*h*	30154.14	0.00	*b* ^4^G_3½_—*t* ${\;}^{4}G_{3½}^{{}^{\circ}}$	
3315.975	7*hl*	30148.40	−0.01	*z* ${\;}^{6}P_{3½}^{{}^{\circ}}$—*g* ^6^D_2½_	
3316.319	20	30145.27	−0.01	*a* ^6^D_0½_—*w* ${\;}^{6}P_{1½}^{{}^{\circ}}$	4
3316.459	60*hl*	30144.00	0.16	*z* ${\;}^{6}P_{3½}^{{}^{\circ}}$—*g* ^6^D_3½_	P–B
3317.289	200*hl*	30136.46	0.15	*z* ${\;}^{6}P_{3½}^{{}^{\circ}}$—*g* ^6^D_4½_	P–B
3318.874	1*h*	30122.08	−0.14	*b* ^4^G_4½_—*t* ${\;}^{4}G_{5½}^{{}^{\circ}}$	
3319.873	3	30113.00	0.22	*a* ^4^P_1½_—*v* ${\;}^{4}P_{1½}^{{}^{\circ}}$	
3320.692	100	30105.57	−0.05	*a* ^6^D_3½_—*w* ${\;}^{6}P_{3½}^{{}^{\circ}}$	6
3322.295	6	30091.05	0.01	*a* ^6^D_1½_—*w* ${\;}^{6}P_{2½}^{{}^{\circ}}$	
3323.633	6	30078.93	0.00	*a* ^6^P_0½_—*v* ${\;}^{4}P_{1½}^{{}^{\circ}}$	4
3330.663	100*c*	30015.45	0.31	*a* ^6^D_2½_—*y* ${\;}^{6}D_{1½}^{{}^{\circ}}$	
3334.557	8	29980.40	0.10	*a* ^4^P_1½_—*v* ${\;}^{4}P_{0½}^{{}^{\circ}}$	4
3335.039	3	29976.06	0.03	*b* ^4^D_2½_—*z* ${\;}^{2}D_{1½}^{{}^{\circ}}$	
3338.002	1	29949.46	0.16	*a* ^4^F_4½_—*v* ${\;}^{4}H_{4½}^{{}^{\circ}}$	
3338.288	1*h*	29946.90	0.05	*a* ^4^F_3½_—*w* ${\;}^{2}F_{3½}^{{}^{\circ}}$	
3338.346	2*h*	29946.37	−0.08	*a* ^4^P_0½_—*v* ${\;}^{4}P_{0½}^{{}^{\circ}}$	
3339.126	2*h*	29939.38	−0.03	*b* ^4^P_2½_—*x* ${\;}^{2}D_{2½}^{{}^{\circ}}$	
3339.486	1*h*	29936.15	0.05	*a* ^6^D_2½_—*w* ${\;}^{6}P_{3½}^{{}^{\circ}}$	
3342.065	4	29913.05	0.02	*a* ^4^H_6½_—*x* ${\;}^{2}H_{5½}^{{}^{\circ}}$	
3343.722	60	29898.23	0.05	*a* ^6^D_1½_—*y* ${\;}^{6}D_{1½}^{{}^{\circ}}$	
3345.349	50	29883.68	0.00	*a* ^6^D_1½_—*y* ${\;}^{6}D_{0½}^{{}^{\circ}}$	4
3345.656	2	29880.94	0.11	*a* ^4^F_4½_—*v* ${\;}^{4}H_{5½}^{{}^{\circ}}$	
3349.494	2	29846.71	0.08	*a* ^4^F_3½_—*v* ${\;}^{4}H_{4½}^{{}^{\circ}}$	
3350.409	10*h*	29838.56	−0.13	*b* ^4^P_0½_—*u* ${\;}^{4}D_{1½}^{{}^{\circ}}$	4
3351.423	10	29829.53	0.02	*a* ^6^D_0½_—*y* ${\;}^{6}D_{1½}^{{}^{\circ}}$	
3351.656	20c	29827.46	0.04	*a* ^4^D_3½_—*y* ${\;}^{4}D_{3½}^{{}^{\circ}}$	6, 7*b*
3353.054	1	29815.02	0.01	a ^6^D_0½_—*y* ${\;}^{6}D_{0½}^{{}^{\circ}}$	
3353.328	4*h*	29812.58	0.04	*a* ^4^D_3½_—*y* ${\;}^{4}D_{2½}^{{}^{\circ}}$	
3354.135	1*h*	29805.41	0.06	*a* ^4^F_2½_—*v* ${\;}^{4}H_{3½}^{{}^{\circ}}$	
3354.183	1*h*	29804.98	0.13	*a* ^4^H_5½_—*x* ${\;}^{2}H_{4½}^{{}^{\circ}}$	
3355.479	10	29793.47	0.05	*b* ^4^P_0½_—*u* ${\;}^{4}D_{0½}^{{}^{\circ}}$	6
3356.788	1*h*	29781.86	0.02	*a* ^4^F_3½_—*w* ${\;}^{2}F_{2½}^{{}^{\circ}}$	
3359.419	20H	29758.53	0.18	*b* ^4^P_2½_— $63583_{2½,\;1½}^{{}^{\circ}}$	
3360.681	10	29747.36	−0.08	*b* ^4^D_3½_—*z* ${\;}^{2}D_{2½}^{{}^{\circ}}$	5
3361.054	2	29744.06	−0.08	*a* ^2^H_5½_—*t* ${\;}^{4}G_{5½}^{{}^{\circ}}$	
3362.535	1	29730.96	0.15	*a* ^6^D_1½_—*y* ${\;}^{4}P_{0½}^{{}^{\circ}}$	
3364.188	6	29716.35	0.08	*b* ^4^D_1½_—*w* ${\;}^{4}D_{0½}^{{}^{\circ}}$	
3365.137	8*cw*	29707.97	−0.04	*a* ^4^H_4½_—*x* ${\;}^{2}H_{5½}^{{}^{\circ}}$?	
3365.693	3	29703.06	0.07	*a* ^6^D_2½_—*y* ${\;}^{4}P_{1½}^{{}^{\circ}}$	
3366.235	10	29698.28	−0.05	*b* ^4^P_2½_— $63523_{2½}^{{}^{\circ}}$	6
3368.086	1	29681.96	−0.08	*b* ^4^D_2½_—*z* ${\;}^{2}D_{2½}^{{}^{\circ}}$	
3368.194	7*h*	29681.00	0.02	*z* ${\;}^{6}P_{1½}^{{}^{\circ}}$—*h* ^6^S_2½_	
3369.188	10*h*	29672.25	0.00	*z* ${\;}^{6}P_{2½}^{{}^{\circ}}$—*h* ^6^S_2½_	
3370.813	15*h*	29657.95	−0.10	*z* ${\;}^{6}P_{3½}^{{}^{\circ}}$—*h* ^6^S_2½_	
3372.087	15	29646.74	0.00	*a* ^4^F_4½_—*x* ${\;}^{2}G_{4½}^{{}^{\circ}}$	6
3375.223	3	29619.20	0.07	*a* ^6^D_3½_—*y* ${\;}^{4}P_{2½}^{{}^{\circ}}$	
3376.527	6	29607.76	−0.07	*a* ^4^F_3½_—*x* ${\;}^{2}G_{3½}^{{}^{\circ}}$	
3378.871	3	29587.22	0.02	*a* ^4^D_3½_—*x* ${\;}^{6}D_{2½}^{{}^{\circ}}$	
3380.283	5*c*	29574.86	−0.03	*a* ^4^D_2_*_½_—y* ${\;}^{4}D_{3½}^{{}^{\circ}}$	
3380.444	10*c*	29573.46	0.03	*a* ^4^D_3½_—*x* ${\;}^{6}D_{3½}^{{}^{\circ}}$	
3380.817	10	29570.19	0.03	*b* ^4^D_2½_—*w* ${\;}^{4}D_{1½}^{{}^{\circ}}$	
3381.981	4*c*	29560.01	0.00	*a* ^4^D_2½_—*y* ${\;}^{4}D_{2½}^{{}^{\circ}}$	
3382.673	3	29553.96	−0.03	*a* ^4^D_2½_—*y* ${\;}^{4}D_{1½}^{{}^{\circ}}$	
3383.585	9*h*	29546.00	−0.07	*b* ^4^P_2½_— $63371_{2½}^{{}^{\circ}}$	
3386.863	1	29517.40	0.04	*a* ^6^D_0½_—*y* ${\;}^{4}P_{1½}^{{}^{\circ}}$	
3389.287	2	29496.29	0.09	*a* ^2^H_5½_—*w* ${\;}^{2}H_{5½}^{{}^{\circ}}$	6, 7*b*
3393.843	1	29456.70	0.04	*a* ^2^H_4½_—*w* ${\;}^{2}H_{4½}^{{}^{\circ}}$	6, 7*b*
3396.367	2*h*	29434.81	0.05	*b* ^4^G_5½_—*u* ${\;}^{4}F_{4½}^{{}^{\circ}}$	
3399.878	1	29404.41	0.08	*a* ^2^I_6½_—*u* ${\;}^{4}H_{6½}^{{}^{\circ}}$	
3401.578	3	29389.72	0.03	*a* ^4^D_1½_—*y* ${\;}^{4}D_{2½}^{{}^{\circ}}$	
3402.490	2	29381.84	$\left\{ \;\begin{array}{r} 0.04\\ -0.11 \end{array}\right.$	*a* ^4^D_1½_—*y* ${\;}^{4}D_{0½}^{{}^{\circ}}$ *b* ^4^P_1½_—*x* ${\;}^{2}D_{1½}^{{}^{\circ}}$	
3406.122	1	29350.51	−0.17	*b* ^4^G_4½_— $66981_{3½}^{{}^{\circ}}$	
3407.962	20	29334.66	−0.01	*a* ^4^D_2½_—*x* ${\;}^{6}D_{2½,\;1½}^{{}^{\circ}}$	4
3408.188	1	29332.72	0.07	*a* ^6^D_1½_—*y* ${\;}^{4}P_{2½}^{{}^{\circ}}$	
3409.561	3*c*	29320.91	0.01	*a* ^4^D_2½_—*x* ${\;}^{6}D_{3½}^{{}^{\circ}}$	
3410.345	1	29314.17	−0.04	*b* ^4^P_2½_—*x* ${\;}^{2}F_{2½}^{{}^{\circ}}$	
3410.800	5	29310.26	0.01	*a* ^4^H_6½_—*y* ${\;}^{2}H_{5½}^{{}^{\circ}}$	
3411.816	1	29301.53	0.00	*b* ^4^P_1½_—*x* ${\;}^{2}D_{2½}^{{}^{\circ}}$	
3412.543	3*hl*	29295.29	0.02	*a* ^2^F_3½_—*t* ${\;}^{4}G_{2½}^{{}^{\circ}}$	
3413.815	1	29284.37	0.05	*a* ^4^D_0½_—*y* ${\;}^{4}D_{1½}^{{}^{\circ}}$	
3417.158	6	29255.73	−0.06	*b* ^4^P_2½_—*y* ${\;}^{2}D_{2½}^{{}^{\circ}}$	
3418.276	15	29246.15	0.01	*b* ^4^D_3½_—*w* ${\;}^{4}D_{2½}^{{}^{\circ}}$	5
3420.794	50	29224.63	−0.03	*a* ^4^H_6½_—*w* ${\;}^{4}H_{6½}^{{}^{\circ}}$	6, 7*b*
3421.491	1	29218.67	0.06	*b* ^4^G_2½_— $67008_{2½}^{{}^{\circ}}$	
3422.418	1	29210.76	0.08	*b* ^4^G_5½_—*v* ${\;}^{2}G_{4½}^{{}^{\circ}}$	
3422.826	10	29207.28	−0.05	*a* ^4^H_5½_—*w* ${\;}^{4}H_{5½}^{{}^{\circ}}$	6, 7*b*
3423.137	2	29204.63	0.04	*a* ^4^H^4^_½_—*z* ${\;}^{2}H_{4½}^{{}^{\circ}}$	6
3423.841	10	29198.62	0.01	*a* ^4^H_5½_—*y* ${\;}^{2}H_{5½}^{{}^{\circ}}$	
3424.375	5	29194.07	−0.02	*a* ^4^H_5½_—*w* ${\;}^{4}H_{4½}^{{}^{\circ}}$	
3425.929	1	29180.82	0.08	*b* ^4^D_2½_—*w* ${\;}^{4}D_{2½}^{{}^{\circ}}$	
3427.865	10	29164.34	−0.01	*a* ^4^D_1½_—*x* ${\;}^{6}D_{2½,\;1½}^{{}^{\circ}}$	
3427.957	3	29163.56	−0.02	*a* ^4^D_1½_—*x* ${\;}^{6}D_{0½}^{{}^{\circ}}$	
3428.782	15	29156.55	0.00	*b* ^4^D_0½_—*u* ${\;}^{4}P_{0½}^{{}^{\circ}}$	6
3429.155	4	29153.37	0.01	*b* ^4^G_5½_—*u* ${\;}^{4}G_{5½}^{{}^{\circ}}$	
3429.741	2	29148.39	0.05	*b* ^4^G_5½_—*u* ${\;}^{4}H_{6½}^{{}^{\circ}}$	
3430.428	1	29142.56	−0.02	*b* ^4^D_1½_—*u* ${\;}^{4}P_{0½}^{{}^{\circ}}$	
3433.025	5*h*	29120.51	0.04	*b* ^4^P_1½_— $63583_{2½,\;1½}^{{}^{\circ}}$	
3433.572	50	29115.87	−0.06	*b* ^4^D_3½_—*v* ${\;}^{4}D_{3½}^{{}^{\circ}}$	6
3433.911	1	29113.00	−0.02	*a* ^4^H_5½_—*w* ${\;}^{4}H_{6½}^{{}^{\circ}}$	
3434.473	2	29108.23	−0.05	*b* ^4^D_2½_—*v* ${\;}^{4}D_{1½}^{{}^{\circ}}$	
3434.823	1	29105.27	0.04	*a* ^4^H_4½_—*y* ${\;}^{2}H_{5½}^{{}^{\circ}}$	
3435.193	5	29102.13	−0.05	*b* ^4^D_1½_—*v* ${\;}^{4}D_{1½}^{{}^{\circ}}$	P–B
3435.254	4	29101.62	−0.03	*b* ^4^D_1½_—*v* ${\;}^{4}D_{0½}^{{}^{\circ}}$	P–B
3435.375	5	29100.59	$\left\{ \;\begin{array}{r} -0.12\\ 0.04 \end{array}\right.$	*a* ^4^H_4½_—*w* ^4^ ${\;}^{4}H_{4½}^{{}^{\circ}}$ *a* ^4^H_3½_— $63523_{2½}^{{}^{\circ}}$	6, 7*b*
3439.579	3	29065.02	0.02	*a* ^4^D_0½_—*x* ${\;}^{6}D_{1½}^{{}^{\circ}}$	
3439.673	3	29064.23	0.00	*a* ^4^D_0½_—*x* ${\;}^{6}D_{0½}^{{}^{\circ}}$	
3440.044	10	29061.10	−0.09	*b* ^4^D_2½_—*v* ${\;}^{4}D_{2½}^{{}^{\circ}}$	6
3440.769	1	29054.97	−0.12	*b* ^4^D_1½_—*v* ${\;}^{4}D_{2½}^{{}^{\circ}}$	
3440.907	1	29053.79	−0.07	*b* ^4^G_2½_—*u* ${\;}^{4}F_{1½}^{{}^{\circ}}$	
3441.177	2	29051.53	−0.02	*a* ^4^H_4½_—*w* ${\;}^{4}H_{3½}^{{}^{\circ}}$	
3442.756	5	29038.20	−0.06	*a* ^4^H_5½_—*z* ${\;}^{2}H_{5½}^{{}^{\circ}}$	6, 7*b*
3443.657	5*h*	29030.60	−0.03	*a* ^4^H_4½_— $63374_{4½}^{{}^{\circ}}$	
3444.237	2*hc*	29025.72	−0.26	*b* ^4^P_2½_—*w* ${\;}^{6}D_{3½}^{{}^{\circ}}$	
3446.519	1	29006.50	−0.01	*b* ^4^D_3½_—*x* ${\;}^{4}F_{2½}^{{}^{\circ}}$	
3446.766	2	29004.42	0.01	*b* ^4^D_0½_—*x* ${\;}^{4}F_{1½}^{{}^{\circ}}$	
3446.822	5	29003.95	−0.06	*a* ^4^H_4½_—*y* ${\;}^{2}H_{4½}^{{}^{\circ}}$	6
3448.429	1*h*	28990.44	0.00	*b* ^4^D_1½_—*x* ${\;}^{4}F_{1½}^{{}^{\circ}}$	
3449.036	1	28985.34	0.00	*b* ^4^D_3½_—*w* ${\;}^{4}D_{3½}^{{}^{\circ}}$	
3450.607	20	28972.14	−0.04	*a* ^4^H_3½_—*w* ${\;}^{4}H_{3½}^{{}^{\circ}}$	6
3450.932	1*h*	28969.41	−0.14	*b* ^4^G_4½_— $66600_{3½}^{{}^{\circ}}$	
3451.475	7	28964.85	0.01	*b* ^4^D_2½_—*u* ${\;}^{4}P_{1½}^{{}^{\circ}}$	4
3452.201	5	28958.76	0.02	*b* ^4^D_1½_—*u* ${\;}^{4}P_{1½}^{{}^{\circ}}$	
3452.440	5*w*	28956.75	0.18	*a* ^4^D_3½_—*u* ${\;}^{6}P_{3½}^{{}^{\circ}}$	
3453.426	2*h*	28948.49	0.01	*a* ^6^D_4½_—*y* ${\;}^{8}P_{3½}^{{}^{\circ}}$	
3453.505	2*hw*	28947.83	−0.06	*b* ^4^G_2½_—*v* ${\;}^{2}G_{3½}^{{}^{\circ}}$	
3453.861	1	28944.84	$\left\{ \;\begin{array}{l} -0.04\\ -0.04 \end{array}\right.$	*a* ^4^H_4½_—*z* ${\;}^{2}H_{5½}^{{}^{\circ}}$ *a* ^4^H_4½_—*x* ${\;}^{2}F_{3½}^{{}^{\circ}}$	
3454.110	2	28942.76	−0.22	*b* ^4^G_4½_—*u* ${\;}^{4}G_{5½}^{{}^{\circ}}$	
3454.309	1	28941.09	−0.02	*b* ^4^D_2½_—*x* ${\;}^{4}F_{2½}^{{}^{\circ}}$	
3454.926	8	28935.92	$\left\{ \;\begin{array}{r} 0.08\\ -0.10 \end{array}\right.$	*b* ^4^P_2½_—*w* ${\;}^{6}D_{2½}^{{}^{\circ}}$ *b* ^4^D_3½_—*x* ${\;}^{4}F_{3½}^{{}^{\circ}}$	
3455.045	8	28934.92	−0.09	*b* ^4^D_1½_—*x* ${\;}^{4}F_{2½}^{{}^{\circ}}$	
3456.268	1	28924.69	0.05	*a* ^4^H_3½_—*y* ${\;}^{2}H_{4½}^{{}^{\circ}}$	
3456.850	1	28919.81	−0.13	*b* ^4^D_2½_—*w* ${\;}^{4}D_{3½}^{{}^{\circ}}$	
3458.246	2*h*	28908.14	−0.05	*b* ^4^P_1½_— $63371_{2½}^{{}^{\circ}}$	
3458.842	5	28903.16	−0.07	*b* ^4^D_3½_—*x* ${\;}^{4}F_{4½}^{{}^{\circ}}$	
3462.755	10	28870.50	−0.12	*b* ^4^D_2½_—*x* ${\;}^{4}F_{3½}^{{}^{\circ}}$	P–B
3463.356	3	28865.49	−0.02	*a* ^4^H_3½_—*x* ${\;}^{2}F_{3½}^{{}^{\circ}}$	
3463.657	15	28862.98	0.03	*b* ^4^G_3½_— $66600_{3½}^{{}^{\circ}}$	6
3470.013	15	28810.12	−0.12	*b* ^4^G_2½_— $66600_{3½}^{{}^{\circ}}$	7*b*?
3472.986	1	28785.45	0.05	*b* ^4^G_3½_—*u* ${\;}^{4}G_{4½}^{{}^{\circ}}$	
3475.776	20	28762.35	−0.04	*b* ^4^D_3½_—*u* ${\;}^{4}P_{2½}^{{}^{\circ}}$	4
3478.645	2*hl*	28738.63	$\left\{ \;\begin{array}{r} 0.05\\ -0.02 \end{array}\right.$	*b* ^4^P_0½_— $63583_{1½}^{{}^{\circ}}$ *a* ^2^I_5½_—*v* ${\;}^{4}G_{5½}^{{}^{\circ}}$	
3480.609	5*h*	28722.41	0.14	*a* ^4^P_2½_— $55923_{2½}^{{}^{\circ}}$	
3481.053	1	28718.75	−0.02	*a* ^6^D_3½_—*y* ${\;}^{8}P_{3½}^{{}^{\circ}}$	
3481.342	2	28716.36	−0.07	*a* ^4^H_3½_—*x* ${\;}^{2}F_{2½}^{{}^{\circ}}$	
3483.084	20	28702.00	0.02	*a* ^6^D_4½_—*z* ${\;}^{4}D_{3½}^{{}^{\circ}}$	
3483.689	6	28697.02	0.03	*b* ^4^D_2½_—*u* ${\;}^{4}P_{2½}^{{}^{\circ}}$	
3484.355	2	28691.54	0.06	*a* ^2^G_4½_—*v* ${\;}^{2}H_{5½}^{{}^{\circ}}$	
3485.724	2*h*	28680.27	0.15	*z* ${\;}^{8}P_{3½}^{{}^{\circ}}$—*e* ^6^D_3½_	
3486.239	2*h*	28676.03	0.22	*a* ^4^P_1½_— $55923_{2½}^{{}^{\circ}}$	
3487.658	1	28664.36	0.02	*b* ^4^G_2½_—*u* ${\;}^{4}G_{3½}^{{}^{\circ}}$	
3488.309	5	28659.01	0.08	*a* ^6^D_3½_—*z* ${\;}^{4}D_{2½}^{{}^{\circ}}$	
3491.548	1	28632.43	0.06	*a* ^6^D_2½_—*z* ${\;}^{4}D_{1½}^{{}^{\circ}}$	
3492.786	1	28622.28	0.06	*a* ^2^H_5½_—*v* ${\;}^{2}G_{4½}^{{}^{\circ}}$	
3493.352	1	28617.64	0.00	*a* ^2^H_4½_—*v* ${\;}^{2}G_{3½}^{{}^{\circ}}$	
3494.861	2*h*	28605.29	0.03	*b* ^4^G_2½_—*u* ${\;}^{4}G_{2½}^{{}^{\circ}}$	
3503.727	2	28532.91	0.13	*a* ^6^D_0½_—*z* ${\;}^{4}D_{0½}^{{}^{\circ}}$	
3504.085	2*h*	28529.99	0.07	*a* ^6^D_2½_—*y* ${\;}^{8}P_{2½}^{{}^{\circ}}$	
3505.408	1	28519.22	0.07	*a* ^4^F_4½_—*w* ${\;}^{4}H_{5½}^{{}^{\circ}}$	
3505.863	2	28515.52	0.11	*a* ^6^D_1½_—*z* ${\;}^{4}D_{1½}^{{}^{\circ}}$	
3507.158	2*h*	28504.99	0.06	*a* ^4^F_3½_— $63546_{3½}^{{}^{\circ}}$	
3507.538	3*h*	28501.90	−0.01	*z* ${\;}^{8}P_{4½}^{{}^{\circ}}$—*e* ${\;}^{6}D_{4½}^{{}^{\circ}}$	
3509.067	3	28489.48	0.07	*a* ^6^D_2½_—*z* ${\;}^{4}D_{2½}^{{}^{\circ}}$	
3509.168	1	28488.66	−0.02	*b* ^4^G_5½_—*v* ${\;}^{4}G_{4½}^{{}^{\circ}}$	
3511.188	4	28472.28	0.01	*a* ^6^D_3½_—*z* ${\;}^{4}D_{3½}^{{}^{\circ}}$	
3511.831	20	28467.06	−0.01	*b* ^4^G_5½_—*v* ${\;}^{4}G_{5½}^{{}^{\circ}}$	6, 7
3514.330	1	28446.82	0.08	*a* ^6^D_0½_—*z* ${\;}^{4}D_{1½}^{{}^{\circ}}$	
3515.690	3*h*	28435.82	−0.01	*a* ^4^F_4½_— $63374_{4½}^{{}^{\circ}}$	
3516.053	2	28432.88	−0.02	*a* ^2^G_3½_—*v* ${\;}^{2}H_{4½}^{{}^{\circ}}$	
3518.989	2*h*	28409.16	$\left\{ \;\begin{array}{r} -0.05\\ 0.03 \end{array}\right.$	*a* ^4^F_4½_—*y* ${\;}^{2}H_{4½}^{{}^{\circ}}$ *a* ^4^D_1½_—*u* ${\;}^{6}P_{2½}^{{}^{\circ}}$	
3523.530	3	28372.54	0.09	*a* ^6^D_1½_—*z* ${\;}^{4}D_{2½}^{{}^{\circ}}$	
3525.212	1*h*	28359.01	−0.16	*b* ^4^G_2½_—*u* ${\;}^{2}F_{3½}^{{}^{\circ}}$	
3526.322	1	28350.09	$\{\;\begin{array}{c} 0.01\\ 0.01 \end{array}$	*a* ^4^F_4½_—*z* ${\;}^{2}H_{5½}^{{}^{\circ}}$ *a* ^4^F_4½_—*x* ${\;}^{2}F_{3½}^{{}^{\circ}}$	
3526.492	1	28348.72	0.15	*b* ^4^G_5½_— $65768_{4½}^{{}^{\circ}}$	
3529.534	1	28324.29	0.03	*b* ^4^P_1½_—*w* ${\;}^{6}D_{1½}^{{}^{\circ}}$	
3531.836	1600	28305.82	−0.05	*z* ${\;}^{8}P_{2½}^{{}^{\circ}}$—*e* ^8^D_3½_	P–B
3531.998	1800	28304.53	−0.04	*z* ${\;}^{8}P_{2½}^{{}^{\circ}}$—*e* ^8^D_2½_	P–B
3532.110	2000	28303.63	0.00	*z* ${\;}^{8}P_{2½}^{{}^{\circ}}$—*e* ^8^D_1½_	P–B
3535.297	10	28278.11	−0.19	*b* ^4^G_4½_—*v* ${\;}^{4}G_{4½}^{{}^{\circ}}$	6, 7*b*
3538.002	5	28256.50	−0.19	*b* ^4^G_4½_—*v* ${\;}^{4}G_{5½}^{{}^{\circ}}$	
3539.371	2	28245.57	−0.15	*b* ^4^G_4½_—*v* ${\;}^{4}G_{3½}^{{}^{\circ}}$	
3541.234	1	28230.70	0.00	*b* ^4^G_2½_—*u* ${\;}^{2}F_{2½}^{{}^{\circ}}$	
3547.794	4000	28178.51	0.00	*z* ${\;}^{8}P_{3½}^{{}^{\circ}}$—*e* ^8^D_4½_	P–B
3548.022	3000	28176.69	0.00	*z* ${\;}^{8}P_{3½}^{{}^{\circ}}$—*e* ^8^D_3½_	P–B
3548.182	1000	28175.43	0.04	*z* ${\;}^{8}P_{3½}^{{}^{\circ}}$—*e* ^8^D_2½_	P–B
3552.757	10	28139.14	0.02	*b* ^4^G_3½_—*v* ${\;}^{4}G_{3½}^{{}^{\circ}}$	
3552.901	4	28138.00	−0.19	*b* ^4^G_4½_— $65768_{4½}^{{}^{\circ}}$	
3553.121	1	28136.26	0.08	*b* ^4^G_3½_—*v* ${\;}^{4}G_{2½}^{{}^{\circ}}$	
3555.066	2	28120.87	0.21	*a* ^4^P_1½_—*w* ${\;}^{4}P_{1½}^{{}^{\circ}}$	
3559.373	2	28086.84	0.03	*a* ^4^P_0½_—*w* ${\;}^{4}P_{1½}^{{}^{\circ}}$	
3559.434	2	28086.36	−0.05	*b* ^4^G_2½_—*v* ${\;}^{4}G_{3½}^{{}^{\circ}}$	
3559.808	10	28083.41	−0.06	*b* ^4^G_2½_—*v* ${\;}^{4}G_{2½}^{{}^{\circ}}$	
3561.736	1	28068.21	−0.01	*a* ^2^F_3½_—*v* ${\;}^{2}G_{3½}^{{}^{\circ}}$	
3566.330	1	28032.05	0.14	*a* ^4^P_1½_—*x* ${\;}^{4}D_{1½}^{{}^{\circ}}$	
3566.731	3	28028.91	−0.01	*a* ^2^H_4½_—*u* ${\;}^{2}F_{3½}^{{}^{\circ}}$	
3569.494	6000	28007.21	0.00	*z* ${\;}^{8}P_{4½}^{{}^{\circ}}$—*e* ^8^D_5½_	P–B
3569.804	3000	28004.78	0.00	*z* ${\;}^{8}P_{4½}^{{}^{\circ}}$—*e* ^8^D_4½_	P–B
3570.028	1000	28003.02	0.06	*z* ${\;}^{8}P_{4½}^{{}^{\circ}}$—*e* ^8^D_3½_	P–B
3572.354	1	27984.79	0.16	*a* ^4^P_2½_—*x* ${\;}^{4}D_{2½}^{{}^{\circ}}$	
3575.356	1	27961.29	−0.03	*a* ^2^F_3½_—*v* ${\;}^{2}G_{4½}^{{}^{\circ}}$	
3575.967	4H	27956.51	0.00	*z* ${\;}^{6}P_{1½}^{{}^{\circ}}$—*f* ^6^D_0½_	
3576.073	8H	27955.68	−0.01	*z* ${\;}^{6}P_{1½}^{{}^{\circ}}$—*f* ^6^D_1½_	
3576.303	4H	27953.89	−0.01	*z* ${\;}^{6}P_{1½}^{{}^{\circ}}$—*f* ^6^D_2½_	
3577.187	2H	27946.98	0.02	*z* ${\;}^{6}P_{2½}^{{}^{\circ}}$—*f* ^6^D_1½_	
3577.416	5H	27945.19	0.02	*z* ${\;}^{6}P_{2½}^{{}^{\circ}}$—*f* ^6^D_2½_	
3577.870	2000	27941.64	0.01	*a* ^6^D_4½_—*x* ${\;}^{6}P_{3½}^{{}^{\circ}}$	4
3578.285	3	27938.40	0.23	*a* ^4^P_1½_—*x* ${\;}^{4}D_{2½}^{{}^{\circ}}$	
3579.637	8	27927.85	−0.31	*z* ${\;}^{6}P_{3½}^{{}^{\circ}}$—*f* ^6^D_3½_	
3580.112	40H*l*	27924.14	0.00	*Z* ${\;}^{6}P_{3½}^{{}^{\circ}}$—*f* ^6^D_4½_	
3581.545	5H*w*	27912.97	0.20	*a* ^4^F_4½_—*w* ${\;}^{6}D_{3½}^{{}^{\circ}}$	
3582.425	20*c*	27906.11	0.13	*a* ^4^P_2½_—*x* ${\;}^{4}D_{3½}^{{}^{\circ}}$	4
3583.187	2	27900.18	−0.04	*a* ^2^H_5½_—*v* ${\;}^{4}G_{4½}^{{}^{\circ}}$	
3583.675	40	27896.38	−0.07	*a* ^4^D_2½_—*x* ${\;}^{4}P_{1½}^{{}^{\circ}}$	4
3585.966	1	27878.56	−0.05	*a* ^2^H_5½_—*v* ${\;}^{4}G_{5½}^{{}^{\circ}}$	
3586.540	1000*h*	27874.10	−0.01	*a* ^6^D_3½_—*x* ${\;}^{6}P_{2½}^{{}^{\circ}}$	4
3590.664	2	27842.09	0.05	*b* ^4^G_5½_—*w* ${\;}^{2}G_{4½}^{{}^{\circ}}$	
3591.806	15	27833.23	−0.03	*a* ^4^D_1½_—*x* ${\;}^{4}P_{0½}^{{}^{\circ}}$	4
3592.556	1	27827.42	−0.02	*b* ^4^G_2½_—*v* ${\;}^{2}F_{3½}^{{}^{\circ}}$	
3595.110	500*h*	27807.65	0.00	*a* ^6^D_2½_—*x* ${\;}^{6}P_{1½}^{{}^{\circ}}$	4
3597.560	1	27788.72	−0.02	*a* ^2^H_4½_—*v* ${\;}^{4}G_{4½}^{{}^{\circ}}$	
3599.504	4H*w*	27773.71	0.02	*a* ^4^H_6½_—*y* ${\;}^{2}I_{6½}^{{}^{\circ}}$	
3601.268	20	27760.11	0.00	*a* ^2^H_5½_— $65768_{4½}^{{}^{\circ}}$	4
3601.772	20*c*	27756.22	0.01	*a* ^4^D_2½_—*x* ${\;}^{4}P_{2½}^{{}^{\circ}}$	6
3604.670	15*c*	27733.91	0.00	*a* ^4^D_0½_—*x* ${\;}^{4}P_{0½}^{{}^{\circ}}$	6
3605.683	20	27726.12	−0.01	*a* ^4^D_1½_—*x* ${\;}^{4}P_{1½}^{{}^{\circ}}$	6
3606.489	2H	27719.92	−0.04	*a* ^4^F_3½_—*w* ${\;}^{6}D_{2½}^{{}^{\circ}}$	
3606.702	1H	27718.28	0.00	*a* ^4^D_3½_—*x* ${\;}^{6}F_{3½}^{{}^{\circ}}$	
3607.530	1000	27711.92	0.00	*a* ^6^D_3½_—*x* ${\;}^{6}P_{3½}^{{}^{\circ}}$	6
3608.485	1000	27704.59	0.00	*a* ^6^D_2½_—*x* ${\;}^{6}P_{2½}^{{}^{\circ}}$	6
3610.298	1000	27690.68	−0.01	*a* ^6^D_1½_—*x* ${\;}^{6}P_{1½}^{{}^{\circ}}$	6
3612.612	2H	27672.94	0.29	*a* ^4^F_2½_—*w* ${\;}^{6}D_{1½}^{{}^{\circ}}$	
3615.375	10H	27651.78	0.03	*a* ^4^H_3½_—*y* ${\;}^{2}F_{2½}^{{}^{\circ}}$	5
3618.646	2	27626.80	0.02	*a* ^4^D_0½_—*x* ${\;}^{4}P_{1½}^{{}^{\circ}}$	
3619.272	600	27622.02	0.00	*a* ^6^D_0½_—*x* ${\;}^{6}P_{1½}^{{}^{\circ}}$	4
3620.751	1*h*	27610.73	−0.04	*a* ^4^H_3½_—*y* ${\;}^{2}F_{3½}^{{}^{\circ}}$	
3621.483	6H*w*	27605.15	−0.01	*a* ^4^H_6½_— $61744_{5½}^{{}^{\circ}}$	
3623.783	500*h*	27587.63	0.00	*a* ^6^D_1½_—*x* ${\;}^{6}P_{2½}^{{}^{\circ}}$	5
3626.299	3H*w*	27568.49	−0.06	*a* ^4^H_5½_—*y* ${\;}^{2}I_{5½}^{{}^{\circ}}$	
3629.738	400*h*	27542.38	−0.02	*a* ^6^D_2½_—*x* ${\;}^{6}P_{3½}^{{}^{\circ}}$	5
3635.699	10	27497.22	0.03	*a* ^2^H_4½_—*v* ${\;}^{2}F_{3½}^{{}^{\circ}}$	4
3636.190	2H*w*	27493.51	−0.01	*a* ^4^H_5½_— $61744_{5½}^{{}^{\circ}}$	
3638.032	1*h*	27479.59	0.09	*a* ^2^F_3½_—*u* ${\;}^{2}F_{3½}^{{}^{\circ}}$	
3639.145	5	27471.18	0.02	*a* ^6^D_4½_—*z* ${\;}^{4}F_{3½}^{{}^{\circ}}$	
3639.580	1	27467.90	0.14	*b* ^4^G_5½_—*v* ${\;}^{4}H_{4½}^{{}^{\circ}}$	
3640.086	15*h*	27464.08	$\{\;\begin{array}{c} 0.16\\ 0.08 \end{array}$	*a* ^4^F_3½_—*v* ${\;}^{4}F_{3½}^{{}^{\circ}}$ *a* ^4^H_5½_—*y* ${\;}^{2}G_{4½}^{{}^{\circ}}$	
3641.405	30*h*	27454.13	0.01	*a* ^4^F_4½_—*v* ${\;}^{4}F_{4½}^{{}^{\circ}}$	6, 7*b*
3643.018	3	27441.98	−0.06	*a* ^4^H_4½_—*y* ${\;}^{2}G_{3½}^{{}^{\circ}}$	5
3646.707	3	27414.22	−0.07	*a* ^6^D_3½_—*z* ${\;}^{4}F_{2½}^{{}^{\circ}}$	
3648.698	6	27399.26	−0.03	*b* ^4^G_5½_—*v* ${\;}^{4}H_{5½}^{{}^{\circ}}$	6
3652.292	3H	27372.30	−0.08	*a* ^4^F_2½_—*v* ${\;}^{4}F_{2½}^{{}^{\circ}}$	
3652.510	1*h*	27370.66	0.04	*a* ^4^H_4½_—*y* ${\;}^{2}G_{4½}^{{}^{\circ}}$	
3653.514	2	27363.14	−0.07	*a* ^6^D_2½_—*z* ${\;}^{4}F_{1½}^{{}^{\circ}}$	
3653.581	2	27362.64	−0.03	*a* ^4^H_3½_—*y* ${\;}^{2}G_{3½}^{{}^{\circ}}$	
3654.291	2*h*	27357.32	−0.28	*b* ^4^G_4½_—*w* ${\;}^{2}F_{3½}^{{}^{\circ}}$	
3655.097	3*h*	27351.29	$\left\{ \;\begin{array}{r} -0.16\\ 0.26 \end{array}\right.$	*a* ^4^F_3½_—*v* ${\;}^{4}F_{4½}^{{}^{\circ}}$ *a* ^2^F_3½_—*u* ${\;}^{2}F_{2½}^{{}^{\circ}}$	
3657.906	20*c*	27330.29	−0.04	*a* ^4^H_6½_—*w* ${\;}^{4}G_{5½}^{{}^{\circ}}$	5
3660.404	100	27311.64	0.00	*b* ^4^G_5½_—*v* ${\;}^{4}H_{6½}^{{}^{\circ}}$	4
3663.135	1*h*	27291.27	0.02	*a* ^4^H_3½_—*y* ${\;}^{2}G_{4½}^{{}^{\circ}}$	
3663.373	2*h*	27289.50	−0.21	*b* ^4^G_4½_—*v* ${\;}^{4}H_{3½}^{{}^{\circ}}$	
2667.714	10	27257.21	−0.17	*b* ^4^G_4½_—*v* ${\;}^{4}H_{4½}^{{}^{\circ}}$	6
3668.201	1	27253.59	0.01	*a* ^2^H_5½_—*w* ${\;}^{2}G_{4½}^{{}^{\circ}}$	
3668.547	1*h*	27251.02	0.02	*b* ^4^G_3½_—*w* ${\;}^{2}F_{3½}^{{}^{\circ}}$	
3669.198	4	27246.18	−0.07	*a* ^6^D_1½_—*z* ${\;}^{4}F_{1½}^{{}^{\circ}}$	
3669.398	15	27244.70	−0.07	*a* ^6^D_2½_—*z* ${\;}^{4}F_{2½}^{{}^{\circ}}$	
3669.837	50	27241.44	−0.01	*a* ^6^D_3½_—*z* ${\;}^{4}F_{3½}^{{}^{\circ}}$	
3670.505	100	27236.48	0.01	*a* ^6^D_4½_—*z* ${\;}^{4}F_{4½}^{{}^{\circ}}$	6
3672.037	1	27225.12	−0.03	*a* ^4^F_1½_—*v* ${\;}^{4}F_{1½}^{{}^{\circ}}$	
3672.915	1	27218.61	0.08	*a* ^4^H_5½_—*w* ${\;}^{4}G_{5½}^{{}^{\circ}}$	
3673.515	2	27214.16	0.00	*a* ^2^F_2½_—*u* ${\;}^{2}F_{3½}^{{}^{\circ}}$	
3674.514	1	27206.76	0.02	*a* ^2^F_3½_—*v* ${\;}^{4}G_{3½}^{{}^{\circ}}$	
3675.670	10H*l*	27198.21	−0.08	*b* ^4^G_2½_—*w* ${\;}^{2}F_{3½}^{{}^{\circ}}$	
3676.960	80	27188.67	−0.24	*b* ^4^G_4½_—*v* ${\;}^{4}H_{5½}^{{}^{\circ}}$	4
3677.469	1	27184.90	−0.05	*a* ^2^H_4½_—*w* ${\;}^{2}G_{3½}^{{}^{\circ}}$	
3677.716	4*h*	27183.08	−0.03	*b* ^4^G_3½_—*v* ${\;}^{4}H_{3½}^{{}^{\circ}}$	
3678.470	3	27177.50	−0.08	*a* ^6^D_0½_—*z* ${\;}^{4}F_{1½}^{{}^{\circ}}$	
3680.146	20	27165.12	−0.08	*b* ^4^G_5½_—*x* ${\;}^{2}G_{4½}^{{}^{\circ}}$	4
3682.090	60	27150.79	0.01	*b* ^4^G_3½_—*v* ${\;}^{4}H_{4½}^{{}^{\circ}}$	4
2683.363	4	27141.40	−0.04	*a* ^4^H_4½_—*w* ${\;}^{4}G_{4½}^{{}^{\circ}}$	
3684.522	20	27132.87	0.00	*b* ^4^D_3½_—*v* ${\;}^{4}P_{2½}^{{}^{\circ}}$	4
3684.866	20*h*	27130.33	−0.07	*b* ^4^G_2½_—*v* ${\;}^{4}H_{3½}^{{}^{\circ}}$	
3685.215	15	27127.76	−0.05	*a* ^6^D_1½_—*z* ${\;}^{4}F_{2½}^{{}^{\circ}}$	
3685.561	7	27125.22	−0.09	*a* ^4^H_4½_—*w* ${\;}^{4}G_{5½}^{{}^{\circ}}$	
3689.097	2	27099.22	$\{\;\begin{array}{c} 0.01\\ 0.10 \end{array}$	*a* ^2^F_3½_— $65768_{4½}^{{}^{\circ}}$ *a* ^4^D_1½_—*x* ${\;}^{6}F_{0½}^{{}^{\circ}}$	4
3689.989	1*h*	27092.67	0.12	*b* ^4^D_2½_—*y* ${\;}^{4}S_{1½}^{{}^{\circ}}$	
3690.808	10	27086.66	−0.01	*a* ^4^H_6½_—*y* ${\;}^{4}I_{6½}^{{}^{\circ}}$	
3690.933	5*h*	27085.74	$\left\{ \;\begin{array}{r} 0.05\\ -0.25 \end{array}\right.$	*a* ^2^F_2½_—*u* ${\;}^{2}F_{2½}^{{}^{\circ}}$ *b* ^4^G_3½_—*w* ${\;}^{2}F_{2½}^{{}^{\circ}}$	
3692.187	2*h*	27076.54	−0.02	*a* ^4^D_3½_—*y* ${\;}^{4}F_{2½}^{{}^{\circ}}$	
3692.817	40	27071.92	−0.01	*a* ^6^D_2½_—*z* ${\;}^{4}F_{3½}^{{}^{\circ}}$	4
3693.426	4	27067.46	−0.01	*b* ^4^D_2½_—*v* ${\;}^{4}P_{2½}^{{}^{\circ}}$	
3693.671	100	27065.66	0.00	*a* ^4^H_6½_—*y* ${\;}^{4}I_{7½}^{{}^{\circ}}$	4
$\begin{array}{l} 3694.056\\ 3694.116 \end{array}\}$	20*c*	$\left\{ \;\begin{array}{l} 27062.84\\ 24062.40 \end{array}\right.$	$\begin{array}{r} 0.23\\ -0.21 \end{array}\}$	*a* ^4^D_3½_—*y* ${\;}^{4}F_{3½}^{{}^{\circ}}$	$\left\{ \;\begin{array}{r} 6\\ P–B \end{array}\right.$
3696.093	2*h*	27047.93	−0.03	*a* ^4^H_3½_—*w* ${\;}^{4}G_{2½}^{{}^{\circ}}$	
3696.547	100 *cw*	27044.61	−0.02	*a* ^4^D_3½_—*y* ${\;}^{4}F_{4½}^{{}^{\circ}}$	4
3700.147	8	27018.29	−0.29	*b* ^4^G_4½_—*x* ${\;}^{2}G_{3½}^{{}^{\circ}}$	
3700.300	15	27017.18	0.01	*a* ^4^P_2½_—*z* ${\;}^{4}S_{1½}^{{}^{\circ}}$	4
3701.728	80	27006.75	−0.01	*a* ^6^D_3½_—*z* ${\;}^{4}F_{4½}^{{}^{\circ}}$	4
3706.082	150	26975.03	0.00	*a* ^4^H_5½_—*y* ${\;}^{4}I_{6½}^{{}^{\circ}}$	
3706.663	10	26970.80	0.09	*a* ^4^P_1½_—*z* ${\;}^{4}S_{1½}^{{}^{\circ}}$	6, 7*b*
3708.148	1*h*	26960.00	−0.04	*a* ^4^F_2½_—*y* ${\;}^{2}F_{2½}^{{}^{\circ}}$	
3708.870	3	26954.75	−0.07	*b* ^4^G_4½_—*x* ${\;}^{2}G_{4½}^{{}^{\circ}}$	
3709.656	1	26949.04	0.00	*b* ^4^D_0½_—*v* ${\;}^{4}P_{1½}^{{}^{\circ}}$	
3709.831	3	26947.77	0.00	*a* ^2^F_3½_—*v* ${\;}^{2}F_{3½}^{{}^{\circ}}$	6
3710.749	8	26941.10	−0.07	*b* ^4^D_2½_—*v* ${\;}^{2}P_{1½}^{{}^{\circ}}$	4
3711.349	4	26936.75	−0.11	*a* ^4^P_0½_—*z* ${\;}^{4}S_{1½}^{{}^{\circ}}$	4
3711.590	6	26935.00	−0.07	*b* ^4^D_1½_—*v* ${\;}^{4}P_{1½}^{{}^{\circ}}$	
3713.789	6*h*	26919.05	−0.01	*a* ^4^F_2½_—*y* ${\;}^{2}F_{3½}^{{}^{\circ}}$	4
3714.771	1	26911.93	−0.05	*b* ^4^G_3½_—*x* ${\;}^{2}G_{3½}^{{}^{\circ}}$	
3715.046	5*h*	26909.94	−0.03	*a* ^4^F_1½_—*y* ${\;}^{2}F_{2½}^{{}^{\circ}}$	5
3715.525	4	26906.47	−0.24	*a* ^2^I_5½_—*x* ${\;}^{2}H_{4½}^{{}^{\circ}}$	
3718.129	4	26887.63	−0.03	*a* ^2^I_6½_—*x* ${\;}^{2}H_{5½}^{{}^{\circ}}$	
3718.924	100	26881.88	0.01	*a* ^4^H_4½_—*y* ${\;}^{4}I_{5½}^{{}^{\circ}}$	
3720.908	5	26867.55	0.02	*a* ^4^H_4½_—*y* ${\;}^{4}I_{4½}^{{}^{\circ}}$	6
3722.062	1	26859.22	−0.05	*b* ^4^G_2½_—*x* ${\;}^{2}G_{3½}^{{}^{\circ}}$	
3725.548	2	26834.08	0.01	*a* ^4^D_2½_—*y* ${\;}^{4}F_{1½}^{{}^{\circ}}$	
3726.949	20	26824.00	−0.03	*a* ^4^D_2½_—*y* ${\;}^{4}F_{2½}^{{}^{\circ}}$	6
3727.986	4	26816.54	−0.02	*b* ^4^D_0½_—*v* ${\;}^{4}P_{0½}^{{}^{\circ}}$	6
3728.894	100*c*	26810.01	−0.07	*a* ^4^D_2½_—*y* ${\;}^{4}F_{3½}^{{}^{\circ}}$	4
3729.523	2H*l*	26805.48	0.14	*a* ^4^F_4½_— $61744_{5½}^{{}^{\circ}}$	
3729.942	3	26802.47	−0.12	*b* ^4^D_1½_—*v* ${\;}^{4}P_{0½}^{{}^{\circ}}$	
3731.012	10	26794.79	−0.06	*a* ^4^H_6½_—*x* ${\;}^{4}H_{5½}^{{}^{\circ}}$	
3731.938	100	26788.14	−0.02	*a* ^4^H_3½_—*y* ${\;}^{4}I_{4½}^{{}^{\circ}}$	5
3733.643	1*h*	26775.91	$\left\{ \;\begin{array}{r} 0.09\\ -0.01 \end{array}\right.$	*a* ^4^F_4½_—*y* ${\;}^{2}G_{4½}^{{}^{\circ}}$ *a* ^4^D_3½_—*z* ${\;}^{4}H_{4½}^{{}^{\circ}}$	
3736.909	50	26752.53	−0.07	*a* ^4^H_6½_—*x* ${\;}^{4}H_{6½}^{{}^{\circ}}$	6, 7*b*
3738.014	1*h*	26744.60	0.03	*a* ^4^F_3½_—*y* ${\;}^{2}G_{3½}^{{}^{\circ}}$	
3741.019	10	26723.11	−0.07	*a* ^2^H_5½_—*v* ${\;}^{4}H_{6½}^{{}^{\circ}}$	
3742.269	1H	26714.19	0.00	*a* ^2^F_2½_—*v* ${\;}^{2}F_{2½}^{{}^{\circ}}$	
3743.525	10	26705.23	−0.13	*a* ^4^H_5½_—*x* ${\;}^{4}H_{4½}^{{}^{\circ}}$	
3744.362	2	26699.26	−0.09	*a* ^2^H_4½_—*v* ${\;}^{4}H_{5½}^{{}^{\circ}}$	
3746.623	40	26683.15	−0.06	*a* ^4^H_5½_—*X* ${\;}^{4}H_{5½}^{{}^{\circ}}$	6, 7*b*
3748.021	8*h*	26673.19	0.04	*a* ^4^F_3½_—*y* ${\;}^{2}G_{4½}^{{}^{\circ}}$	
3748.332	1	26670.98	0.02	*a* ^4^F_2½_—*y* ${\;}^{2}G_{3½}^{{}^{\circ}}$	
3748.515	7*hl*	26669.68	$\{\;\begin{array}{c} 0.07\\ 0.01 \end{array}$	*a* ^4^F_3½_—*z* ${\;}^{2}F_{3½}^{{}^{\circ}}$ *b* ^2^I_6½_—*v* ${\;}^{2}H_{5½}^{{}^{\circ}}$	
3749.353	10	26663.71	−0.04	*a* ^4^D_1½_—*y* ${\;}^{4}F_{1½}^{{}^{\circ}}$	6
3750.766	60	26653.67	−0.04	*a* ^4^D_1½_—*y* ${\;}^{4}F_{2½}^{{}^{\circ}}$	4
3752.554	5	26640.97	0.01	*a* ^4^H_5½_—*x* ${\;}^{4}H_{6½}^{{}^{\circ}}$	
3753.303	2*h*	26635.66	0.13	*a* ^2^F_3½_—*w* ${\;}^{2}G_{3½}^{{}^{\circ}}$	
3753.866	8	26631.66	−0.01	*b* ^4^G_5½_—*x* ${\;}^{2}H_{5½}^{{}^{\circ}}$	
3754.224	5H*w*	26629.12	−0.29	*z* ${\;}^{4}P_{1½}^{{}^{\circ}}$—*f* ^4^D_0½_	
3755.385	1H*w*	26620.89	0.14	*z* ${\;}^{4}P_{2½}^{{}^{\circ}}$—*f* ^4^D_1½_	
3756.449	5	26613.35	0.04	*a* ^4^H_4½_—*x* ${\;}^{4}H_{3½}^{{}^{\circ}}$	
3756.643	30	26611.98	0.00	*a* ^4^H_4½_—*x* ${\;}^{4}H_{4½}^{{}^{\circ}}$	
3758.912	4*h*	26595.91	−0.09	*a* ^4^F_2½_—*z* ${\;}^{2}F_{3½}^{{}^{\circ}}$	
3759.354	2*h*	26592.79	0.11	*a* ^2^F_3½_—*w* ${\;}^{2}G_{4½}^{{}^{\circ}}$	
3759.775	6	26589.81	−0.02	*a* ^4^H_4½_—*x* ${\;}^{4}H_{5½}^{{}^{\circ}}$	
3761.010	5H*w*	26581.08	0.20	*z* ${\;}^{4}P_{0½}^{{}^{\circ}}$—*f* ${\;}^{4}D_{0½}^{{}^{\circ}}$	
3761.629	1	26576.70	−0.04	*a* ^2^H_5½_—*x* ${\;}^{2}G_{4½}^{{}^{\circ}}$	
3763.372	40	26564.40	0.00	*a* ^4^D_0½_—*y* ${\;}^{4}F_{1½}^{{}^{\circ}}$	5
3763.650	2*h*	26562.43	0.02	*a* ^4^F_1½_—*z* ${\;}^{2}F_{2½}^{{}^{\circ}}$	
3766.052	10H	26545.49	0.01	*z* ${\;}^{4}P_{1½}^{{}^{\circ}}$—*f* ^4^D_1½_	
3767.695	20	26533.92	−0.02	*a* ^4^H_3½_—*x* ${\;}^{4}H_{3½}^{{}^{\circ}}$	
3767.876	2	26532.64	0.03	*a* ^4^H_3½_—*x* ${\;}^{4}H_{4½}^{{}^{\circ}}$	
3768.176	10	26530.53	0.02	*a* ^4^F_4½_—*w* ${\;}^{4}G_{5½}^{{}^{\circ}}$	
3770.226	1H?	26516.11	−0.15	*a* ^4^D_2½_—*z* ${\;}^{4}H_{3½}^{{}^{\circ}}$	
3771.439	15	26507.58	0.00	*b* ^2^I_6½_—*x* ${\;}^{2}I_{6½}^{{}^{\circ}}$	6, 7*b*
3772.955	10H	26496.93	−0.02	*z* ${\;}^{4}P_{0½}^{{}^{\circ}}$—*f* ^4^D_1½_	
3773.858	10	26490.58	0.00	*b* ^2^I_5½_—*x* ${\;}^{2}I_{5½}^{{}^{\circ}}$	6, 7*b*
3774.669	20*h*	26484.89	0.07	*z* ${\;}^{4}P_{2½}^{{}^{\circ}}$—*f* ^4^D_2½_	
3776.289	2*h*	26473.53	0.11	*a* ^2^G_4½_—*w* ${\;}^{2}H_{5½}^{{}^{\circ}}$	
3776.537	40	26471.79	0.00	*a* ^6^D_4½_—*z* ${\;}^{6}F_{3½}^{{}^{\circ}}$	
3781.192	1*h*	26439.20	−0.03	*a* ^4^F_3½_—*w* ${\;}^{4}G_{3½}^{{}^{\circ}}$	
3783.297	2	26424.49	−0.26	*b* ^4^G_4½_—*x* ${\;}^{2}H_{4½}^{{}^{\circ}}$	
3784.233	1	26417.96	−0.01	*a* ^4^H_5½_—*z* ${\;}^{2}G_{4½}^{{}^{\circ}}$	
3785.422	30*h*	26409.66	0.11	*z* ${\;}^{4}P_{1½}^{{}^{\circ}}$—*f* ^4^D_2½_	4
3786.836	3	26399.80	−0.03	*a* ^2^I_5½_—*z* ${\;}^{2}H_{4½}^{{}^{\circ}}$	7*b*
3787.446	1	26395.55	0.03	*a* ^4^H_4½_—*z* ${\;}^{2}G_{3½}^{{}^{\circ}}$	
3789.757	1*h*	26379.45	−0.19	*a* ^4^D_1½_—*v* ${\;}^{6}P_{1½}^{{}^{\circ}}$	
3790.214	200*h*	26376.27	−0.02	*a* ^6^D_4½_—*z* ${\;}^{6}F_{4½}^{{}^{\circ}}$	6
3791.081	2*h*	26370.22	0.03	*a* ^2^F_2½_—*w* ${\;}^{2}G_{3½}^{{}^{\circ}}$	
3794.497	2	26346.50	−0.04	*a* ^2^G_3½_—*w* ${\;}^{2}H_{4½}^{{}^{\circ}}$	
3799.256	60	26313.50	0.00	*a* ^6^D_3½_—*z* ${\;}^{6}F_{2½}^{{}^{\circ}}$	5
3800.551	60*h*	26304.53	0.06	*z* ${\;}^{4}P_{2½}^{{}^{\circ}}$—*f* ^4^D_3½_	4
3801.901	80	26295.19	0.00	*a* ^4^G_5½_—*z* ${\;}^{4}G_{5½}^{{}^{\circ}}$	6, 7*b*
3802.137	1	26293.56	−0.04	*a* ^2^I_6½_—*w* ${\;}^{4}H_{5½}^{{}^{\circ}}$	
3803.073	2	26287.09	0.02	*a* ^4^F_4½_—*y* ${\;}^{4}I_{5½}^{{}^{\circ}}$	
3804.021	6	26280.54	0.01	*a* ^4^G_5½_—*z* ${\;}^{4}G_{4½}^{{}^{\circ}}$	
3804.752	20*c*	26275.49	−0.01	*a* ^4^G_4½_—*z* ${\;}^{4}G_{5½}^{{}^{\circ}}$	
3806.715	2000*h*	26261.94	0.00	*a* ^6^D_4½_—*z* ${\;}^{6}F_{5½}^{{}^{\circ}}$	4
3806.881	10	26260.80	−0.04	*a* ^4^G_4½_—*z* ${\;}^{4}G_{4½}^{{}^{\circ}}$	
3807.203	10	26258.58	0.05	*a* ^4^G_3½_—*z* ${\;}^{4}G_{4½}^{{}^{\circ}}$	
3808.506	10	26249.59	0.02	*a* ^4^G_2½_—*z* ${\;}^{4}G_{3½}^{{}^{\circ}}$	
3809.146	5	26245.18	0.00	*a* ^4^G_4½_—*z* ${\;}^{4}G_{3½}^{{}^{\circ}}$	
3809.485	10	26242.85	−0.02	*a* ^4^G_3½_—*Z* ${\;}^{4}G_{3½}^{{}^{\circ}}$	
3809.593	500*h*	26242.10	0.02	*a* ^6^D_3½_—*z* ${\;}^{6}F_{3½}^{{}^{\circ}}$	6
3810.679	40	26234.62	0.03	*a* ^4^G_2½_—*z* ${\;}^{4}G_{2½}^{{}^{\circ}}$	
3811.659	6	26227.88	−0.01	*a* ^4^G_3½_—*z* ${\;}^{4}G_{2½}^{{}^{\circ}}$	
3813.024	1	26218.49	0.09	*a* ^2^F_3½_—*_V_* ${\;}^{4}H_{4½}^{{}^{\circ}}$	
3816.746	100	26192.92	−0.01	*a* ^6^D_2½_—*z* ${\;}^{6}F_{1½}^{{}^{\circ}}$	5, C
3820.081	1*h*	26170.06	0.00	*a* ^4^F_3½_—*y* ${\;}^{4}I_{4½}^{{}^{\circ}}$	
3820.903	1*h*	26164.42	0.14	*b* ^4^P_2½_—*w* ${\;}^{4}D_{1½}^{{}^{\circ}}$	
3823.508	1500*h*	26146.60	0.02	*a* ^6^D_3½_—*z* ${\;}^{6}F_{4½}^{{}^{\circ}}$	4
3823.891	100*h*	26143.98	0.00	*a* ^6^D_2½_—*z* ${\;}^{6}F_{2½}^{{}^{\circ}}$	6, C
3826.617	5*h*	26125.36	0.00	*z* ${\;}^{6}P_{1½}^{{}^{\circ}}$—*g* ^6^S_2½_	
3826.734	1*h*	26124.55	0.02	*a* ^2^I_6½_—*z* ${\;}^{2}H_{5½}^{{}^{\circ}}$	
3827.890	10H	26116.67	0.04	*z* ${\;}^{6}P_{2½}^{{}^{\circ}}$—*g* ^6^S_2½_	
3829.679	100	26104.47	0.29	*a* ^6^D_1½_—*z* ${\;}^{6}F_{0½}^{{}^{\circ}}$	5, C
3829.986	8*h*	26102.38	−0.05	*z* ${\;}^{6}P_{3½}^{{}^{\circ}}$—*g* ^6^S_2½_	
3833.865	500	26075.97	0.00	*a* ^6^D_1½_—*z* ${\;}^{6}F_{1½}^{{}^{\circ}}$	6, C
3834.368	1000	26072.55	−0.01	*a* ^6^D_2½_—*z* ${\;}^{6}F_{3½}^{{}^{\circ}}$	4
3836.908	1*h*	26055.29	−0.10	*b* ^4^G_2½_—*x* ${\;}^{2}D_{1½}^{{}^{\circ}}$	
3838.191	2*hl*	26046.58	−0.09	*a* ^2^H_5½_—*x* ${\;}^{2}H_{4½}^{{}^{\circ}}$	
3839.779	500*h*	26035.81	0.30	*a* ^6^D_0½_—*z* ${\;}^{6}F_{0½}^{{}^{\circ}}$	6, C
3841.074	600*h*	26027.03	0.01	*a* ^6^D_1½_—*z* ${\;}^{6}F_{2½}^{{}^{\circ}}$	4, C
3843.988	500*h*	26007.30	0.00	*a* ^6^D_0½_—*z* ${\;}^{6}F_{1½}^{{}^{\circ}}$	4, C
3845.009	7*h*	26000.40	0.13	*a* ^4^F_4½_—*w* ${\;}^{4}F_{4½}^{{}^{\circ}}$	
3845.795	1	25995.08	0.05	*a* ^4^F_4½_—*x* ${\;}^{4}H_{5½}^{{}^{\circ}}$	
3847.234	1*h*	25985.36	−0.03	*a* ^2^F_2½_—*v* ${\;}^{4}H_{3½}^{{}^{\circ}}$	
3846.046	1	25979.88	0.28	*a* ^2^F_3½_—*x* ${\;}^{2}G_{3½}^{{}^{\circ}}$	
3850.380	1*h*	25964.12	0.02	*a* ^4^F_4½_—*w* ${\;}^{4}F_{3½}^{{}^{\circ}}$	
3852.512	6*h*	25949.76	−0.06	*a* ^2^G_4½_— $66981_{3½}^{{}^{\circ}}$	
3853.476	30	25943.27	−0.03	*b* ^4^G_5½_—*w* ${\;}^{4}H_{6½}^{{}^{\circ}}$	4
3854.674	1	25935.17	−0.02	*a* ^2^H_4½_—*x* ${\;}^{2}H_{4½}^{{}^{\circ}}$	
3855.114	4*h*	25932.24	−0.03	*b* ^4^P_1½_—*z* ${\;}^{2}D_{1½}^{{}^{\circ}}$	
3856.539	50	25922.66	0.11	*a* ^4^P_2½_—*y* ${\;}^{4}D_{3½}^{{}^{\circ}}$	4
3857.280	1	25917.68	−0.19	*b* ^4^G_4½_—*z* ${\;}^{2}H_{4½}^{{}^{\circ}}$	
3857.563	1*h*	25915.78	$\left\{ \;\begin{array}{l} -0.06\\ -0.06 \end{array}\right.$	*a* ^4^F_3½_—*x* ${\;}^{4}H_{3½}^{{}^{\circ}}$ *a* ^2^F_3½_—*x* ${\;}^{2}G_{4½}^{{}^{\circ}}$	
3857.753	1	25914.51	0.00	*a* ^4^F_3½_—*x* ${\;}^{4}H_{4½}^{{}^{\circ}}$	
3858.749	5	25907.82	0.15	*a* ^4^P_2½_—*y* ${\;}^{4}D_{2½}^{{}^{\circ}}$	
3859.397	1	25903.47	0.03	*a* ^4^G_5½_—*x* ${\;}^{6}F_{5½}^{{}^{\circ}}$	
3860.258	3*h*	25897.69	0.09	*a* ^4^F_3½_—*w* ${\;}^{4}F_{4½}^{{}^{\circ}}$	
3862.320	1*h*	25883.86	0.11	*a* ^4^G_4½_—*x* ${\;}^{6}F_{5½}^{{}^{\circ}}$	
3865.672	20	25861.42	0.21	*a* ^4^P_1½_—*y* ${\;}^{4}D_{2½}^{{}^{\circ}}$	
3866.574	2	25855.39	0.20	*a* ^4^P_1½_—*y* ${\;}^{4}D_{1½}^{{}^{\circ}}$	
3868.535	1	25842.28	0.05	*a* ^2^F_2½_—*x* ${\;}^{4}H_{3½}^{{}^{\circ}}$	
3870.822	4	25827.01	−0.22	*b* ^4^G_4½_—*w* ${\;}^{4}H_{5½}^{{}^{\circ}}$	
3871.345	1H	25823.52	0.00	*a* ^2^G_4½_—*u* ${\;}^{4}F_{4½}^{{}^{\circ}}$	
3871.671	6*h*	25821.34	0.00	*a* ^4^P_0½_—*y* ${\;}^{4}D_{1½}^{{}^{\circ}}$	
2871.953	2	25819.47	0.00	*a* ^4^P_0½_—*y* ${\;}^{4}D_{0½}^{{}^{\circ}}$	
3872.127	10	25818.31	−0.20	*b* ^4^G_4½_—*y* ${\;}^{2}H_{5½}^{{}^{\circ}}$	
3873.200	10	25811.16	−0.11	*b* ^4^G_3½_—*z* ${\;}^{2}H_{4½}^{{}^{\circ}}$	
3874.743	1*h*	25800.87	0.15	*a* ^4^F_4½_—*z* ${\;}^{2}G_{3½}^{{}^{\circ}}$?	
3876.712	3	25787.77	−0.05	*a* ^4^F_2½_—*w* ${\;}^{4}F_{3½}^{{}^{\circ}}$	
3878.155	5*h*	25778.18	−0.06	*a* ^2^G_3½_— $67008_{2½}^{{}^{\circ}}$	
3883.249	20	25744.36	0.10	*b* ^2^I_6½_—*z* ${\;}^{2}K_{7½}^{{}^{\circ}}$	4
3887.380	2	25717.00	−0.29	*b* ^4^G_4½_—*y* ${\;}^{2}H_{4½}^{{}^{\circ}}$	
3888.841	7	25707.35	−0.04	*b* ^4^G_3½_—*w* ${\;}^{4}H_{4½}^{{}^{\circ}}$	
3889.461	20	25703.25	0.00	*b* ^2^I_5½_—*z* ${\;}^{2}K_{6½}^{{}^{\circ}}$	5
3891.624	5	25688.96	−0 04	*a* ^4^H_6½_—*z* ${\;}^{2}I_{5½}^{{}^{\circ}}$	
3892.621	15	25682.38	0.05	*a* ^4^P_2½_—*x* ${\;}^{6}D_{2½,1½}^{{}^{\circ}}$	
3893.199	1	25678.57	−0.04	*b* ^4^P_1½_—*w* ${\;}^{4}D_{0½}^{{}^{\circ}}$	
3894.714	60	25668.58	0.02	*a* ^4^P_2½_—*x* ${\;}^{6}D_{3½}^{{}^{\circ}}$	4
3896.339	20	25657.87	$\left\{ \;\begin{array}{l} -0.29\\ -0.29 \end{array}\right.$	*b* ^4^G_4½_—*z* ${\;}^{2}H_{5½}^{{}^{\circ}}$ *b* ^4^G_4½_—*x* ${\;}^{2}F_{3½}^{{}^{\circ}}$	4
3896.743	2*h*	25655.21	$\left\{ \;\begin{array}{l} -0.10\\ -0.09 \end{array}\right.$	*b* ^4^P_2½_—*v* ${\;}^{4}D_{2½}^{{}^{\circ}}$ *a* ^4^F_1½_—*w* ${\;}^{4}F_{2½}^{{}^{\circ}}$	
3898.368	50*c*	25644.52	−0.13	*b* ^4^P_2½_—*v* ${\;}^{4}D_{3½}^{{}^{\circ}}$	4
3899.336	10	25638.15	−0.13	*b* ^4^P_1½_—*z* ${\;}^{2}D_{2½}^{{}^{\circ}}$	4
3899.620	30	25636.29	0.42	*a* ^4^P_1½_—*x* ${\;}^{6}D_{2½,\;1½}^{{}^{\circ}}$	
3903.525	10	25610.64	−0.05	*b* ^4^G_3½_—*y* ${\;}^{2}H_{4½}^{{}^{\circ}}$	
3904.328	20	25605.37	−0.15	*b* ^4^G_2½_—*w* ${\;}^{4}H_{3½}^{{}^{\circ}}$	5
3904.852	15	25601.94	−0.08	*a* ^4^P_0½_—*x* ${\;}^{6}D_{1½}^{{}^{\circ}}$	P–B
3904.967	15	25601.18	−0.07	*a* ^4^P_0½_—*x* ${\;}^{6}D_{0½}^{{}^{\circ}}$	P–B
3905.237	5*h*	25599.42	−0.02	*a* ^2^G_4½_—*v* ${\;}^{2}G_{4½}^{{}^{\circ}}$	6, 7*b*
3907.815	1	25582.53	−0.10	*b* ^4^G_3½_— $63319_{3½}^{{}^{\circ}}$	
3909.795	3H	25569.57	−0.03	*b* ^4^D_3½_— $55923_{2½}^{{}^{\circ}}$	
3910.222	5H	25566.78	0.00	*z* ${\;}^{4}P_{2½}^{{}^{\circ}}$—*i* ^6^D_1½_	
3911.144	20*hw*	25560.75	−0.05	*z* ${\;}^{4}P_{2½}^{{}^{\circ}}$—*e* ^4^D_2½_	
3911.425	20*c*	25558.92	−0.04	*b* ^4^P_2½_—*u* ${\;}^{4}P_{1½}^{{}^{\circ}}$	6, 7*b*
3912.553	1	25551.55	−0.01	*b* ^4^G_3½_—*x* ${\;}^{2}F_{3½}^{{}^{\circ}}$	
3912.754	5	25550.24	−0.14	*b* ^4^P_0½_—*z* ${\;}^{2}D_{1½}^{{}^{\circ}}$	4
3914.210	5H*lwd*	25540.73	−0.38	*z* ${\;}^{4}P_{0½}^{{}^{\circ}}$—*i* ^6^D_0½_	
3916.407	6*h*	25526.40	0.00	*b* ^4^P_1½_—*w* ${\;}^{4}D_{1½}^{{}^{\circ}}$	6
3916.601	10H*w*	25525.14	−0.07	*z* ${\;}^{4}P_{1½}^{{}^{\circ}}$—*e* ^4^D_1½_	
3918.319	60*c*	25513.95	$\left\{ \;\begin{array}{l} -0.11\\ -0.07 \end{array}\right.$	*b* ^4^P_2½_—*w* ${\;}^{4}D_{3½}^{{}^{\circ}}$ *a* ^4^H_6½_—*x* ${\;}^{4}G_{5½}^{{}^{\circ}}$	44
3919.332	3	25507.36	−0.16	*a* ^2^G_3½_—*v* ${\;}^{2}G_{3½}^{{}^{\circ}}$	6, 7*b*
3920.657	1	25498.74	−0.11	*b* ^4^G_2½_—*x* ${\;}^{2}F_{3½}^{{}^{\circ}}$	
3921.766	30H*w*	25491.52	0.01	*z* ${\;}^{4}P_{1½}^{{}^{\circ}}$—*i* ^6^D_1½_	P–B
3922.066	10H	25489.57	−0.07	*z* ${\;}^{4}P_{2½}^{{}^{\circ}}$—*i* ^6^D_2½_	P–B
3922.681	40H*w*	25485.58	0.05	*z* ${\;}^{4}P_{1½}^{{}^{\circ}}$—*e* ^4^D_2½_	
3923.325	30	25481.39	−0.03	*a* ^4^H_5½_—*x* ${\;}^{4}G_{4½}^{{}^{\circ}}$	4
3924.075	40H	25476.52	−0.16	*z* ${\;}^{4}P_{0½}^{{}^{\circ}}$—*e* ^4^D_1½_	
3925.912	4	25464.60	−0.14	*b* ^4^P_2½_—*x* ${\;}^{4}F_{3½}^{{}^{\circ}}$	4
3926.476	100H*w*	25460.95	0.02	*z* ${\;}^{4}P_{2½}^{{}^{\circ}}$—*e* ^4^D_3½_	
3928.314	6	25449.03	−0.12	*a* ^2^H_5½_—*w* ${\;}^{4}H_{5½}^{{}^{\circ}}$	6
3929.248	30H*w*	25443.02	0.04	*z* ${\;}^{4}P_{0½}^{{}^{\circ}}$—*i* ^6^D_1½_	
3929.662	20	25440.30	−0.11	*a* ^4^H_4½_—*x* ${\;}^{4}G_{3½}^{{}^{\circ}}$	4
3931.517	10	25428.30	−0.01	*a* ^2^H_4½_—*z* ${\;}^{2}H_{4½}^{{}^{\circ}}$	6, 7*b*
3933.666	50H*w*	25414.42	0.05	*z* ${\;}^{4}P_{1½}^{{}^{\circ}}$—*i* ^6^D_2½_	
3935.544	10	25402.28	−0.10	*a* ^4^H_5½_—*x* ${\;}^{4}G_{5½}^{{}^{\circ}}$	6
3936.766	30	25394.40	−0.03	*a* ^4^H_3½_—*x* ${\;}^{4}G_{2½}^{{}^{\circ}}$	5
3937.763	10	25387.97	−0.07	*a* ^4^H_4½_—*x* ${\;}^{4}G_{4½}^{{}^{\circ}}$	6
3941.951	7	25360.99	−0.05	*a* ^4^H_3½_—*x* ${\;}^{4}G_{3½}^{{}^{\circ}}$	
3942.881	50H*wl*	25355.01	−0.05	*z* ${\;}^{4}P_{2½}^{{}^{\circ}}$—*i* ^6^D_3½_	4
3943.719	1	25349.63	−0.14	*b* ^4^G_2_*_½_*—*x* ${\;}^{2}F_{2½}^{{}^{\circ}}$	
3951.977	20	25296.66	−0.06	*b* ^4^P_0½_—*w* ${\;}^{4}D_{0½}^{{}^{\circ}}$	6
3952.842	100	25291.12	0.01	*b* ^4^P_2½_—*u* ${\;}^{4}P_{2½}^{{}^{\circ}}$	6
3954.579	10	25280.01	−0.07	*a* ^2^H_5½_—*z* ${\;}^{2}H_{5½}^{{}^{\circ}}$	6
3955.314	1	25275.31	0.04	*a* ^2^H_4½_—*w* ${\;}^{4}H_{3½}^{{}^{\circ}}$	
3958.603	1*h*	25254.32	−0.03	*a* ^2^H_4½_— $63374_{4½}^{{}^{\circ}}$	
3959.138	1	25250.90	0.18	*e* ^6^S_2½_— $66654_{2½}^{{}^{\circ}}$	
3962.776	1	25227.73	0.00	*a* ^2^H_4½_—*y* ${\;}^{2}H_{4½}^{{}^{\circ}}$	
3972.077	1	25168.65	$\{\;\begin{array}{c} 0.05\\ 0.05 \end{array}$	*a* ^2^H_4½_—*z* ${\;}^{2}H_{5½}^{{}^{\circ}}$ *a* ^2^H_4½_—*x* ${\;}^{2}F_{3½}^{{}^{\circ}}$	
3975.880	40	25144.58	0.07	*b* ^4^P_0½_—*w* ${\;}^{4}D_{1½}^{{}^{\circ}}$	4
3977.076	50	25137.02	0.04	*b* ^4^P_1½_—*w* ${\;}^{4}D_{2½}^{{}^{\circ}}$	4
3978.781	2*h*	25126.24	0.00	*a* ^4^H_4½_—*v* ${\;}^{4}D_{3½}^{{}^{\circ}}$	
3980.142	10	25117.65	0.03	*a* ^2^G_4½_—*u* ${\;}^{2}F_{3½}^{{}^{\circ}}$	4
3982.164	30	25104.90	−0.02	*b* ^4^P_1½_—*u* ${\;}^{4}P_{0½}^{{}^{\circ}}$	
3982.576	100	25102.30	0.07	*a* ^4^G_2½_—*y* ${\;}^{4}F_{1½}^{{}^{\circ}}$	P–B
3982.900	40	25100.26	−0.02	*e* ^6^S_2½_— $66504_{1½}^{{}^{\circ}}$	
3984.172	20	25092.25	0.06	*a* ^4^G_2½_—*y* ${\;}^{4}F_{2½}^{{}^{\circ}}$	
3985.236	100	25085.55	0.06	*a* ^4^G_3½_—*y* ${\;}^{4}F_{2½}^{{}^{\circ}}$	P–B
3986.377	2	25078.34	0.10	*a* ^4^G_2½_—*y* ${\;}^{4}F_{3½}^{{}^{\circ}}$	
3986.822	200	25075.57	0.01	*a* ^4^G_5½_—*y* ${\;}^{4}F_{4½}^{{}^{\circ}}$	4
3987.092	100	25073.87	0.02	*a* ^4^G_4½_—*y* ${\;}^{4}F_{3½}^{{}^{\circ}}$	4
3987.455	20	25071.59	0.05	*a* ^4^G_3½_—*y* ${\;}^{4}F_{3½}^{{}^{\circ}}$	P–B
3988.666	8	25063.98	−0.01	*b* ^4^P_1½_—*v* ${\;}^{4}D_{0½}^{{}^{\circ}}$	P–B
3989.699	1	25057.49	−0.04	*a* ^4^H_3½_—*v* ${\;}^{4}D_{2½}^{{}^{\circ}}$	
3989.952	30	25055.90	0.03	*a* ^4^G_4½_—*y* ${\;}^{4}F_{4½}^{{}^{\circ}}$	6
3990.737	1	25050.97	0.04	*b* ^4^D_3½_—*w* ${\;}^{4}P_{2½}^{{}^{\circ}}$	
3991.596	20	25045.58	0.12	*b* ^4^D_0½_—*w* ${\;}^{4}P_{0½}^{{}^{\circ}}$	
3993.858	6	25031.40	−0.09	*b* ^4^D_1½_—*w* ${\;}^{4}P_{0½}^{{}^{\circ}}$	P–B
3995.012	1*h*	25024.17	0.06	*b* ^4^G_3½_—*w* ${\;}^{6}D_{2½}^{{}^{\circ}}$?	
3996.101	5H*s*	25017.34	−0.09	*b* ^4^P_1½_—*v* ${\;}^{4}D_{2½}^{{}^{\circ}}$	4
3997.771	3*h*	25006.89	−0.03	*a* ^4^H_5½_—*x* ${\;}^{4}F_{4½}^{{}^{\circ}}$	
3999.573	1*h*	24995.63	−0.02	*a* ^4^H_4½_—*w* ${\;}^{4}D_{3½}^{{}^{\circ}}$	
4001.190	15	24985.52	−0.01	*b* ^4^D_2½_—*w* ${\;}^{4}P_{2½}^{{}^{\circ}}$	P–B
4002.169	15	24979.42	−0.01	*b* ^4^D_1½_—*w* ${\;}^{4}P_{2½}^{{}^{\circ}}$	P–B
4002.559	1	24976.98	0.05	*a* ^4^F_1½_—*w* ${\;}^{4}D_{0½}^{{}^{\circ}}$	
4003.258	20H*l*	24972.63	0.05	*b* ^4^G_5½_—*v* ${\;}^{4}F_{4½}^{{}^{\circ}}$	
4005.775	1	24956.93	0.01	*b* ^4^D_0½_—*w* ${\;}^{4}P_{1½}^{{}^{\circ}}$	
4007.041	10	24949.04	−0.01	*b* ^4^D_2½_—*w* ${\;}^{4}P_{1½}^{{}^{\circ}}$	P–B
4008.022	20	24942.94	−0.01	*b* ^4^D_1½_—*w* ${\;}^{4}P_{1½}^{{}^{\circ}}$	P–B
4011.535	30	24921.09	0.01	*b* ^4^P_1½_—*u* ${\;}^{4}P_{1½}^{{}^{\circ}}$	6
4011.913	10*h*	24918.75	−0.05	*a* ^2^G_3½_—*u* ${\;}^{2}F_{3½}^{{}^{\circ}}$	6
4012.761	2	24913.48	−0.06	*a* ^4^H_4½_—*x* ${\;}^{4}F_{4½}^{{}^{\circ}}$	
4016.671	5	24889.23	0.05	*a* ^4^F_4½_—*z* ${\;}^{2}I_{5½}^{{}^{\circ}}$	4
4018.106	1000*h*	24880.34	−0.01	*a* ^6^D_4½_—*z* ${\;}^{6}D_{3½}^{{}^{\circ}}$	4
4018.583	2*h*	24877.39	−0.05	*a* ^2^G_4½_—*v* ${\;}^{4}G_{4½}^{{}^{\circ}}$	
4020.072	10	24868.17	0.00	*b* ^4^D_0½_—*x* ${\;}^{4}D_{1½}^{{}^{\circ}}$	5
4021.354	2	24860.25	−0.05	*b* ^4^D_2½_—*x* ${\;}^{4}D_{1½}^{{}^{\circ}}$	
4022.335	2*h*	24854.18	$\left\{ \;\begin{array}{l} -0.01\\ -0.12 \end{array}\right.$	*b* ^4^D_1½_—*x* ${\;}^{4}D_{1½}^{{}^{\circ}}$ *a* ^2^F_3½_— $63523_{2½}^{{}^{\circ}}$	
4023.719	1	24845.63	0.02	*a* ^4^F_4½_—*x* ${\;}^{4}G_{3½}^{{}^{\circ}}$	
4025.938	4	24831.94	−0.02	*b* ^4^D_3½_—*x* ${\;}^{4}D_{2½}^{{}^{\circ}}$	5
4026.437	80	24828.86	0.00	*a* ^4^G_5½_—*z* ${\;}^{4}H_{6½}^{{}^{\circ}}$	4
4028.595	5H	24815.56	−0.01	*a* ^4^G_5½_—*z* ${\;}^{4}H_{5½}^{{}^{\circ}}$	6
4030.755	20000R*w*	24802.26	0.01	*a* ^6^S_2½_—*z* ${\;}^{6}P_{3½}^{{}^{\circ}}$	4
4031.791	100	24795.89	0.01	*a* ^4^G_4½_—*z* ${\;}^{4}H_{5½}^{{}^{\circ}}$	
4033.068	15000R*w*	24788.04	−0.01	*a* ^6^S_2½_—*z* ${\;}^{6}P_{2½}^{{}^{\circ}}$	6
4033.587	4	24784.85	0.00	*a* ^4^G_3½_—*z* ${\;}^{4}H_{4½}^{{}^{\circ}}$	
4033.652	3	24784.45	0.03	*a* ^4^G_2½_—*z* ${\;}^{4}H_{3½}^{{}^{\circ}}$	
4034.485	10000R*w*	24779.33	0.01	*a* ^6^S_2½_—*z* ${\;}^{6}P_{1½}^{{}^{\circ}}$	4
4035.729	1000	24771.70	−0.03	*a* ^6^D_3½_—*z* ${\;}^{6}D_{2½}^{{}^{\circ}}$	4
4036.244	4	24768.51	−0.22	*a* ^2^P_1½_—*z* ${\;}^{2}P_{1½}^{{}^{\circ}}$	
4036.562	5	24766.58	0.02	*b* ^4^D_2½_—*x* ${\;}^{4}D_{2½}^{{}^{\circ}}$	
4037.561	5	24760.45	−0.01	*b* ^4^D_1½_—*x* ${\;}^{4}D_{2½}^{{}^{\circ}}$	
4038.728	20	24753.30	−0.01	*b* ^4^D_3½_—*x* ${\;}^{4}D_{3½}^{{}^{\circ}}$	6, 7*b*
4039.250	1H*l*	24750.10	−0.04	*b* ^4^G_3½_—*v* ${\;}^{4}F_{2½}^{{}^{\circ}}$	
4040.424	2	24742.95	0.01	*a* ^4^F_3½_—*x* ${\;}^{4}G_{3½}^{{}^{\circ}}$	
4041.357	2000*h*	24737.20	0.01	*a* ^6^D_4½_—*z* ${\;}^{6}D_{4½}^{{}^{\circ}}$	6, 7*b*
4043.682	1*h*	24722.98	−0.05	*b* ^4^P_0½_—*u* ${\;}^{4}P_{0½}^{{}^{\circ}}$	
4045.115	50	24714.22	0.02	*a* ^4^F_4½_—*x* ${\;}^{4}G_{5½}^{{}^{\circ}}$	4
4045.203	50	24713.68	−0.04	*a* ^4^H_6½_—*z* ${\;}^{4}I_{7½}^{{}^{\circ}}$	4
4046.711	1*h*	24704.47	−0.04	*a* ^4^H_6½_—*z* ${\;}^{4}I_{6½}^{{}^{\circ}}$	
4046.996	3	24702.73	0.01	*a* ^4^F_2½_—*x* ${\;}^{4}G_{2½}^{{}^{\circ}}$	6
4048.747	1000*h*	24692.05	0.00	*a* ^6^D_2½_—*z* ${\;}^{6}D_{1½}^{{}^{\circ}}$	4, C
4048.998	40	24690.52	−0.05	*a* ^4^F_3½_—*X* ${\;}^{4}G_{4½}^{{}^{\circ}}$	4
4049.423	1*h*	24687.93	0.02	*b* ^4^D_2½_—*x* ${\;}^{4}D_{3½}^{{}^{\circ}}$	
4050.306	1	24682.54	−0.09	*b* ^4^P_0½_—*V* ${\;}^{4}D_{1½}^{{}^{\circ}}$	
4051.255	2*h*	24676.76	−0.03	*a* ^4^D_2½_—*y* ${\;}^{6}F_{3½}^{{}^{\circ}}$	
4052.476	50	24669.33	0.00	*a* ^4^F_2½_—*X* ${\;}^{4}G_{3½}^{{}^{\circ}}$	4
4055.215	20	24652.67	0.02	*a* ${\;}^{4}F_{1½}$—*x* ${\;}^{4}G_{2½}^{{}^{\circ}}$	5
4055.548	1000*h*	24650.64	0.00	*a* ^6^D_3½_—*z* ${\;}^{6}D_{3½}^{{}^{\circ}}$	6, 7*b*
4057.954	100*hl*	24636.03	0.00	*z* ${\;}^{6}P_{1½}^{{}^{\circ}}$—*f* ^6^S_2½_	4
4058.936	500	24630.06	−0.02	*a* ^6^D_1½_—*z* ${\;}^{6}D_{0½}^{{}^{\circ}}$	4, C
4059.388	150*hl*	24627.32	0.02	*z* ${\;}^{6}P_{2½}^{{}^{\circ}}$—*f* ^6^S_2½_	6
4061.737	200*hl*	24613.09	−0.01	*z* ${\;}^{6}P_{3½}^{{}^{\circ}}$—*f* ^6^S_2½_	4
4063.530	400	24602.22	0.01	*a* ^6^D_2½_—*z* ${\;}^{6}D_{2½}^{{}^{\circ}}$	6, 7*b*
4065.083	100	24592.82	−0.05	*a* ^4^H_5½_—*z* ${\;}^{4}I_{6½}^{{}^{\circ}}$	
4066.235	10	24585.86	−0.03	*a* ^2^G_4½_—*v* ${\;}^{2}F_{3½}^{{}^{\circ}}$	5
4067.238	5	24579.79	0.00	*a* ^2^I_6½_— $61744_{5½}^{{}^{\circ}}$	
4068.011	100	24575.10	0.01	*a* ^6^D_1½_—*z* ${\;}^{6}D_{1½}^{{}^{\circ}}$	6, 7*b*
4070.280	200	24561.42	0.01	*a* ^6^D_0½_—*z* ${\;}^{6}D_{0½}^{{}^{\circ}}$	6, 7C
4070.682	2*h*	24559.00	0.02	*a* ^4^F_3½_—*w* ${\;}^{4}D_{2½}^{{}^{\circ}}$	
4071.949	1	24551.36	−0.03	*a* ^4^D_1½_—*y* ${\;}^{6}F_{2½}^{{}^{\circ}}$	
4073.976	10	24539.14	−0.05	*b* ^4^P_0½_—*u* ${\;}^{4}P_{1½}^{{}^{\circ}}$	4
4075.251	40*h*	24531.46	0.02	*a* ^4^F_4½_—*v* ${\;}^{4}D_{3½}^{{}^{\circ}}$	4
4076.708	2	24522.70	−0.06	*a* ^4^H_4½_—*z* ${\;}^{4}I_{4½}^{{}^{\circ}}$	
4079.241	500	24507.47	$\left\{ \;\begin{array}{l} -0.01\\ -0.12 \end{array}\right.$	*a* ^6^D_3½_—*z* ${\;}^{6}D_{4½}^{{}^{\circ}}$ *a* ^4^H_4½_—*z* ${\;}^{4}I_{5½}^{{}^{\circ}}$	5
4079.415	500	24506.34	−0.08	*a* ^6^D_0½_—*z* ${\;}^{6}D_{1½}^{{}^{\circ}}$	4, C
4082.945	600	24485.24	−0.01	*a* ^6^D_1½_—*z* ${\;}^{6}D_{2½}^{{}^{\circ}}$	4, C
4083.634	500	24481.11	−0.01	*a* ^6^D_2½_—*z* ${\;}^{6}D_{3½}^{{}^{\circ}}$	4
4085.480	1	24470.04	−0.06	*a* ^2^F_3½_—*x* ${\;}^{2}F_{2½}^{{}^{\circ}}$	
4087.073	1	24460.51	0.00	*a* ^2^F_2½_—*w* ${\;}^{4}H_{3½}^{{}^{\circ}}$	
4087.617	1H	24457.25	−0.07	*a* ^4^D_3½_—*y* ${\;}^{6}D_{2½}^{{}^{\circ}}$	
4088.565	6	24451.58	−0.02	*b* ^2^I_6½_—*w* ${\;}^{2}H_{5½}^{{}^{\circ}}$	
4089.941	60	24443.36	−0.03	*a* ^4^H_3½_—*Z* ${\;}^{4}I_{4½}^{{}^{\circ}}$	5
4090.618	10	24439.31	−0.12	*a* ^4^F_3½_—*v* ${\;}^{4}D_{2½}^{{}^{\circ}}$	4
4090.910	3	24437.57	0.00	*b* ^2^I_5½_—*w* ${\;}^{2}H_{4½}^{{}^{\circ}}$	
4092.388	40*h*	24428.74	−0.03	*a* ^4^F_3½_—*v* ${\;}^{4}D_{3½}^{{}^{\circ}}$	6
4094.070	6	24418.70	−0.13	*a* ^2^G_3½_—*v* ${\;}^{2}F_{2½}^{{}^{\circ}}$	
4095.052	10	24412.85	−0.06	*a* ^4^F_2½_—*v* ${\;}^{4}D_{1½}^{{}^{\circ}}$	4
4095.252	30	24411.66	−0.02	*a* ^2^F_3½_—*y* ${\;}^{2}D_{2½}^{{}^{\circ}}$	4
4096.676	5	24403.17	−0.25	*b* ^4^G_4½_—*y* ${\;}^{2}F_{3½}^{{}^{\circ}}$	
4099.399	8	24386.96	−0.11	*a* ^2^G_3½_—*v* ${\;}^{2}F_{3½}^{{}^{\circ}}$	6
4099.899	5*h*	24383.99	−0.13	*a* ^2^H_5½_—*v* ${\;}^{4}F_{4½}^{{}^{\circ}}$	
4102.970	30	24365.74	−0.08	*a* ^4^F_2½_—*v* ${\;}^{4}D_{2½}^{{}^{\circ}}$	6
4103.463	10	24362.81	−0.03	*a* ^4^F_1½_—*v* ${\;}^{4}D_{1½}^{{}^{\circ}}$	P–B
4103.560	5	24362.22	−0.09	*a* ^4^F_1½_—*v* ${\;}^{4}D_{0½}^{{}^{\circ}}$	P–B
4105.365	60*c*	24351.52	−0.01	*a* ^4^F_4½_—*x* ${\;}^{4}F_{3½}^{{}^{\circ}}$	5
4107.873	10	24336.66	−0.02	*a* ^2^I_5½_—*w* ${\;}^{4}G_{4½}^{{}^{\circ}}$	4
4110.802	30	24319.32	−0.03	*a* ^4^F_3½_—*x* ${\;}^{4}F_{2½}^{{}^{\circ}}$	
4110.894	60*c*	24318.77	0.03	*a* ^4^F_4½_—*x* ${\;}^{4}F_{4½}^{{}^{\circ}}$	6, 7
4111.416	2	24315.68	−0.07	*a* ^4^F_1½_—*v* ${\;}^{4}D_{2½}^{{}^{\circ}}$	
4113.243	30	24304.88	$\left\{ \;\begin{array}{r} 0.10\\ -0.08 \end{array}\right.$	*a* ^4^P_1½_—*x* ${\;}^{4}P_{0½}^{{}^{\circ}}$ (*a* ^2^I_6½_—*w* ${\;}^{4}G_{5½}^{{}^{\circ}}$)	4
4113.880	20	24301.12	−0.05	*a* ^4^F_2½_—*x* ${\;}^{4}F_{1½}^{{}^{\circ}}$	5
4114.381	25	24298.16	−0.02	*a* ^4^F_3½_—*w* ${\;}^{4}D_{3½}^{{}^{\circ}}$	6
4114.594	10H*l*	24296.90	0.08	*b* ^4^G_3½_—*y* ${\;}^{2}F_{3½}^{{}^{\circ}}$	
4115.033	1*h*	24294.31	0.03	*b* ^4^G_5½_—*y* ${\;}^{2}G_{4½}^{{}^{\circ}}$	
4116.598	8H*l*	24285.08	−0.01	*b* ^4^G_2½_—*y* ${\;}^{2}F_{2½}^{{}^{\circ}}$	6, 7*b*
4119.010	5	24270.85	−0.08	*a* ^4^P_0½_—*x* ${\;}^{4}P_{0½}^{{}^{\circ}}$	
4122.367	20	24251.09	−0.01	*a* ^4^F_1½_—*x* ${\;}^{4}F_{1½}^{{}^{\circ}}$	
4122.757	12	24248.80	−0.06	*a* ^4^F_3½_—*x* ${\;}^{4}F_{3½}^{{}^{\circ}}$	
4123.280	15	24245.72	−0.02	*a* ^4^F_2½_—*x* ${\;}^{4}F_{2½}^{{}^{\circ}}$	
4123.542	20	24244.18	0.07	*a* ^4^P_2½_—*x* ${\;}^{4}P_{1½}^{{}^{\circ}}$	
4125.415	1*h*	24233.17	−0.06	*a* ^4^D_2½_—*w* ${\;}^{6}P_{1½}^{{}^{\circ}}$	
4125.813	8*c*	24230.83	0.03	*a* ^2^G_4½_—*w* ${\;}^{2}G_{4½}^{{}^{\circ}}$	6
4126.710	1*h*	24225.57	0.25	*a* ^4^D_2½_—*y* ${\;}^{6}D_{3½}^{{}^{\circ}}$	
4126.876	1	24224.60	0.03	*a* ^4^F_2½_—*w* ${\;}^{4}D_{3½}^{{}^{\circ}}$	
4127.751	1	24219.46	0.06	*a* ^4^F_1½_—*u* ${\;}^{4}P_{1½}^{{}^{\circ}}$	
4130.256	2*h*	24204.77	$\left\{ \;\begin{array}{r} -0.02\\ 0.01 \end{array}\right.$	*a* ^4^D_2½_—*y* ${\;}^{6}D_{2½}^{{}^{\circ}}$ *a* ^2^F_2½_—*x* ${\;}^{2}F_{2½}^{{}^{\circ}}$	
4131.111	150	24199.76	−0.03	*a* ^4^H_6½_—*y* ${\;}^{4}H_{6½}^{{}^{\circ}}$	6, 7*b*
4131.449	10	24197.78	0.13	*a* ^4^F_1½_—*x* ${\;}^{4}P_{1½}^{{}^{\circ}}$	6, 7*b*
4132.282	6	24192.90	0.13	*z* ${\;}^{4}F_{3½}^{{}^{\circ}}$—*f* ${\;}^{4}G_{4½}^{{}^{\circ}}$	4
4134.619	20	24179.23	0.06	*a* ^2^F_2½_—*y* ${\;}^{2}D_{1½}^{{}^{\circ}}$	5
4135.034	100	24176.80	0.02	*a* ^4^H_5½_—*y* ${\;}^{4}H_{5½}^{{}^{\circ}}$	6, 7*b*
4137.266	40	24163.76	−0.04	*a* ^4^P_0½_—*x* ${\;}^{4}P_{1½}^{{}^{\circ}}$	4
4141.063	100	24141.60	−0.02	*a* ^4^H_4½_—*y* ${\;}^{4}H_{4½}^{{}^{\circ}}$	6, 7*b*
4147.529	60	24103.97	0.10	*a* ^4^P_2½_—*x* ${\;}^{4}P_{2½}^{{}^{\circ}}$	6, 7*b*
4148.796	80	24096.61	−0.02	*a* ^4^H_3½_—*y* ${\;}^{4}H_{3½}^{{}^{\circ}}$	6, 7*b*
4149.648	1H	24091.66	−0.07	*a* ^2^F_3½_—*w* ${\;}^{6}D_{2½}^{{}^{\circ}}$	
4150.262	1	24088.10	−0.05	*a* ^4^H_5½_—*y* ${\;}^{4}H_{6½}^{{}^{\circ}}$	
4151.023	10h*l*	24083.68	$\left\{ \;\begin{array}{r} 0.28\\ -0.22 \end{array}\right.$	*a* ^4^H_4½_—*y* ${\;}^{4}H_{5½}^{{}^{\circ}}$ *b* ^4^G_4½_—*y* ${\;}^{2}G_{4½}^{{}^{\circ}}$	
4151.663	3*h*	24079.97	−0.39	*b* ^4^G_4½_—*z* ${\;}^{2}F_{3½}^{{}^{\circ}}$	
4152.556	1	24074.79	−0.04	*a* ^2^G_3½_—*w* ${\;}^{2}G_{3½}^{{}^{\circ}}$	
4154.218	1	24065.16	0.06	*b* ^4^G_5½_—*w* ${\;}^{4}G_{4½}^{{}^{\circ}}$	
4154.631	2	24062.76	−0.15	*a* ^4^D_1½_—*w* ${\;}^{6}P_{1½}^{{}^{\circ}}$	
4154.726	1	24062.22	−0.03	*a* ^4^H_3½_—*y* ${\;}^{4}H_{4½}^{{}^{\circ}}$	
4155.525	40	24057.59	0.18	*a* ^4^P_1½_—*x* ${\;}^{4}P_{2½}^{{}^{\circ}}$	4
4157.019	60	24048.94	$\left\{ \;\begin{array}{r} -0.03\\ 0.22 \end{array}\right.$	*b* ^4^G_5½_—*w* ${\;}^{4}G_{5½}^{{}^{\circ}}$ *b* ^4^G_3½_—*y* ${\;}^{2}G_{3½}^{{}^{\circ}}$?	6
4158.692	8	24039.24	−0.03	*a* ^2^P_0½_—*z* ${\;}^{2}P_{0½}^{{}^{\circ}}$	6
4159.957	1	24031.96	−0.02	*a* ^2^G_3½_—*w* ${\;}^{2}G_{4½}^{{}^{\circ}}$	
4164.979	10*h*	24002.96	−0.02	*a* ^2^P_0½_—*z* ${\;}^{2}P_{1½}^{{}^{\circ}}$	5
4166.208	6	23995.90	−0.11	*b* ^4^G_2½_—*y* ${\;}^{2}G_{3½}^{{}^{\circ}}$	
4167.197	2*h*	23990.21	−0.03	*b* ^4^G_3½_—*z* ${\;}^{2}F_{2½}^{{}^{\circ}}$	
4169.440	1*h*	23977.30	0.00	*b* ^4^G_3½_—*y* ${\;}^{2}G_{4½}^{{}^{\circ}}$	
4175.891	2*h*	23940.26	0.26	*a* ^4^D_1½_—*w* ${\;}^{6}P_{2½}^{{}^{\circ}}$	
4176.608	100*c*	23936.15	−0.03	*a* ^4^H_6½_—*y* ${\;}^{4}G_{5½}^{{}^{\circ}}$	4
4179.875	4*h*	23917.44	−0.02	*a* ^4^D_2½_—*y* ${\;}^{6}D_{1½}^{{}^{\circ}}$	
4182.254	20H*w*	23903.84	−0.03	*a* ^2^H_5½_—*y* ${\;}^{2}I_{6½}^{{}^{\circ}}$	
4184.956	2*h*	23888.40	−0.14	*e* ^8^S_3½_— $63319_{3½}^{{}^{\circ}}$	
4189.990	100	23859.70	−0.02	*a* ^4^H_5½_—*y* ${\;}^{4}G_{4½}^{{}^{\circ}}$	4
4190.901	1*h*	23854.51	−0.21	*b* ^4^G_4½_—*w* ${\;}^{4}G_{4½}^{{}^{\circ}}$	
4193.745	1	23838.34	$\left\{ \;\begin{array}{l} -0.08\\ -0.25 \end{array}\right.$	*a* ^4^D_3½_—*w* ${\;}^{6}P_{3½}^{{}^{\circ}}$ *b* ^4^G_4½_—*w* ${\;}^{4}G_{5½}^{{}^{\circ}}$	
4199.250	1*h*	23807.09	−0.13	*a* ^2^I_5½_—*x* ${\;}^{4}H_{4½}^{{}^{\circ}}$	
4201.778	60	23792.77	−0.02	*a* ^4^H_4½_—*y* ${\;}^{4}G_{3½}^{{}^{\circ}}$	4
4202.612	1	23788.05	0.00	*a* ^2^G_4½_—*v* ${\;}^{4}H_{5½}^{{}^{\circ}}$	
4203.113	3	23785.21	0.14	*a* ^2^I_5½_—*x* ${\;}^{4}H_{5½}^{{}^{\circ}}$	
4207.956	1*hl*	23757.84	−0.08	*a* ^2^G_3½_—*w* ${\;}^{2}F_{3½}^{{}^{\circ}}$	
4209.677	2	23748.12	0.00	*b* ^4^G_3__½_—*w* ${\;}^{4}G_{4½}^{{}^{\circ}}$	
4209.864	1*h*	23747.07	−0.07	*a* ^4^D_1__½_—*y* ${\;}^{6}D_{1½}^{{}^{\circ}}$	
4210.519	2*h*	23743.37	−0.01	*b* ^4^G_3__½_—*w* ${\;}^{4}G_{3½}^{{}^{\circ}}$	
4211.753	50	23736.42	−0.04	*a* ^4^H_3__½_—*y* ${\;}^{4}G_{2½}^{{}^{\circ}}$	5
4211.942	10H*l*	23735.35	0.01	*a* ^2^H_5__½_— $61744_{5½}^{{}^{\circ}}$	
4212.443	8	23732.53	−0.11	*a* ^4^D_1__½_—*y* ${\;}^{6}D_{0½}^{{}^{\circ}}$	
4215.842	1	23713.40	−0.02	*a* ^4^H_3__½_—*y* ${\;}^{4}G_{3½}^{{}^{\circ}}$	
4217.186	1*h*	23705.84	0.02	*a* ^2^H_5__½_—*y* ${\;}^{2}G_{4½}^{{}^{\circ}}$	
4218.405	5H*w*	23698.99	0.10	*a* ^2^H_4__½_—*y* ${\;}^{2}I_{5½}^{{}^{\circ}}$	
4220.610	50*cw*	23686.61	−0.06	*b* ^4^P_2__½_—*y* ${\;}^{4}S_{1½}^{{}^{\circ}}$	4
4221.562	9*h*	23681.27	−0.03	*b* ^4^G_2__½_—*w* ${\;}^{4}G_{2½}^{{}^{\circ}}$	
4224.336	5	23665.72	−0.04	*a* ^2^H_4__½_—*y* ${\;}^{2}G_{3½}^{{}^{\circ}}$	
4225.080	2	23661.55	−0.04	*b* ^4^P_2__½_—*v* ${\;}^{4}P_{2½}^{{}^{\circ}}$	
4225.785	1	23657.60	−0.10	*a* ^2^G_3__½_—*v* ${\;}^{4}H_{4½}^{{}^{\circ}}$	
4230.144	10	23633.22	−0.07	*a* ^4^D_0__½_—*y* ${\;}^{6}D_{0½}^{{}^{\circ}}$	
4235.154	400	23605.27	−0.04	*a* ^4^D_2__½_—*y* ${\;}^{4}P_{1½}^{{}^{\circ}}$	4
4235.300	800	23604.45	−0.01	*a* ^4^D_3__½_—*y* ${\;}^{4}P_{2½}^{{}^{\circ}}$	4
4237.104	2H	23594.41	0.07	*a* ^2^H_4__½_—*y* ${\;}^{2}G_{4½}^{{}^{\circ}}$	
4239.737	200	23579.75	−0.02	*a* ^4^D_1__½_—*y* ${\;}^{4}P_{0½}^{{}^{\circ}}$	4
4247.689	1	23535.61	−0.03	*b* ^4^G_5__½_—*x* ${\;}^{4}H_{4½}^{{}^{\circ}}$	
4250.722	1*h*	23518.82	0.09	*b* ^4^G_5__½_—*w* ${\;}^{4}F_{4½}^{{}^{\circ}}$	
4251.345	2*hw*	23515.37	0.09	*b* ^2^I_6__½_—*u* ${\;}^{4}H_{6½}^{{}^{\circ}}$	
4257.669	200	23480.44	0.02	*a* ^4^D_0½_—*y* ${\;}^{4}P_{0½}^{{}^{\circ}}$	6
4258.369	7	23476.58	−0.06	*a* ^2^H_5__½_—*w* ${\;}^{4}G_{4½}^{{}^{\circ}}$	
4259.349	10	23471.18	−0.06	*b* ^4^G_5__½_—*x* ${\;}^{4}H_{6½}^{{}^{\circ}}$	4
4261.301	30	23460.43	−0.08	*a* ^2^H_5__½_—*w* ${\;}^{4}G_{5½}^{{}^{\circ}}$	6
4265.928	400	23434.99	0.00	*a* ^4^D_1½_—*y* ${\;}^{4}P_{1½}^{{}^{\circ}}$	
4271.282	2	23405.61	0.19	*a* ^2^F_3__½_—*y* ${\;}^{2}F_{2½}^{{}^{\circ}}$	
4278.676	20	23365.16	0.00	*a* ${\;}^{2}H_{4½}^{{}^{\circ}}$—*w* ${\;}^{4}G_{4½}^{{}^{\circ}}$	6
4279.545	8H	23360.42	0.00	*a* ${\;}^{2}H_{4½}^{{}^{\circ}}$—*w* ${\;}^{4}G_{3½}^{{}^{\circ}}$	
4281.100	500	23351.93	0.00	*a* ^4^D_2½_—*y* ${\;}^{4}P_{2½}^{{}^{\circ}}$	6
4284.083	100	23335.67	0.03	*a* ^4^D_0½_—*y* ${\;}^{4}P_{1½}^{{}^{\circ}}$	5
4290.111	10	23302.89	−0.22	*b* ^4^G_4__½_—*x* ${\;}^{4}H_{5½}^{{}^{\circ}}$	4
4300.194	20	23248.25	0.00	*b* ^4^G_5__½_—*z* ${\;}^{2}G_{4½}^{{}^{\circ}}$	5
4305.670	10	23218.68	0.02	*b* ^4^G_3__½_—*x* ${\;}^{4}H_{4½}^{{}^{\circ}}$	
4305.985	4	23216.98	$\{\begin{array}{r} \begin{array}{r} -0.09\\ 0.13 \end{array} \end{array}$	*a* ^2^H_5__½_—*y* ${\;}^{4}I_{5½}^{{}^{\circ}}$ *a* ^2^H_5__½_—*y* ${\;}^{4}I_{6½}^{{}^{\circ}}$	
4308.633	1	23202.71	−0.02	*a* ^2^H_5__½_—*y* ${\;}^{4}I_{4½}^{{}^{\circ}}$	
4312.554	100	23181.62	0.01	*a* ^4^D_1__½_—*y* ${\;}^{4}P_{2½}^{{}^{\circ}}$	5
4314.424	2*h*	23171.57	0.03	*a* ^4^F_4__½_—*y* ${\;}^{4}G_{4½}^{{}^{\circ}}$	
4315.235	8	23167.22	−0.06	*b* ^4^G_2½_—*x* ${\;}^{4}H_{3½}^{{}^{\circ}}$	
4315.532	1*h*	23165.62	0.04	*b* ^4^G_3½_—*w* ${\;}^{4}F_{3½}^{{}^{\circ}}$	
4320.284	4H	23140.14	0.06	*a* ^2^F_2½_—*y* ${\;}^{2}F_{2½}^{{}^{\circ}}$	
4326.181	5	23108.60	−0.20	*b* ^4^G_4__½_—*z* ${\;}^{2}G_{3½}^{{}^{\circ}}$	
4326.747	1	23105.57	−0.02	*a* ^2^H_4½_—*y* ${\;}^{4}I_{5½}^{{}^{\circ}}$	
4327.951	10H*w*	23099.15	0.05	*a* ^2^F_2½_—*y* ${\;}^{2}F_{3½}^{{}^{\circ}}$	
4328.676	2	23095.28	−0.04	*a* ^4^F_3½_—*y* ${\;}^{4}G_{3½}^{{}^{\circ}}$	
4329.430	3	23091.26	0.01	*a* ^2^H_4½_—*y* ${\;}^{4}I_{4½}^{{}^{\circ}}$	
4330.938	1	23083.22	0.09	*b* ^4^G_3½_—*w* ${\;}^{4}F_{2½}^{{}^{\circ}}$	
4337.414	30*c*	23048.76	−0.03	*b* ^4^P_1½_—*y* ${\;}^{4}S_{1½}^{{}^{\circ}}$	
4338.135	2*h*	23044.92	0.00	*a* ^2^F_3½_—*y* ${\;}^{2}G_{4½}^{{}^{\circ}}$	
4338.819	3*h*	23041.29	−0.09	*a* ^2^F_3½_—*z* ${\;}^{2}F_{3½}^{{}^{\circ}}$	
4342.105	1	23023.85	$\{\begin{array}{r} \begin{array}{r} 0.14\\ -0.04 \end{array} \end{array}$	*b* ^4^P_1½_—*v* ${\;}^{4}P_{2½}^{{}^{\circ}}$ *a* ^4^G_4½_—*x* ${\;}^{2}H_{4½}^{{}^{\circ}}$	
4346.331	5*h*	23001.47	0.00	*z* ${\;}^{8}P_{2½}^{{}^{\circ}}$—*e* ^6^S_2½_	
4352.100	1?	22970.98	0.04	*b* ^4^G_2½_—*w* ${\;}^{4}F_{1½}^{{}^{\circ}}$	
4356.180	1*h*	22949.46	−0.03	*b* ^4^G_2½_—*z* ${\;}^{2}G_{3½}^{{}^{\circ}}$	
4356.613	2	22947.18	0.00	*a* ^2^H_5½_—*x* ${\;}^{4}H_{4½}^{{}^{\circ}}$	
4359.640	2	22931.25	−0.02	*b* ^4^G_3½_—*z* ${\;}^{2}G_{4½}^{{}^{\circ}}$	
4359.815	8*h*	22930.33	0.06	*a* ^2^H_5½_—*w* ${\;}^{4}F_{4½}^{{}^{\circ}}$	
4366.078	1	22897.44	0.03	*b* ^4^P_1½_—*v* ${\;}^{4}P_{1½}^{{}^{\circ}}$	
4368.881	50	22882.75	−0.03	*a* ^2^H_5½_—*x* ${\;}^{4}H_{6½}^{{}^{\circ}}$	
4370.881	8	22872.29	0.00	*z* ${\;}^{8}P_{3½}^{{}^{\circ}}$—*e* ^6^S_2½_	
4374.952	50	22851.00	$\{\begin{array}{r} \begin{array}{r} -0.16\\ 0.00 \end{array} \end{array}$	*a* ^4^P_1½_—*v* ${\;}^{6}P_{1½}^{{}^{\circ}}$ *a* ^2^F_2½_—*y* ${\;}^{2}G_{3½}^{{}^{\circ}}$	7*b*
4381.700	50	22815.80	0.06	*a* ^2^F_3½_—*w* ${\;}^{4}G_{4½}^{{}^{\circ}}$	4
4382.135	1	22813.54	−0.01	*a* ^2^H_4½_—*x* ${\;}^{4}H_{5½}^{{}^{\circ}}$	
4382.620	40H	22811.01	$\{\begin{array}{r} \begin{array}{r} 0.02\\ 0.01 \end{array} \end{array}$	*a* ^4^P_2½_—*v* ${\;}^{6}P_{2½}^{{}^{\circ}}$ *a* ^2^F_3½_—*w* ${\;}^{4}G_{3½}^{{}^{\circ}}$	6, 7*b*
4384.410	2*h*	22801.70	0.07	*a* ^2^F_3½_—*w* ${\;}^{4}G_{2½}^{{}^{\circ}}$	
4386.176	2*h*	22792.52	0.00	*a* ^2^F_2½_—*z* ${\;}^{2}F_{2½}^{{}^{\circ}}$	
4388.092	10	22782.57	−0.05	*a* ^2^H_4½_—*w* ${\;}^{4}F_{3½}^{{}^{\circ}}$	
4389.377	1*h*	22775.90	−0.14	*a* ^2^F_2½_—*z* ${\;}^{2}F_{3½}^{{}^{\circ}}$	
4390.540	2*h*	22769.87	−0.01	*b* ^4^D_3½_—*y* ${\;}^{4}D_{3½}^{{}^{\circ}}$	
4393.413	8	22754.96	−0.04	*b* ^4^D_3½_—*y* ${\;}^{4}D_{2½}^{{}^{\circ}}$	
4408.083	5*s*	22679.25	0.03	*a* ^2^I_5½_—*z* ${\;}^{2}I_{5½}^{{}^{\circ}}$	6
4410.493	2	22666.86	−0.04	*b* ^4^P_0½_—*y* ${\;}^{4}S_{1½}^{{}^{\circ}}$	
4411.874	10	22659.77	−0.02	*a* ^2^H_5½_—*z* ${\;}^{2}G_{4½}^{{}^{\circ}}$	4
4414.887	40	22644.30	0.04	*a* ^4^D_3½_—*z* ${\;}^{4}D_{2½}^{{}^{\circ}}$	5
4419.769	10	22619.29	0.05	*a* ^2^H_4½_—*z* ${\;}^{2}G_{3½}^{{}^{\circ}}$	4
4433.720	1*s*	22548.12	−0.19	*a* ^2^H_4½_—*z* ${\;}^{2}G_{4½}^{{}^{\circ}}$	
4434.139	2	22545.98	0.32	*a* ^2^F_2½_—*w* ${\;}^{4}G_{3½}^{{}^{\circ}}$	
4436.061	6*h*	22536.22	−0.07	*a* ^2^F_2½_—*w* ${\;}^{4}G_{2½}^{{}^{\circ}}$	
4436.358	80	22534.70	0.01	*a* ^4^D_2½_—*z* ${\;}^{4}D_{1½}^{{}^{\circ}}$	5
4439.873	0	22516.87	−0.14	*a* ^2^G_4½_—*z* ${\;}^{2}H_{4½}^{{}^{\circ}}$	
4451.575	100	22457.66	0.06	*a* ^4^D_3½_—*z* ${\;}^{4}D_{3½}^{{}^{\circ}}$	6, 7*b*
4452.525	7*s*	22452.88	0.01	*a* ^2^I_6½_—*z* ${\;}^{2}I_{6½}^{{}^{\circ}}$	
4453.013	50	22450.42	0.01	*a* ^4^D_1½_—*z* ${\;}^{4}D_{0½}^{{}^{\circ}}$	5
4455.019	25	22440.32	0.00	*z* ${\;}^{6}P_{1½}^{{}^{\circ}}$—*e* ^6^D_0½_	
4455.320	25	22438.80	−0.03	*z* ${\;}^{6}P_{1½}^{{}^{\circ}}$—*e* ^6^D_1½_	
4455.820	25	22436.29	0.00	*z* ${\;}^{6}P_{1½}^{{}^{\circ}}$—*e* ^6^D_2½_	
4457.041	20	22430.14	0.04	*z* ${\;}^{6}P_{2½}^{{}^{\circ}}$—*e* ^6^D_1½_	
4457.553	20	22427.56	0.00	*z* ${\;}^{6}P_{2½}^{{}^{\circ}}$—*e* ^6^D_2½_	
4458.263	25	22423.99	−0.02	*z* ${\;}^{6}P_{2½}^{{}^{\circ}}$—*e* ^6^D_3½_	
4460.376	20	22413.37	0.01	*z* ${\;}^{6}P_{3½}^{{}^{\circ}}$—*e* ^6^D_2½_	
4461.089	30	22409.84	0.03	*z* ${\;}^{6}P_{3½}^{{}^{\circ}}$—*e* ^6^D_3½_	
4462.033	150	22405.05	0.02	*z* ${\;}^{6}P_{3½}^{{}^{\circ}}$—*e* ^6^D_4½_	
4464.679	80	22391.76	0.03	*a* ^4^D_2½_—*z* ${\;}^{4}D_{2½}^{{}^{\circ}}$	6, 7*b*
4470.142	60	22364.40	0.03	*a* ^4^D_1½_—*z* ${\;}^{4}D_{1½}^{{}^{\circ}}$	6, 7*b*
4472.793	100	22351.14	0.08	*a* ^4^D_0½_—*z* ${\;}^{4}D_{0½}^{{}^{\circ}}$	6, 0
4473.52	2	22347.2	0.1	*a* ^4^F_1½_—*y* ${\;}^{4}S_{1½}^{{}^{\circ}}$	
4479.399	10s	22318.18	−0.01	*a* ^2^G_3½_—*z* ${\;}^{2}H_{4½}^{{}^{\circ}}$	5
4489.23	2	22269.3	−0.1	*a* ^2^F_3½_—*w* ${\;}^{4}F_{4½}^{{}^{\circ}}$	
4490.078	30	22265.10	0.08	*a* ^4^D_0½_—*z* ${\;}^{4}D_{1½}^{{}^{\circ}}$	5
4491.652	10*s*	22257.30	$\{\begin{array}{r} \begin{array}{r} 0.00\\ 0.00 \end{array} \end{array}$	*a* ^2^G_4½_—*z* ${\;}^{2}H_{5½}^{{}^{\circ}}$ *a* ^2^G_4½_—*x* ${\;}^{2}F_{3½}^{{}^{\circ}}$	5
4496.638	8*s*	22232.62	−0.04	*b* ^4^G_5½_—*x* ${\;}^{4}G_{5½}^{{}^{\circ}}$	6
4498.897	20	22221.45	0.04	*a* ^4^D_1½_—*z* ${\;}^{4}D_{2½}^{{}^{\circ}}$	5
4502.223	30	22205.04	−0.03	*a* ^4^D_2½_—*z* ${\;}^{4}D_{3½}^{{}^{\circ}}$	5
4503.868	15	22196.93	0.05	*b* ^4^G_5½_—*z* ${\;}^{2}I_{6½}^{{}^{\circ}}$	4
4512.659	2	22153.69	0.00	*b* ^4^G_4½_—*x* ${\;}^{4}G_{3½}^{{}^{\circ}}$	
4520.016	1	22117.63	0.02	*a* ^2^G_3½_—*y* ${\;}^{2}H_{4½}^{{}^{\circ}}$	
4523.399	3*s*	22101.09	−0.23	*b* ^4^G_4½_—*x* ${\;}^{4}G_{4½}^{{}^{\circ}}$	6, 7*b*
4529.804	3*s*	22069.84	0.02	*a* ^2^F_3½_—*z* ${\;}^{2}G_{3½}^{{}^{\circ}}$	6
4534.481	2.5*s*	22047.08	−0.01	*b* ^4^G_3½_—*x* ${\;}^{4}G_{3½}^{{}^{\circ}}$	6, 7*b*
4538.475	2	22027.68	−0.09	*b* ^4^G_2½_—*x* ${\;}^{4}G_{2½}^{{}^{\circ}}$	
4541.26	2	22014.2	−0.1	*a* ^2^P_1½_—*w* ${\;}^{4}D_{2½}^{{}^{\circ}}$	
4544.423	5*s*	21998.85	−0.04	*a* ^2^F_3½_—*z* ${\;}^{2}G_{4½}^{{}^{\circ}}$	4
4546.32	12	21994.5	$\{\begin{array}{r} \begin{array}{r} 0.1\\ -0.2 \end{array} \end{array}$	*b* ^4^G_2½_—*x* ${\;}^{4}G_{3½}^{{}^{\circ}}$ *b* ^4^G_3½_—*x* ${\;}^{4}G_{4½}^{{}^{\circ}}$	
4561.13	2	21918.2	0.2	*z* ${\;}^{6}P_{2½}^{{}^{\circ}}$—*e* ^8^D_1½_	
4563.62	2	21906.4	0.3	*z* ${\;}^{6}P_{3½}^{{}^{\circ}}$—*e* ^8^D_3½_	
4564.0	2	21904.5	−0.3	*z* ${\;}^{6}P_{3½}^{{}^{\circ}}$—*e* ^8^D_2½_	
4565.77	10	21896.0	0.0	*y* ${\;}^{6}P_{2½}^{{}^{\circ}}$—*f* ^4^D_1½_	
4578.077	1	21837.13	−0.07	*b* ^4^G_5½_—*x* ${\;}^{4}F_{4½}^{{}^{\circ}}$	
4581.854	2	21819.13	−0.05	*a* ^4^H_5½_—*z* ${\;}^{2}I_{5½}^{{}^{\circ}}$	
4584.918	1	21804.56	0.08	*a* ^2^F_2½_—*z* ${\;}^{2}G_{3½}^{{}^{\circ}}$	
4586.114	(30*h*)	21798.04	−0.38	*a* ^2^P_1½_—*u* ${\;}^{4}P_{1½}^{{}^{\circ}}$	
4598.940	1	21738.08	0.12	*b* ^4^G_2½_—*v* ${\;}^{4}D_{1½}^{{}^{\circ}}$?	
4605.365	20	21707.75	0.05	*a* ^2^H_4½_—*z* ${\;}^{2}I_{5½}^{{}^{\circ}}$	5
4606.38	1	21702.9	0.0	*b* ^4^D_1½_—*u* ${\;}^{6}P_{2½}^{{}^{\circ}}$	
4607.61	7*s*	21697.2	0.0	*a* ^4^D_3½_—*x* ${\;}^{6}P_{3½}^{{}^{\circ}}$	
4614.12	1	21666.6	0.0	*b* ^4^P_2½_—*v* ${\;}^{6}F_{3½}^{{}^{\circ}}$?	
4615.65	8	21659.4	−0.2	*b* ^4^G_4½_—*x* ${\;}^{4}F_{3½}^{{}^{\circ}}$	
4620.21	5*h*	21638.0	0.0	*a* ^2^P_0½_—*w* ${\;}^{4}D_{1½}^{{}^{\circ}}$	
4622.74	8	21626.2	0.0	*b* ^4^G_2½_—*x* ${\;}^{4}F_{1½}^{{}^{\circ}}$	
4623.33	8	21623.4	−0.1	*b* ^4^G_3½_—*x* ${\;}^{4}F_{2½}^{{}^{\circ}}$	
4624.220	8	21619.22	0.29	*y* ${\;}^{6}P_{3½}^{{}^{\circ}}$—*e* ^8^P_4½_	
4626.544	25	21608.37	−0.05	*a* ^2^H_5½_—*z* ${\;}^{2}I_{6½}^{{}^{\circ}}$	4
4626.861	2	21606.89	−0.02	*a* ^4^D_2½_—*x* ${\;}^{6}P_{2½}^{{}^{\circ}}$	
4642.803	5*s*	21532.70	−0.02	*a* ^2^H_4½_—*x* ${\;}^{4}G_{5½}^{{}^{\circ}}$	
4658.42	12	21460.5	0.1	*b* ^4^P_1½_— $55923_{2½}^{{}^{\circ}}$	
4661.93	2	21444.4	−0.3	*a* ^4^D_2½_—*x* ${\;}^{6}P_{3½}^{{}^{\circ}}$	
4663.638	2	21436.51	−0.08	*a* ^4^D_1½_—*x* ${\;}^{6}P_{2½}^{{}^{\circ}}$	
4671.688	10*s*	21399.57	−0.05	*a* ^4^D_3½_—*z* ${\;}^{4}F_{2½}^{{}^{\circ}}$	5
4697.52	2	21281.9	0.1	*b* ^4^P_2½_—*x* ${\;}^{4}D_{3½}^{{}^{\circ}}$	
4701.150	8*s*	21265.46	−0.07	*a* ^4^D_2½_—*z* ${\;}^{4}F_{1½}^{{}^{\circ}}$	
4709.710	40	21226.81	0.03	*a* ^4^D_3½_—*z* ${\;}^{4}F_{3½}^{{}^{\circ}}$	6
4712.02	5	21216.5	0.0	*a* ^4^P_0½_—*u* ${\;}^{4}P_{0½}^{{}^{\circ}}$	
4727.462	30	21147.10	0.01	*a* ^4^D_2½_—*z* ${\;}^{4}F_{2½}^{{}^{\circ}}$	6
4728.789	1	21141.17	0.13	*b* ^4^D_0½_—*x* ${\;}^{4}P_{0½}^{{}^{\circ}}$	
4731.92	1	21127.2	0.1	*b* ^4^D_1½_—*x* ${\;}^{4}P_{0½}^{{}^{\circ}}$	
4739.110	25	21095.13	−0.08	*a* ^4^D_1½_—*z* ${\;}^{4}F_{1½}^{{}^{\circ}}$	6
4754.048	50H	21028.85	0.00	*z* ${\;}^{8}P_{2½}^{{}^{\circ}}$—*e* ^8^S_3½_	4
4761.527	50	20995.81	−0.05	*a* ^4^D_0½_—*z* ${\;}^{4}F_{1½}^{{}^{\circ}}$	5
4762.376	80	20992.09	0.00	*a* ^4^D_3½_—*z* ${\;}^{4}F_{4½}^{{}^{\circ}}$	4
4765.856	60	20976.71	−0.06	*a* ^4^D_1½_—*z* ${\;}^{4}F_{2½}^{{}^{\circ}}$	4
4766.426	70	20974.25	0.00	*a* ^4^D_2½_—*z* ${\;}^{4}F_{3½}^{{}^{\circ}}$	4
4771.668	4*s*	20951.18	−0.02	*b* ^4^D_3½_—*x* ${\;}^{4}P_{2½}^{{}^{\circ}}$	
4779.138	4	20918.44	0.01	*b* ^4^G_5½_—*y* ${\;}^{4}H_{6½}^{{}^{\circ}}$	
4783.432	100	20899.67	0.00	*z* ${\;}^{8}P_{3½}^{{}^{\circ}}$—*e* ^8^S_3½_	6
4786.867	3	20884.67	−0.01	*z* ${\;}^{6}D_{0½}^{{}^{\circ}}$—*e* ^6^F_1½_	
4793.011	8	20857.90	−0.06	*a* ^2^H_5½_—*z* ${\;}^{4}I_{4½}^{{}^{\circ}}$	
4796.055	3	20844.62	−0.10	*a* ^2^G_3½_—*y* ${\;}^{2}F_{2½}^{{}^{\circ}}$	
4796.704	15	20841.85	−0.23	*y* ${\;}^{6}P_{2½}^{{}^{\circ}}$—*i* ^6^D_1½_	
4798.324	1	20834.81	0.12	*a* ^2^H_5½_—*z* ${\;}^{4}I_{6½}^{{}^{\circ}}$	
4807.170	3*s*	20796.46	−0.22	*b* ^4^G_4½_—*y* ${\;}^{4}H_{5½}^{{}^{\circ}}$	
4815.109	15*h*	20762.18	−0.06	*z* ${\;}^{6}D_{1½}^{{}^{\circ}}$—*e* ^6^F_2½_	
4816.909	1	20754.42	−0.04	*a* ^2^G_4½_—*y* ${\;}^{2}G_{3½}^{{}^{\circ}}$	
4818.331	2*s*	20748.29	−0.01	*b* ^4^G_3½_—*y* ${\;}^{4}H_{4½}^{{}^{\circ}}$	
4822.708	10*h*	20729.46	−0.51	*b* ^4^G_2½_—*y* ${\;}^{4}H_{3½}^{{}^{\circ}}$?	
4823.528	150	20725.94	0.00	*z* ${\;}^{8}P_{4½}^{{}^{\circ}}$—*e* ^8^S_3½_	4
4825.592	20	20717.07	0.00	*z* ${\;}^{4}P_{2½}^{{}^{\circ}}$—*e* ^4^P_1½_	4
4826.890	10	20711.50	0.00	*z* ${\;}^{4}P_{1½}^{{}^{\circ}}$—*e* ^4^P_0½_	4
4838.228	8	20662.97	0.00	*z* ${\;}^{4}P_{0½}^{{}^{\circ}}$—*e* ^4^P_0½_	
4840.139	3	20654.81	−0.01	*b* ^4^G_5½_—*y* ${\;}^{4}G_{5½}^{{}^{\circ}}$	
4840.21	2	20654.51	0.02	*b* ^4^P_0__½_—*y* ${\;}^{8}F_{1½}^{{}^{\circ}}$	
4843.188	4	20641.81	0.01	*z* ${\;}^{4}P_{1½}^{{}^{\circ}}$—*e* ^4^P_1½_	
4844.312	20*s*	20637.03	0.01	*z* ${\;}^{4}P_{2½}^{{}^{\circ}}$—*e* ^4^P_2½_	6
4854.610	10*s*	20593.24	−0.03	*z* ${\;}^{4}P_{0½}^{{}^{\circ}}$—*e* ^4^P_1½_	4
4862.048	15*s*	20561.74	−0.01	*z* ${\;}^{4}P_{1½}^{{}^{\circ}}$—*e* ^4^P_2½_	4
4881.578	3	20479.47	−0.15	*b* ^4^G_4½_—*y* ${\;}^{4}G_{4½}^{{}^{\circ}}$	
4888.833	2	20449.08	−0.04	*a* ^2^G_4½_—*w* ${\;}^{4}G_{3½}^{{}^{\circ}}$	
4900.719	2	20399.47	0.00	*b* ^4^G_3½_—*y* ${\;}^{4}G_{3½}^{{}^{\circ}}$	
4902.202	1	20393.32	0.10	*b* ^4^P_2½_—*z* ${\;}^{4}S_{1½}^{{}^{\circ}}$	
4907.868	2	20369.77	−0.03	*b* ^4^G_2½_—*y* ${\;}^{4}G_{2½}^{{}^{\circ}}$	
4917.446	1	20330.10	0.13	*a* ^2^H_5½_—*y* ${\;}^{4}H_{6½}^{{}^{\circ}}$	
4939.115	6*h*	20240.91	−0.02	*z* ${\;}^{6}D_{2½}^{{}^{\circ}}$—*e* ^6^F_3½_	
4942.396	3	20227.47	0.06	*a* ^4^D_3½_—*z* ${\;}^{6}F_{3½}^{{}^{\circ}}$	
4956.733	1*s*	20168.97	0.15	*a* ^4^F_4½_—*x* ${\;}^{4}D_{3½}^{{}^{\circ}}$	
4965.856	30	20131.91	0.00	*a* ^4^D_3½_—*z* ${\;}^{6}F_{4½}^{{}^{\circ}}$	5
4974.334	6*h*	20097.60	0.06	*z* ${\;}^{6}D_{3½}^{{}^{\circ}}$—*e* ^6^F_4½_	
4982.067	1	20066.41	0.05	*a* ^2^H_5½_—*y* ${\;}^{4}G_{5½}^{{}^{\circ}}$	
4985.734	10*s*	20051.65	0.36	*a* ^4^P_1½_—*y* ${\;}^{4}P_{0½}^{{}^{\circ}}$	
4987.066	2*s*	20046.29	−0.01	*a* ^4^D_2½_—*z* ${\;}^{6}F_{2½}^{{}^{\circ}}$	
4994.213	3*s*	20017.61	0.17	*a* ^4^P_0½_—*y* ${\;}^{4}P_{0½}^{{}^{\circ}}$	
5004.891	20*s*	19974.90	0.02	*a* ^4^D_2½_—*z* ${\;}^{6}F_{3½}^{{}^{\circ}}$	
5010.349	8*s*	19953.14	0.17	*a* ^4^P_2½_—*y* ${\;}^{4}P_{1½}^{{}^{\circ}}$	
5017.612	20*h*	19924.26	0.12	*z* ${\;}^{6}D_{4½}^{{}^{\circ}}$—*e* ^6^F_5½_	
5022.004	5*s*	19906.83	0.32	*a* ^4^P_1½_—*y* ${\;}^{4}P_{1½}^{{}^{\circ}}$	
5029.779	10*s*	19876.06	0.08	*a* ^4^D_1½_—*z* ${\;}^{6}F_{2½}^{{}^{\circ}}$	
5030.623	5*s*	19872.73	0.07	*a* ^4^P_0½_—*y* ${\;}^{4}P_{1½}^{{}^{\circ}}$	
5042.567	4*s*	19825.55	−0.03	*a* ^4^D_0½_—*z* ${\;}^{6}F_{1½}^{{}^{\circ}}$	
5074.805	15	19699.72	0.13	*a* ^4^P_2½_—*y* ${\;}^{4}P_{2½}^{{}^{\circ}}$	
5086.884	10*s*	19652.94	−0.19	*a* ^4^P_1½_—*y* ${\;}^{4}P_{2½}^{{}^{\circ}}$	
5091.018	1*s*	19636.98	−0.03	*a* ^2^G_4½_—*z* ${\;}^{2}G_{4½}^{{}^{\circ}}$	
5117.935	20*s*	19533.70	0.01	*a* ^4^G_2½_—*z* ${\;}^{4}F_{1½}^{{}^{\circ}}$	P–B
5142.919	15*s*	19438.81	0.02	*z* ${\;}^{6}F_{1½}^{{}^{\circ}}$—*e* ^6^F_1½_	
5149.155	50	19415.27	0.02	*a* ^4^G_2½_—*z* ${\;}^{4}F_{2½}^{{}^{\circ}}$	
5150.937	30*s*	19408.55	0.00	*a* ^4^G_3½_—*z* ${\;}^{4}F_{2½}^{{}^{\circ}}$	
5177.077	2	19310.55	0.24	*z* ${\;}^{6}F_{2½}^{{}^{\circ}}$—*e* ^6^F_2__½_	
5180.298	2*s*	19298.55	−0.05	*b* ^4^P_2__½_—*y* ${\;}^{4}D_{3½}^{{}^{\circ}}$	
5190.02	6*h*	19262.40	−0.64	*y* ${\;}^{6}P_{1½}^{{}^{\circ}}$—*g* ^6^D_1½_	
5190.69	6*h*	19259.91	−0.01	*y* ${\;}^{6}P_{1½}^{{}^{\circ}}$—*g* ^6^D_0½_	
5196.603	30*s*	19238.00	−0.02	*a* ^4^G_4½_—*z* ${\;}^{4}F_{3½}^{{}^{\circ}}$	
5197.229	10	19235.68	−0.03	*a* ^4^G_3½_—*z* ${\;}^{4}F_{3½}^{{}^{\circ}}$	
5199.51	3	19227.24	0.07	*y* ${\;}^{6}P_{2½}^{{}^{\circ}}$—*g* ^6^D_1½_	
5200.18	5	19224.77	−0.04	*y* ${\;}^{6}P_{2½}^{{}^{\circ}}$—*g* ^6^D_2½_	
5201.440	2H	19220.11	−0.13	*y* ${\;}^{6}P_{2½}^{{}^{\circ}}$—*g* ^6^D_3½_	
5213.34	8	19176.24	0.12	*y* ${\;}^{6}P_{3½}^{{}^{\circ}}$—*g* ^6^D_3½_	
5215.434	5H	19168.54	−0.05	*y* ${\;}^{6}P_{3½}^{{}^{\circ}}$—*g* ^6^D_4½_	
5249.371	2	19044.60	−0.01	*b* ^4^P_2½_—*x* ${\;}^{6}D_{3½}^{{}^{\circ}}$	
5255.330	30	19023.02	0.00	*a* ^4^G_5½_—*z* ${\;}^{4}F_{4½}^{{}^{\circ}}$	4
5260.771	8*s*	19003.34	0.01	*a* ^4^G_4½_—*z* ${\;}^{4}F_{4½}^{{}^{\circ}}$	
5274.206	2	18954.94	−0.02	*z* ${\;}^{6}F_{3½}^{{}^{\circ}}$—*e* ^4^G_4½_	
5277.54	8H	18942.96	0.29	*z* ${\;}^{4}F_{4½}^{{}^{\circ}}$—*e* ^4^F_4½_	
5284.196	6H	18919.10	0.01	*z* ${\;}^{6}F_{2½}^{{}^{\circ}}$—*e* ^6^G_2½_?	
5289.495	5*h*	18900.15	$\{\begin{array}{r} \begin{array}{r} -0.30\\ -0.40 \end{array} \end{array}$	*z* ${\;}^{6}F_{0½}^{{}^{\circ}}$—*e* ^6^G_1½_ *z* ${\;}^{4}F_{3½}^{{}^{\circ}}$—*e* ^4^F_3½_	
5292.869	1	18888.10	0.02	*a* ^4^P_0½_—*z* ${\;}^{4}D_{0½}^{{}^{\circ}}$	
5297.920	3*hd*	18870.10	−0.04	*z* ${\;}^{6}F_{1½}^{{}^{\circ}}$—*e* ^6^G_2½_	
5298.84	7*s*	18866.82	0.04	*z* ${\;}^{6}F_{4½}^{{}^{\circ}}$—*e* ^4^G_5½_	
5308.92	15*h*	18831.00	0.02	*z* ${\;}^{6}F_{2½}^{{}^{\circ}}$—*e* ^6^G_3½_	
5317.082	8*s*	18802.09	0.05	*a* ^4^P_0½_—*z* ${\;}^{4}D_{1½}^{{}^{\circ}}$	
5324.317	20*h*	18776.54	−0.01	*z* ${\;}^{6}F_{3½}^{{}^{\circ}}$—*e* ^6^G_4½_	
5325.999	5*h*	18770.61	0.03	*z* ${\;}^{6}F_{3½}^{{}^{\circ}}$—*e* ^6^F_3½_	
5334.872	5*s*	18739.39	0.00	*a* ^4^P_2½_—*z* ${\;}^{4}D_{2½}^{{}^{\circ}}$	
5341.065	250	18717.66	−0.02	*a* ^6^D_4½_—*y* ${\;}^{6}P_{3½}^{{}^{\circ}}$	4
5344.438	50H	18705.85	−0.02	*z* ${\;}^{6}F_{4½}^{{}^{\circ}}$—*e* ${\;}^{6}G_{5½}^{{}^{\circ}}$	
5348.078	20*s*	18693.12	0.19	*a* ^4^P_1½_—*z* ${\;}^{4}D_{2½}^{{}^{\circ}}$	
5349.878	80H	18686.83	−0.03	*z* ${\;}^{6}F_{5½}^{{}^{\circ}}$—*e* ^6^G_6½_	
5361.631	1	18645.86	0.02	*b* ^4^P_1½_—*y* ${\;}^{4}D_{2½}^{{}^{\circ}}$	
5364.488	5	18635.94	−0.03	*a* ^4^D_3½_—*z* ${\;}^{6}D_{3½}^{{}^{\circ}}$	
5374.39	1*h*	18601.60	0.00	*z* ${\;}^{6}F_{4½}^{{}^{\circ}}$—*e* ^6^F_4½_	
5375.306	2	18598.42	0.00	*z* ${\;}^{4}H_{6½}^{{}^{\circ}}$—*f* ^4^G_5½_?	
5377.634	100	18590.38	0.02	*z* ${\;}^{4}P_{2½}^{{}^{\circ}}$—*e* ^4^S_1½_	4
5388.538	15*s*	18552.80	0.07	*a* ^4^P_2½_—*z* ${\;}^{4}D_{3½}^{{}^{\circ}}$	
5394.677	30	18531.65	0.01	*a* ^6^S_2½_—*z* ${\;}^{8}P_{3½}^{{}^{\circ}}$	4
5399.506	30	18515.08	−0.01	*z* ^4^P_1½_—*e* ${\;}^{4}S_{1½}^{{}^{\circ}}$	6
5402.608	1	18504.45	−0.08	*a* ^4^D_2½_—*z* ${\;}^{6}D_{2½}^{{}^{\circ}}$	
5406.014	5	18492.79	−0.02	*a* ^4^D_3½_—*z* ${\;}^{6}D_{4½}^{{}^{\circ}}$	
5407.432	50*c*	18487.97	0.00	*a* ^6^D_3½_—*y* ${\;}^{6}P_{3½}^{{}^{\circ}}$	6
5413.696	30	18466.55	−0.01	*z* ${\;}^{4}P_{0½}^{{}^{\circ}}$—*e* ^4^S_1½_	4
5420.368	100*c*	18443.82	−0.03	*a* ^6^D_3½_—*y* ${\;}^{6}P_{2½}^{{}^{\circ}}$	4
5426.192	2	18424.03	−0.02	*a* ^4^D_1½_—*z* ${\;}^{6}D_{1½}^{{}^{\circ}}$	
5427.184	1*h*	18420.66	0.16	*b* ^4^P_1½_—*x* ${\;}^{6}D_{1½}^{{}^{\circ}}$, _2½_	
5432.555	60	18402.45	−0.01	*a* ^6^S_2½_—*z* ${\;}^{8}P_{2½}^{{}^{\circ}}$	
5433.422	100*wc*	18399.50	0.11	*z* ${\;}^{6}F_{5½}^{{}^{\circ}}$—*e* ^6^F_5½_	
5439.290	0	18379.66	−0.03	*a* ^4^D_0½_—*z* ${\;}^{6}D_{0½}^{{}^{\circ}}$	
5444.094	1*h*	18363.44	0.03	*a* ^4^G_2½_—*z* ${\;}^{6}F_{1½}^{{}^{\circ}}$	
5445.99	1*h*	18357.04	0.10	*y* ${\;}^{4}F_{3½}^{{}^{\circ}}$—*f* ^4^G_4½_	
5447.576	1*h*	18351.70	−0.02	*y* ${\;}^{4}F_{4½}^{{}^{\circ}}$—*f* ^4^G_5½_	
5457.468	30	18318.44	−0.01	*a* ^6^D_2½_—*y* ${\;}^{6}P_{3½}^{{}^{\circ}}$	
5460.645	1	18307.78	0.02	*a* ^4^G_3½_—*z* ${\;}^{6}F_{2½}^{{}^{\circ}}$	
5470.640	100	18274.33	0.00	*a* ^6^D_2½_—*y* ${\;}^{6}P_{2½}^{{}^{\circ}}$	6
5481.345	100	18238.48	0.02	*a* ^6^D_2½_—*y* ${\;}^{6}P_{1½}^{{}^{\circ}}$	
5495.913	1	18190.30	0.02	*z* ${\;}^{4}F_{4½}^{{}^{\circ}}$—*e* ^4^G_4½_	
5497.374	10*h*	18185.47	0.08	*a* ^4^F_4½_—*y* ${\;}^{4}D_{3½}^{{}^{\circ}}$	
5504.224	15	18162.83	−0.01	*a* ^4^G_5½_—*z* ${\;}^{6}F_{4½}^{{}^{\circ}}$	
5505.877	50	18157.38	0.01	*a* ^6^D_1½_—*y* ${\;}^{6}P_{2½}^{{}^{\circ}}$	
5510.190	1	18143.16	0.01	*a* ^4^G_4½_—*z* ${\;}^{6}F_{4½}^{{}^{\circ}}$	
5516.777	100	18121.50	0.00	*a* ^6^D_1½_—*y* ${\;}^{6}P_{1½}^{{}^{\circ}}$	6
5520.496	10*h*	18109.29	−0.03	*z* ${\;}^{4}F_{3½}^{{}^{\circ}}$—*e* ^4^G_3½_	
5533.156	5*s*	18067.86	0.02	*a* ^4^F_3½_—*y* ${\;}^{4}D_{2½}^{{}^{\circ}}$	
5536.471	8*h*	18057.04	−0.04	*z* ${\;}^{4}F_{2½}^{{}^{\circ}}$—*e* ^4^G_2½_	
5537.749	100	18052.88	0.05	*a* ^6^D_0½_—*y* ${\;}^{6}P_{1½}^{{}^{\circ}}$	4
5551.978	80*w*	18006.60	0.00	*z* ${\;}^{4}F_{4½}^{{}^{\circ}}$—*e* ^4^G_5½_	
5555.782	3*s*	17994.29	0.06	*a* ^4^F_2½_—*y* ${\;}^{4}D_{2½}^{{}^{\circ}}$	
5567.764	50	17955.55	−0.04	*z* ${\;}^{4}F_{3½}^{{}^{\circ}}$—*e* ^4^G_4½_	
5573.010	30	17938.67	0.03	*z* ${\;}^{4}F_{1½}^{{}^{\circ}}$—*e* ^4^G_2½_	
5573.690	40	17936.48	0.00	*z* ${\;}^{4}F_{2½}^{{}^{\circ}}$—*e* ^4^G_3½_	
5575.242	1	17931.46	0.06	*a* ^4^F_4½_—*x* ${\;}^{6}D_{3½}^{{}^{\circ}}$	
5602.046	5	17845.68	−0.01	*z* ${\;}^{4}F_{4½}^{{}^{\circ}}$—*e* ^6^G_5½_	
5603.08	1	17842.38	−0.12	*a* ^4^F_3½_—*x* ${\;}^{6}D_{2½}^{{}^{\circ}}$	
5607.355	1	17828.78	0.05	*a* ^4^F_3½_—*x* ${\;}^{6}D_{3½}^{{}^{\circ}}$	
5626.247	3	17768.93	0.04	*a* ^4^F_2½_—*x* ${\;}^{6}D_{1½,\;2½}^{{}^{\circ}}$	
5642.394	1	17718.07	0.02	*a* ^4^F_1½_—*x* ${\;}^{6}D_{0½}^{{}^{\circ}}$	
5658.395	1	17667.97	0.05	*b* ^4^G_3½_—*w* ${\;}^{4}P_{2½}^{{}^{\circ}}$	
5687.133	1	17578.68	−0.05	*b* ^4^G_2½_—*w* ${\;}^{4}P_{1½}^{{}^{\circ}}$	
5718.236	2*h*	17483.07	0.00	*z* ${\;}^{4}D_{2½}^{{}^{\circ}}$—*e* ^4^F_3½_	
5720.233	2*h*	17476.97	−0.19	*z* ${\;}^{4}D_{3½}^{{}^{\circ}}$—*e* ^4^F_4½_	
5738.286	30	17422.00	−0.05	*a* ^4^H_6½_—*z* ${\;}^{4}G_{5½}^{{}^{\circ}}$	
5780.173	25	17295.74	−0.01	*a* ^4^H_5½_—*z* ${\;}^{4}G_{4½}^{{}^{\circ}}$	
5782.307	1*h*	17289.34	−0.26	*a* ^2^G_3½_—*y* ${\;}^{4}H_{3½}^{{}^{\circ}}$	
5816.840	20	17186.71	0.00	*a* ^4^H_4½_—*z* ${\;}^{4}G_{3½}^{{}^{\circ}}$	
5822.55	3	17169.86	−0.09	*z* ${\;}^{4}G_{4½}^{{}^{\circ}}$—*f* ^4^G_4½_	
5835.372	5	17132.12	0.03	*z* ${\;}^{4}G_{5½}^{{}^{\circ}}$—*f* ^4^G_5½_	
5848.950	15	17092.36	0.00	*a* ^4^H_3½_—*z* ${\;}^{4}G_{2½}^{{}^{\circ}}$	
5868.18	2	17036.35	0.09	*x* ${\;}^{6}P_{3½}^{{}^{\circ}}$—*e* ^6^F_4½_	
5879.18	2	17004.47	−0.09	*y* ${\;}^{6}P_{2½}^{{}^{\circ}}$—*f* ^6^D_3½_	
5973.867	2	16734.95	0.05	*b* ^4^D_2½_—*y* ${\;}^{4}P_{1½}^{{}^{\circ}}$	
6013.484	100	16624.70	0.09	*z* ${\;}^{6}P_{1½}^{{}^{\circ}}$—*e* ^6^S_2½_	4
6014.385	20*s*	16622.21	−0.02	*a* ^4^F_4½_—*z* ${\;}^{4}G_{5½}^{{}^{\circ}}$	
6016.697	150	16615.82	−0.06	*z* ${\;}^{6}P_{2½}^{{}^{\circ}}$—*e* ^6^S_2½_	6
6021.787	200	16601.78	0.10	*z* ${\;}^{6}P_{3½}^{{}^{\circ}}$—*e* S_2½_	4
6041.730	8*s*	16546.98	0.06	*b* ^4^D_3½_—*y* ${\;}^{4}P_{2½}^{{}^{\circ}}$	
6057.120	15*s*	16504.94	0.04	*a* ^4^F_3½_—*z* ${\;}^{4}G_{4½}^{{}^{\circ}}$	
6062.885	3*s*	16489.24	0.00	*a* ^4^F_3½_—*z* ${\;}^{4}G_{3½}^{{}^{\circ}}$	
6090.080	10*s*	16415.61	−0.02	*a* ^4^F_2½_—*z* ${\;}^{4}G_{3½}^{{}^{\circ}}$	
6095-656	2*s*	16400.60	−0.05	*a* ^4^F_2½_—*z* ${\;}^{4}G_{2½}^{{}^{\circ}}$	
6114.324	8*s*	16350.52	−0.06	*a* ^4^F_1½_—*z* ${\;}^{4}G_{2½}^{{}^{\circ}}$	
6177.5	2	16183.31	−0.08	*e* ^6^D_3½_—*w* ${\;}^{4}H_{3½}^{{}^{\circ}}$	
6265.627	10*s*	15955.69	−0.03	*a* ^4^H_6½_—*z* ${\;}^{4}H_{6½}^{{}^{\circ}}$	
6315.064	10*s*	15830.78	−0.01	*a* ^4^H_5½_—*z* ${\;}^{4}H_{5½}^{{}^{\circ}}$	
6344.133	30	15758.23	0.04	*b* ^4^D_0½_—*z* ${\;}^{4}D_{0½}^{{}^{\circ}}$	
6349.795	15	15744.19	−0.03	*b* ^4^D_1½_—*z* ${\;}^{4}D_{0½}^{{}^{\circ}}$	
6356.095	8*s*	15728.59	−0.10	*a* ^4^H_4½_—*z* ${\;}^{4}H_{4½}^{{}^{\circ}}$	
6378.969	20	15072.19	0.04	*b* ^4^D_0½_—*z* ${\;}^{4}D_{1½}^{{}^{\circ}}$	
6382.195	25	15664.27	−0.01	*b* ^4^D_2½_—*z* ${\;}^{4}D_{1½}^{{}^{\circ}}$	
6384.686	30	15658.16	−0.02	*b* ^4^D_1½_—*z* ${\;}^{4}D_{1½}^{{}^{\circ}}$	
6391.248	7*s*	15642.08	−0.11	*a* ^4^H_3½_—*z* ${\;}^{4}H_{3½}^{{}^{\circ}}$	
6413.947	2	15580.72	0.00	*b* ^4^D_3½_—*z* ${\;}^{4}D_{2½}^{{}^{\circ}}$	
6440.973	50	15521.32	0.00	*b* ^4^D_2½_—*z* ${\;}^{4}D_{2½}^{{}^{\circ}}$	
6443.511	20	15515.21	−0.01	*b* ^4^D_1½_—*z* ${\;}^{4}D_{2½}^{{}^{\circ}}$	
6483.061	2*s*	15420.56	−0.02	*a* ^4^F_4½_—*y* ${\;}^{4}F_{3½}^{{}^{\circ}}$	
6590.61	3	15402.62	0.02	*a* ^4^F_4½_—*y* ${\;}^{4}F_{4½}^{{}^{\circ}}$	
6491.697	50	15400.04	−0 02	*b* ^4^D_3½_—*z* ${\;}^{4}D_{3½}^{{}^{\circ}}$	
6519.381	20	15334.65	−0.01	*b* ^4^D_2½_—*z* ${\;}^{4}D_{3½}^{{}^{\circ}}$	
6520.548	2*h*	15331.90	0.04	*a* ^4^F_3½_—*y* ${\;}^{4}F_{3½}^{{}^{\circ}}$	
6526.528	8	15317.86	−0.05	*a* ^4^F_3½_—*y* ${\;}^{4}F_{3½}^{{}^{\circ}}$	
6534.103	6HH	15300.10	0.17	*a* ^4^F_3½_—*y* ${\;}^{4}F_{4½}^{{}^{\circ}}$	
6547.715	2*h*	15268.29	0.00	*a* ^4^F_2½_—*y* ${\;}^{4}F_{1½}^{{}^{\circ}}$	
6552.035	5	15258.23	−0.02	*a* ^4^F_2½_—*y* ${\;}^{4}F_{2½}^{{}^{\circ}}$	
6558.016	2*h*	15244.31	0.01	*a* ^4^F_2½_—*y* ${\;}^{4}F_{3½}^{{}^{\circ}}$	
6569.267	5	15218.20	−0.02	*a* ^4^F_1½_—*y* ${\;}^{4}F_{1½}^{{}^{\circ}}$	
6570.807	7HH	15214.64	−0.06	*y* ${\;}^{6}P_{1½}^{{}^{\circ}}$—*g* ^6^S_2__½_	
6572.936	2*s*	15209.71	−0.20	*y* ${\;}^{6}F_{5½}^{{}^{\circ}}$—*e* ^4^F_4½_?	
6573.589	1*h*	15208.20	0.02	*a* ^4^F_1½_—*y* ${\;}^{4}F_{2½}^{{}^{\circ}}$	
6586.316	8HH	15178.81	−0.02	*y* ${\;}^{6}P_{2½}^{{}^{\circ}}$—*g* ^6^S_2½_	
6605.549	10HH	15134.62	−0.09	*y* ${\;}^{6}P_{3½}^{{}^{\circ}}$—*g* ^6^S_2½_	
6827.161	3*h*	14643.34	0.08	*z* ${\;}^{6}P_{2½}^{{}^{\circ}}$—*e* ^8^S_3½_	
6833.793	5*h*	14629.13	−0.18	*z* ${\;}^{6}D_{3½}^{{}^{\circ}}$—*e* ^4^D_2½_	
6833.936	10*h*	14628.83	−0.23	*z* ${\;}^{6}P_{3½}^{{}^{\circ}}$—*e* ^8^S_3½_	
6863.08	4H	14566.71	−0.02	*z* ${\;}^{6}D_{4½}^{{}^{\circ}}$—*i* ^6^D_3½_	
6867.18	3HH	14558.01	−0.14	*z* ${\;}^{6}D_{3½}^{{}^{\circ}}$—*i* ^6^D_2½_	
6880.64	5H	14529.53	0.09	*z* ${\;}^{6}D_{3½}^{{}^{\circ}}$—*e* ^4^D_3½_	
6887.73	15*s*	14514.57	0.37	*z* ${\;}^{6}D_{2½}^{{}^{\circ}}$—*i* ^6^D^1^_½_	
6890.764	1HHH	14508.18	−0.04	*z* ${\;}^{6}D_{2½}^{{}^{\circ}}$—*e* ^4^D_2½_	
6924.690	3H	14437.10	0.04	*z* ${\;}^{6}D_{2½}^{{}^{\circ}}$—*i* ^6^D_2½_	
6931.165	10H	14423.62	0.05	*z* ${\;}^{6}D_{3½}^{{}^{\circ}}$—*i* ^6^D_3½_	
6933.654	3HH	14418.44	0.06	*z* ${\;}^{6}D_{1½}^{{}^{\circ}}$—*e* ^4^D_2½_	
6938.486	4H	14408.40	0.05	*z* ${\;}^{6}D_{2½}^{{}^{\circ}}$—*e* ^4^D_3½_	
6941.045	2H	14403.09	$\{\begin{array}{r} \begin{array}{r} 0.10\\ 0.02 \end{array} \end{array}$	*b* ^4^D_0½_—*z* ${\;}^{4}F_{1½}^{{}^{\circ}}$ *z* ${\;}^{6}D_{0½}^{{}^{\circ}}$—*e* ^4^D_1½_	
6942.538	20H	14399.99	0.02	*z* ${\;}^{6}D_{4½}^{{}^{\circ}}$—*i* ^6^D_4__½_	
6947.828	1	14389.02	0.00	*b* ^4^D_1½_—*z* ${\;}^{4}F_{1½}^{{}^{\circ}}$	
6957.328	1*h*	14369.37	0.00	*z* ${\;}^{6}D_{0½}^{{}^{\circ}}$—*i* ^6^D_1½_	
6960.942	1*h*	14361.91	0.05	*y* ${\;}^{6}F_{2½}^{{}^{\circ}}$—*e* ^4^G_3½_	
6968.062	3HH	14347.24	0.02	*z* ${\;}^{6}D_{1½}^{{}^{\circ}}$—*i* ^6^D_2½_	
6970.567	1	14342.09	0.01	*b* ^4^D_3½_—*z* ${\;}^{4}F_{2½}^{{}^{\circ}}$	
6989.914	15*h*	14302.39	−0.09	*z* ${\;}^{6}D_{2½}^{{}^{\circ}}$—*i* ^6^D_3½_	
7002.50	4*s*	14276.68	0.00	*b* ^4^D_2½_—*z* ${\;}^{4}F_{2½}^{{}^{\circ}}$	
7005.500	2*s*	14270.57	−0.01	*b* ^4^D_1½_—*z* ${\;}^{4}F_{2½}^{{}^{\circ}}$	
7012.256	10H	14256.82	0.01	*z* ${\;}^{6}D_{3½}^{{}^{\circ}}$—*i* ^6^D_4½_	
7013.15	2H	14255.00	0.01	*y* ${\;}^{6}F_{0½}^{{}^{\circ}}$—*e* ^6^G_1½_	
7014.09	1H	14253.09	0.04	*y* ${\;}^{6}F_{3½}^{{}^{\circ}}$—*e* ^4^G_4½_	
7033.555	1HHH	14213.65	0.04	*y* ${\;}^{6}F_{1½}^{{}^{\circ}}$—*e* ^6^G_2½_	
7055.583	4*s*	14169.27	0.03	*b* ^4^D_3½_—*z* ${\;}^{4}F_{3½}^{{}^{\circ}}$	
7062.42	1HH	14155.55	−0.02	*y* ${\;}^{6}F_{2½}^{{}^{\circ}}$—*e* ^6^G_3½_	
7069.834	20*s*	14140.71	0.02	*b* ^4^G_5½_—*z* ${\;}^{4}G_{5½}^{{}^{\circ}}$	
7076.553	2H	14127.28	−0.07	*y* ${\;}^{6}F_{4½}^{{}^{\circ}}$—*e* ^4^G_3½_	
7077.145	1	14126.10	0.07	*b* ^4^G_5½_—*z* ${\;}^{4}G_{4½}^{{}^{\circ}}$	
7088.288	3*s*	14103.89	0.05	*b* ^4^D_2½_—*z* ${\;}^{4}F_{3½}^{{}^{\circ}}$	
7103.010	2HH	14074.66	0.02	*y* ${\;}^{6}F_{3½}^{{}^{\circ}}$—*e* ^6^G_4½_	
7151.267	5HH	13979.69	0.03	*y* ${\;}^{6}F_{5½}^{{}^{\circ}}$—*e* ^6^G_6½_	
7158.039	2HH	13966.46	0.02	*y* ${\;}^{6}F_{4½}^{{}^{\circ}}$—*e* ^6^G_5½_	
7174.420	4*s*	13934.57	0.02	*b* ^4^D_3½_—*z* ${\;}^{4}F_{4½}^{{}^{\circ}}$	
7176.705	2*h*	13930.14	−0.17	*b* ^4^G_4½_—*z* ${\;}^{4}G_{5½}^{{}^{\circ}}$	
7184.29	15*s*	13915.43	−0.22	*b* ^4^G_4½_—*z* ${\;}^{4}G_{4½}^{{}^{\circ}}$	
7192.363	1*h*	13899.81	−0.18	*b* ^4^G_4½_—*z* ${\;}^{4}G_{3½}^{{}^{\circ}}$	
7211.93	2	13862.17	0.00	*y* ${\;}^{6}F_{4½}^{{}^{\circ}}$—*e* ^6^F_4½_	
7239.600	3*s*	13809.02	−0.03	*b* ^4^G_3½_—*z* ${\;}^{4}G_{4½}^{{}^{\circ}}$	
7247.821	10*s*	13793.45	0.06	*b* ^4^G_3½_—*z* ${\;}^{4}G_{3½}^{{}^{\circ}}$	
7275.690	1	13740.62	−0.06	*b* ^4^G_2½_—*z* ${\;}^{4}G_{3½}^{{}^{\circ}}$	
7283.80	100	13725.32	−0.05	*y* ${\;}^{6}P_{1½}^{{}^{\circ}}$—*f* ^6^S_2½_	
7287.10	1HH	13719.10	−0.05	*a* ^6^D_3½_—*z* ${\;}^{4}P_{2½}^{{}^{\circ}}$	
7301.38	1HH	13692.27	0.08	*y* ${\;}^{6}F_{5½}^{{}^{\circ}}$—*e* ^6^F_5½_	
7302.89	300	13689.44	−0.06	*y* ${\;}^{6}P_{2½}^{{}^{\circ}}$—*f* ^6^S_2½_	
7322.205	1	13653.34	0.06	*a* ^4^H_5½_—*y* ${\;}^{6}D_{4½}^{{}^{\circ}}$	
7326.500	400	13645.33	−0.05	*y* ${\;}^{6}P_{3½}^{{}^{\circ}}$—*f* ^6^S_2½_	
7376.85	3	13552.19	−0.04	*a* ^2^H_5½_—*z* ${\;}^{4}G_{5½}^{{}^{\circ}}$	
7378.98	1	13548.28	−0.05	*z* ${\;}^{6}D_{1½}^{{}^{\circ}}$—*h* ^6^D_1½_?	
7443.50	1	13430.85	0.23	*a* ^4^H_4½_—*y* ${\;}^{6}D_{3½}^{{}^{\circ}}$	
7446.16	1	13426.05	−0.04	*a* ^2^H_4½_—*z* ${\;}^{4}G_{4½}^{{}^{\circ}}$	
7667.89	1H	13037.81	−0.06	*z* ${\;}^{6}D_{3½}^{{}^{\circ}}$—*e* ^4^H_2½_	
7670.42	2H	13033.51	0.01	*z* ${\;}^{6}F_{4½}^{{}^{\circ}}$—*e* ^4^D_3½_	
7677.46	2H*l*	13021.56	−0.05	*z* ${\;}^{6}F_{1½}^{{}^{\circ}}$—*i* ^6^D_0½_	
7680.22	50H	13016.88	0.02	*z* ${\;}^{4}F_{4½}^{{}^{\circ}}$—*f* ^4^D_3½_	
7706.52	1HHh	12972.46	0.03	*z* ${\;}^{6}F_{2½}^{{}^{\circ}}$—*i* ^6^D_1½_	
7709.98	3HHH	12966.64	$\{\begin{array}{r} \begin{array}{r} 0.19\\ -0.07 \end{array} \end{array}$	*z* ${\;}^{6}F_{2½}^{{}^{\circ}}$—*e* ^4^D_2½_ *z* ${\;}^{6}F_{3½}^{{}^{\circ}}$—*i* ^6^D_2½_	
7712.42	10H	12962.54	0.02	*z* ${\;}^{4}F_{3½}^{{}^{\circ}}$—*f* ^4^D_2½_	
7727.07	1H	12937.96	−0.04	*z* ${\;}^{6}F_{3½}^{{}^{\circ}}$—*e* ^4^D_3½_	
7733.24	10H	12927.64	0.01	*z* ${\;}^{6}F_{4½}^{{}^{\circ}}$—*i* ^6^D_3½_	
7734.43	5H	12925.66	0.05	*z* ${\;}^{4}F_{2½}^{{}^{\circ}}$—*f* ^4^D_1½_	
7735.77	1H	12923.41	−0.07	*z* ${\;}^{6}F_{1½}^{{}^{\circ}}$—*i* ^6^D_1½_	
7737.16	3*s*	12921.09	0.03	*b* ^4^G_5½_—*y* ${\;}^{4}F_{4½}^{{}^{\circ}}$	
7752.67	1HH	12895.24	$\{\begin{array}{r} \begin{array}{r} -0.03\\ -0.05 \end{array} \end{array}$	*z* ${\;}^{6}F_{0½}^{{}^{\circ}}$—*i* ^6^D_1½_ *z* ${\;}^{6}F_{2½}^{{}^{\circ}}$—*i* ^6^D_2½_	
7755.15	3HHH	12891.11	0.01	*z* ${\;}^{4}F_{1½}^{{}^{\circ}}$—*f* ^4^D_0½_	
7764.72	50H	12875.23	0.01	*z* ${\;}^{6}F_{5½}^{{}^{\circ}}$—*i* ^6^D_4½_	
7782.2	1*h*	12846.3	0.0	*z* ${\;}^{6}F_{1½}^{{}^{\circ}}$—*i* ^6^D_2½_	
7790.82	3H	12832.10	−0.03	*z* ${\;}^{6}F_{3½}^{{}^{\circ}}$—*i* ^6^D_3½_	
7806.00	1HHH	12807.14	−0.03	*z* ${\;}^{4}F_{1½}^{{}^{\circ}}$—*f* ^4^D_1½_	
7816.61	3H	12789.76	0.08	*z* ${\;}^{4}F_{2½}^{{}^{\circ}}$—*f* ^4^D_2½_	
7821.25	3H	12782.17	0.00	*z* ${\;}^{4}F_{3½}^{{}^{\circ}}$—*f* ^4^D_3½_	
7834.34	10*h*	12760.81	$\{\begin{array}{r} \begin{array}{r} 0.10\\ -0.06 \end{array} \end{array}$	*z* ${\;}^{6}F_{2½}^{{}^{\circ}}$—*i* ^6^D_3½_ *z* ${\;}^{6}F_{4½}^{{}^{\circ}}$—*i* ^6^D_4½_	
7854.24	3	12728.48	−0.18	*b* ^4^G_4½_—*y* ${\;}^{4}F_{3½}^{{}^{\circ}}$	
7865.36	2	12710.48	−0.20	*b* ^4^G_4½_—*y* ${\;}^{4}F_{4½}^{{}^{\circ}}$	
7889.61	1*h*	12671.42	0.18	*z* ${\;}^{4}F_{1½}^{{}^{\circ}}$—*f* ^4^D_2½_	
7911.68	2	12636.07	0.06	*b* ^4^G_3½_—*y* ${\;}^{4}F_{2½}^{{}^{\circ}}$	
7920.44	2	12022.10	0.04	*b* ^4^G_3½_—*y* ${\;}^{4}F_{3½}^{{}^{\circ}}$	
7928.45	4	12609.34	0.01	*z* ${\;}^{4}F_{2½}^{{}^{\circ}}$—*f* ^4^D_3½_	
7938.52	2	12593.35	0.01	*b* ^4^G_2__½_—*y* ${\;}^{4}F_{1½}^{{}^{\circ}}$	
8043.37	8	12429.18	0.00	*a* ^4^D_3½_—*y* ${\;}^{6}P_{2½}^{{}^{\circ}}$	
8194.06	1	12200.61	−0.15	*z* ${\;}^{4}H_{6½}^{{}^{\circ}}$—*e* ^4^G_5½_	
8210.16	2	12176.69	0.04	*a* ${\;}^{4}D_{2½}^{{}^{\circ}}$—*y* ${\;}^{6}P_{2½}^{{}^{\circ}}$	
8212.43	40*hl*	12173.32	0.00	*z* ${\;}^{4}F_{4½}^{{}^{\circ}}$—*e* ^4^D_3½_	
8234.43	1	12140.80	0.02	*a* ^4^D_2½_—*y* ${\;}^{6}P_{1½}^{{}^{\circ}}$	
8251.64	5*h*	12115.48	0.04	*b* ^4^P_2½_—*z* ${\;}^{4}D_{2½}^{{}^{\circ}}$	
8284.48	4*h*	12067.45	0.00	*z* ${\;}^{4}F_{4½}^{{}^{\circ}}$—*i* ^6^D_3½_	
8304.42	5H*l*	12038.48	−0.02	*z* ${\;}^{4}F_{3½}^{{}^{\circ}}$—*e* ^4^D_2½_	
8353.79	2H	11967.33	−0.01	*z* ${\;}^{4}F_{3½}^{{}^{\circ}}$—*i* ^6^D_2½_	
8373.93	4*h*	11938.54	−0.09	*z* ${\;}^{4}F_{3½}^{{}^{\circ}}$—*e* ^4^D_3½_	
8380.77	40	11928.80	0.02	*b* ^4^P_2½_—*z* ${\;}^{4}D_{3½}^{{}^{\circ}}$	
8395.87	10H	11907.35	−0.09	*e* ^6^S_2½_—*t* ${\;}^{6}P_{1½}^{{}^{\circ}}$	
8409.88	15H	11887.51	−0.14	*e* ^6^S_2½_—*t* ${\;}^{6}P_{2½}^{{}^{\circ}}$	
8421.12	2H	11871.65	0.01	*z* ${\;}^{4}F_{2½}^{{}^{\circ}}$—*i* ^6^D_1½_	
8431.20	20H	11857.45	−0.04	*e* ^6^S_2½_—*t* ${\;}^{6}P_{3½}^{{}^{\circ}}$	
8476.3	1H	11794.4	−0.1	*z* ${\;}^{4}F_{2½}^{{}^{\circ}}$—*i* ^6^D_2½_	
8481.70	3	11786.85	−0.05	*z* ${\;}^{4}F_{1½}^{{}^{\circ}}$—*e* ^4^D_1½_	
8506.0	1H	11753.18	−0.02	*z* ${\;}^{4}F_{1½}^{{}^{\circ}}$—*i* ^6^D_1½_	
8521.57	10*h*	11731.71	0.01	*z* ${\;}^{4}D_{3½}^{{}^{\circ}}$—*f* ^4^D_2½_	
8558.63	8*h*	11680.91	−0.06	*z* ${\;}^{4}D_{2½}^{{}^{\circ}}$—*f* ^4^D_1½_	
8000.34	5H	1102 1.26	−0.07	*z* ${\;}^{6}F_{5½}^{{}^{\circ}}$—*g* ^6^D_4½_	
8602.1	2H	11021.9	0.0	*z* ${\;}^{4}D_{1½}^{{}^{\circ}}$—*f* ^4^D_0½_	
8603.03	5	11620.62	0.10	*b* ^4^P_1½_—*z* ${\;}^{4}D_{1½}^{{}^{\circ}}$	
8654.63	40*h*	11551.34	−0.01	*z* ${\;}^{4}D_{3½}^{{}^{\circ}}$—*f* ^4^D_3½_	
8659.38	10*h*	11545.00	−0.04	*z* ${\;}^{4}D_{2½}^{{}^{\circ}}$—*f* ^4^D_2½_	
8664.6	2*h*	11538.0	0.0	*z* ${\;}^{4}D_{1½}^{{}^{\circ}}$—*f* ^4^D_1½_	
8666.3	2*h*	11535.8	*−*0.1	*z* ${\;}^{4}D_{0½}^{{}^{\circ}}$—*f* ^4^D_0½_	
8670.92	200*c*	11529.74	0.08	*y* ${\;}^{6}P_{1½}^{{}^{\circ}}$—*e* ^6^D_0½_	
8672.06	300*c*	11528.12	−0.05	*y* ${\;}^{6}P_{1½}^{{}^{\circ}}$—*e* ^6^D_1½_	
8673.97	200*c*	11525.58	−0.05	*y* ${\;}^{6}P_{1½}^{{}^{\circ}}$—*e* ^6^D_2½_	
8680.24	2*h*	11517.26	−0.25	*z* ${\;}^{6}F_{4½}^{{}^{\circ}}$—*g* ^6^D_3½_	
8699.13	100*c*	11492.25	−0.05	*y* ${\;}^{6}P_{2½}^{{}^{\circ}}$—*e* ^6^D_1½_	
8701.05	300*c*	11489.71	−0.05	*y* ${\;}^{6}P_{2½}^{{}^{\circ}}$—*e* ^6^D_2½_	
8703.76	500*cw*	11486.14	−0.07	*y* ${\;}^{6}P_{2½}^{{}^{\circ}}$—*e* ^6^D_3½_	
8710.21	10	11477.63	0.07	*b* ^4^P_1½_—*z* ${\;}^{4}D_{2½}^{{}^{\circ}}$	
8717.29	2*h*	11468.31	0.15	*x* ${\;}^{6}P_{3½}^{{}^{\circ}}$—*e* ^4^D_3½_	
8729.80	2*h*	11451.80	−0.17	*z* ${\;}^{4}D_{0½}^{{}^{\circ}}$—*f* ^4^D_1½_	
8734.60	30*c*	11445.58	−0.06	*y* ${\;}^{6}P_{3½}^{{}^{\circ}}$—*e* ^6^D_2½_	
8737.32	300*c*	11442.02	−0.07	*y* ${\;}^{6}P_{3½}^{{}^{\circ}}$—*e* ^6^D_3½_	
8740.93	1000*cw*	11437.29	−0.02	*y* ${\;}^{6}P_{3½}^{{}^{\circ}}$—*e* ^6^D_4½_	
8767.96	5*h*	11402.03	−0.05	*z* ${\;}^{4}D_{1½}^{{}^{\circ}}$—*f* ^4^D_2½_	
8796.83	6*h*	11364.61	−0.08	*z* ${\;}^{4}D_{2½}^{{}^{\circ}}$—*f* ^4^D_3½_	
8798.66	3*h*	11362.25	$\{\begin{array}{r} \begin{array}{r} -0.04\\ 0.10 \end{array} \end{array}$	*x* ${\;}^{6}P_{3½}^{{}^{\circ}}$—*i* ^6^D_3½_ *y* ${\;}^{8}P_{4½}^{{}^{\circ}}$—*e* ^8^P_4__½_	
8820.26	4*h*	11334.43	−0.25	*x* ${\;}^{6}P_{2½}^{{}^{\circ}}$—*i* ^6^D_2½_	
8827.83	3	11324.70	0.03	*b* ^4^P_0½_—*z* ${\;}^{4}D_{0½}^{{}^{\circ}}$	
8842.48	3*h*	11305.94	−0.03	*x* ${\;}^{6}P_{2½}^{{}^{\circ}}$—*e* ^4^D_3½_	
8895.9	4	11238.72	0.09	*b* ^4^P_0½_—*z* ${\;}^{4}D_{1½}^{{}^{\circ}}$	
8901.0	2*p*	11231.0	0.0	*x* ${\;}^{6}P_{1½}^{{}^{\circ}}$—*i* ^6^D_2½_	
8926.06	15*h*	11200.08	−0.02	*x* ${\;}^{6}P_{2½}^{{}^{\circ}}$—*i* ^6^D_3½_	
8929.72	60*h*	11195.49	−0.04	*x* ${\;}^{6}P_{3½}^{{}^{\circ}}$—*i* ^6^D_4½_	
8932.96	2	11191.43	0.03	*y* ${\;}^{8}P_{4½}^{{}^{\circ}}$—*e* ^8^P_3½_	
9084.29	30	11005.00	0.12	*a* ^4^F_1½_—*z* ${\;}^{4}D_{0½}^{{}^{\circ}}$	
9101.2	3*hw*	10984.6	0.0	*e* ^8^D_5½_—*t* ^6^F°	
9114.02	50	10969.10	0.19	*a* ^4^F_2½_—*z* ${\;}^{4}D_{1½}^{{}^{\circ}}$	
9155.85	5	10918.98	0.14	*a* ^4^F_1½_—*z* ${\;}^{4}D_{1½}^{{}^{\circ}}$	
9172.09	100	10899.66	0.10	*a* ^4^F_3½_—*z* ${\;}^{4}D_{2½}^{{}^{\circ}}$	
9234.40	10	10826.11	0.16	*a* ^4^F_2½_—*z* ${\;}^{4}D_{2½}^{{}^{\circ}}$	
9243.29	150	10815.69	0.12	*a* ^4^F_4½_—*z* ${\;}^{4}D_{3½}^{{}^{\circ}}$	
9325.16	5	10720.74	−0.03	*y* ${\;}^{4}P_{2½}^{{}^{\circ}}$—*f* ^4^D_1½_	
9331.90	20*h*	10712.99	0.09	*a* ^4^F_3½_—*z* ${\;}^{4}D_{3½}^{{}^{\circ}}$	
9336.47	40*h*	10707.75	−0.06	*z* ${\;}^{4}D_{3½}^{{}^{\circ}}$—*e* ^4^D_3½_	
9412.78	10*h*	10620.94	−0.08	*z* ${\;}^{4}D_{2½}^{{}^{\circ}}$—*e* ^4^D_2½_	
9429.58	30*h*	10602.02	0.08	*z* ${\;}^{4}D_{3½}^{{}^{\circ}}$—*i* ^6^D_3½_	
9444.90	40	10584.82	−0.02	*y* ${\;}^{4}P_{2½}^{{}^{\circ}}$—*f* ^4^D_2½_	
9474.9	2	10551.3	0.0	*y* ${\;}^{4}P_{1½}^{{}^{\circ}}$—*f* ^4^D_0½_	
9476.57	4*h*	10549.45	−0.41	*z* ${\;}^{4}D_{2½}^{{}^{\circ}}$—*i* ^6^D_2½_?	
9502.12	6*h*	10521.08	−0.07	*z* ${\;}^{4}D_{2½}^{{}^{\circ}}$—*e* ^4^D_3½_	
9335.72	5*h*	10484.01	−0.03	*z* ${\;}^{4}D_{1½}^{{}^{\circ}}$—*i* ^6^D_1½_	
9550.80	20*h*	10467.46	0.07	*y* ${\;}^{4}P_{1½}^{{}^{\circ}}$—*f* ^4^D_1½_	
9584.0	10*h*	10431.2	−0.5	*z* ${\;}^{4}D_{0½}^{{}^{\circ}}$—*e* ^4^D_1½_	
9598.7	3	10415.2	−0.1	*z* ${\;}^{4}D_{2½}^{{}^{\circ}}$—*i* ^6^D_3½_	
9606.71	6*h*	10406.54	0.00	*y* ${\;}^{4}P_{0½}^{{}^{\circ}}$—*f* ^4^D_0½_	
9608.56	100*h*	10404.54	0.05	*y* ${\;}^{4}P_{2½}^{{}^{\circ}}$—*f* ^4^D_3½_	
9676.50	40	10331.49	0.03	*y* ${\;}^{4}P_{1½}^{{}^{\circ}}$—*f* ^4^D_2½_	
9684.9	15	10322.5	−0.1	*y* ${\;}^{4}P_{0½}^{{}^{\circ}}$—*f* ^4^D_1½_	
9845.1	5H	10154.54	0.0	*e* ^8^D_5½_—*u* ^6^F°	
10045.4	4H	9952.08	−0.09	*x* ${\;}^{6}P_{3½}^{{}^{\circ}}$—*g* ^6^D_3½_	
10053.1	10H	9944.46	−0.18	*x* ${\;}^{6}P_{3½}^{{}^{\circ}}$—*g* ^6^D_4½_	
10164.9	2*h*	9835.08	−0.15	*b* ^2^I_5½_—*z* ^8^F°?	
10204.9	1H	9796.53	−0.38	*x* ${\;}^{6}P_{2½}^{{}^{\circ}}$—*g* ^6^D_1½_	
10208.2	2H	9793.36	−1.19	*x* ${\;}^{6}P_{2½}^{{}^{\circ}}$—*g* ^6^D_2½_?	
10212.3	5H	9789.43	−0.55	*x* ${\;}^{6}P_{2½}^{{}^{\circ}}$—*g* ^6^D_3½_	
10300.7	5*h*	9705.42	−0.11	*z* ${\;}^{6}D_{3½}^{{}^{\circ}}$—*e* ^4^P_2½_	
10313.35	2*h*	9693.51	−0.34	*x* ${\;}^{6}P_{1½}^{{}^{\circ}}$—*g* ^6^D_1½_	
10316.05	5*h*	9690.97	0.24	*x* ${\;}^{6}P_{1½}^{{}^{\circ}}$—*g* ^6^D_0½_	
10349.3	2H	9659.84	0.0	*e* ^6^D_4½_—*u* ^6^F°	
10456.60	3	9560.72	−0.23	*y* ${\;}^{4}P_{2½}^{{}^{\circ}}$—*e* ^4^D_3½_	
10561.2	6*h*	9465.94	−0.44	*x* ${\;}^{6}P_{3½}^{{}^{\circ}}$—*h* ^6^S_2½_?	
10621.9	6	9411.93	−0.23	*z* ${\;}^{6}F_{5½}^{{}^{\circ}}$—*f* ^6^D_4½_	
10643.0	10H	9393.26	−0.06	*y* ${\;}^{8}P_{2½}^{{}^{\circ}}$—*g* ^8^D_3½_	
10664.1	15H	9374.69	−0.24	*y* ${\;}^{8}P_{3½}^{{}^{\circ}}$—*g* ^8^D_4½_	
10686.8	2	9354.78	−0.17	*y* ${\;}^{4}D_{3½}^{{}^{\circ}}$—*e* ^4^G_4½_	
10692.2	20H	9350.05	0.10	*y* ${\;}^{8}P_{4½}^{{}^{\circ}}$—*g* ^8^D_5½_	
10745.32	6*h*	9303.83	−0.36	*x* ${\;}^{6}P_{2½}^{{}^{\circ}}$—*h* ^6^S_2½_	
10856.08	2*h*	9208.91	−0.23	*z* ${\;}^{6}F_{3½}^{{}^{\circ}}$—*f* ^6^D_2½_	
10865.85	2*h*	9200.63	−0.50	*x* ${\;}^{6}P_{1½}^{{}^{\circ}}$—*h* ^6^S_2½_	
10938.65	3*h*	9139.40	−0.11	*z* ${\;}^{6}F_{2½}^{{}^{\circ}}$—*f* ^6^D_1½_	
11163.3	2	8955.47	−0.04	*x* ${\;}^{6}D_{4½}^{{}^{\circ}}$—*e* ^6^F_5½_	
11378.8	100*w*	8785.89	0.0	*e* ^8^D_5½_—*y* ${\;}^{8}F_{6½}^{{}^{\circ}}$	
11497.61	40	8695.08	−0.15	*e* ^6^S_2½_—*v* ${\;}^{6}P_{1½}^{{}^{\circ}}$	
11613.24	50	8608.50	−0.10	*e* ^6^S_2½_—*v* ${\;}^{6}P_{2½}^{{}^{\circ}}$	
11644.84	3	8585.14	−0.24	*a* ^6^F_4½_—*z* ${\;}^{6}F_{3½}^{{}^{\circ}}$	
11783.58	60	8484.07	−0.08	*e* ^6^S_2½_—*v* ${\;}^{6}P_{3½}^{{}^{\circ}}$	
12899.7	80	7750.0	0.0	*a* ^6^D_4½_—*z* ${\;}^{6}P_{3½}^{{}^{\circ}}$	
12975.9	40	7704.5	0.0	*a* ^4^D_3½_—*z* ${\;}^{4}P_{2½}^{{}^{\circ}}$	
13294.1	50	7520.1	−0.1	*a* ^6^D_3½_—*z* ${\;}^{6}P_{3½}^{{}^{\circ}}$	
13317.9	30	7506.7	0.7	*a* ^6^D_3½_—*z* ${\;}^{6}P_{2½}^{{}^{\circ}}$	
13415.9	80	7451.8	−0.2	*a* ^4^D_2½_—*z* ${\;}^{4}P_{2½}^{{}^{\circ}}$	
13500.1	100	7405.4	0.0	*a* ^4^D_1½_—*z* ${\;}^{4}P_{0½}^{{}^{\circ}}$	
13625.7	200	7337.1	0.6	*a* ^6^D_2½_—*z* ${\;}^{6}P_{2½}^{{}^{\circ}}$	
13684.6	80	7305.5	−0.6	*a* ^4^D_0½_—*z* ${\;}^{4}P_{0½}^{{}^{\circ}}$	
13863.8	100	7211.1	0.3	*a* ^6^D_1½_—*z* ${\;}^{6}P_{1½}^{{}^{\circ}}$	
13997.0	120	7142.4	0.2	*a* ^6^D_0½_—*z* ${\;}^{6}P_{1½}^{{}^{\circ}}$	
14969.9	30	6678.2	−0.3	*y* ${\;}^{8}P_{4½}^{{}^{\circ}}$—*f* ^8^D_5½_	
15217.9	80	6569.4	−0.1	*e* ^8^S_3__½_—*y* ${\;}^{8}P_{3½}^{{}^{\circ}}$	
15263.1	200	6550.0	−0.1	*e* ^8^S_3__½_—*y* ${\;}^{8}P_{2½}^{{}^{\circ}}$	
15964.9	200	6262.1	0.2	*e* ^8^D_5½_—*z* ^8^F°	
17335.2	80	5767.0	−0.2	*e* ^6^D_4½_—*z* ^8^F°	
17607.5	20	5677.9	−0.2	*e* ^6^S_2__½_—*y* ${\;}^{6}P_{2½}^{{}^{\circ}}$	

**Table 2:** Even terms of *Mn I*

Config.	Desig.	*J*	Level	Interval	Obs. g
					
3*d*^5^ 4*s*^2^	*a* ^6^S	2½	0.00		1.999
3*d*^6^(*a* ^5^D)4*s*	*a* ^6^D	4½	17052.29	−229.71 −169.52 −116.96 −68.67	1.559
		3½	17282.00		1.584
		2½	17451.52		1.657
		1½	17568.48		1.866
		0½	17637.15		3.327
3*d*^6^(*a* ^5^D)4*s*	*a* ^4^D	3½	23296.67	−252.53 −170.32 −99.35	1.427
		2½	23549.20		1.368
		1½	23719.52		1.198
		0½	23818.87		0.000
3*d*^5^ 4*s*^2^	*a* ^4^G	5½	25265.74	−19.69 −2.31 6.70	1.270
		4½	25285.43		1.173
		3½	25287.74		
		2½	25281.04		
3*d*^5^ 4*s*^2^	*a* ^4^P	2½	27201.54	−46.46 −33.85	1.597
		1½	27248.00		1.730
		0½	27281.85		2.666
3*d*^5^ 4*s*^2^	*b* ^4^D	3½	30354.21	−65.40 −6.10 13.97	1.425
		2½	30419.61		1.38
		1½	30425.71		
		0½	30411.74		0.111
3*d*^6^(*a* ^3^P)4*s*	*b* ^4^P	2½	33825.49	−637.88 −381.89	1.602
		1½	34463.37		1.730
		0½	34845.26		2.655
3*d*^6^(*a*^3^H)4*s*	*a* ^4^H	6½	34138.88	−111.64 −93.38 −79.37	1.231
		5½	34250.52		1.135
		4½	34343.90		0.971
		3½	34423.27		0.665
3*d*^6^(*a* ^3^F) 4*s*	*a* ^4^F	4½	34938.70	−102.67 −73.61 −50.07	1.328
		3½	35041.37		1.238
		2½	35114.98		1.024
		1½	35165.05		0.430
3*d*^5^ 4*s*^2^	*a* ^2^I	5½	37148.66	15.59	0.94
		6½	37164.25		
3*d*^6^(a ^3^G)4*s*	*b* ^4^G	5½	37420.24	−210.58 −106.40 −52.71	1.263
		4½	37630.62		1.163
		3½	37737.22		0.989
		2½	37789.93		0.59
3*d*^6^(^3^P)4*s*	*a* ^2^P	1½	37586.03	−765.75	
		0½	38351.78		0.675
3*d*^6^(*a* ^3^H)4*s*	*a* ^2^H	5½	38008.70	−111.48	1.098
		4½	38120.18		0.914
3*d*^6^(*a* ^3^F) 4*s*	*a* ^2^F	3½	38669.60	−265.34	1.128
		2½	38934.94		
3*d*^5^ 4*s*(*a* ^7^S)5*s*	*e* ^8^S	3½	39431.31		2.000
3*d*^6^(^3^G) 4*s*	*a* ^2^G	4½	41031.48	−198.86	1.118
		3½	41230.30		0.88
3*d*^5^ 4*s* (*a* ^7^S)5*s*	*e* ^6^S	2½	41403.93		1.997
	*b* ^2^I	6½	43053.30	−85.97	1.07
		5½	43139.27		0.924
3*d*^5^ 4*s* (*a* ^7^S)4*d*	*e* ^8^D	1½	46706.09	0.94 1.30 1.82 2.43	
		2½	46707.03		
		3½	46708.33		
		4½	46710.15		
		5½	46712.58		
3*d*^5^ 4*s*(*a* ^7^S)4*d*	*e* ^6^D	4½	47207.28	−4.78 −3.55 −2.54 −1.49	1.554
		3½	47212.06		1.581
		2½	47215.61		1.634
		1½	47218.15		1.759
		0½	47219.64		3.934
3*d*^5^ 4*s* (*a* ^5^S) 5*s*	*f* ^6^S	2½	49415.35		2.00
3*d*^5^4*s*(*a* ^5^S)5*s*	*e* ^4^S	1½	49591.51		1.998
3*d*^5^4*s*(*a* ^7^S)6*s*	*f* ^8^S	3½	50157.63		1.995
3*d*^5^4*s*(*a* ^7^S)6*s*	*g* ^6^S	2½	50904.68		
3*d*^7^	*e* ^4^P	2½	51638.17	−80.05 −69.70	1.601
		1½	51718.22		1.733
		0½	51787.92		2.65
3*d*^5^4*s*(*a* ^7^S)5*d*	*f* ^8^D	1½	} 52702.48?	0.6 2.1	
		2½			
		3½			
		4½	52703.1		
		5½	52705.23		
3*d*^5^4*s*(*a* ^7^S)5*d*	*f* ^6^D	4½	52726.39	−4.02 −2.81 −1.79 −0.82	
		3½	52730.41		
		2½	52733.22		
		1½	52735.01		
		0½	52735.83		
3*d*^5^4*s*(*a* ^7^S)7*s*	*h* ^6^S	2½	54460.30		
3*d*^5^4*s*(*a* ^5^S)4*d*	*g* ^6^D	4½	54938.56	−7.53 −4.57 −2.36 3.12	
		3½	54946.09		
		2½	54950.66		
		1½	54953.02		
		0½	54949.90		
3*d*^5^4*s*(*a* ^7^S)6*d*	*g* ^8^D	1½		0.94 1.00	
		2½			
		3½	55374.76		
		4½	55375.70		
		5½	55376.70		
3*d*^5^4*s*(*a* ^7^S)6*d*	*h* ^6^D	4½	55681.90	−6.20 −2.70 −1.10 −0.50	
		3½	55688.10		
		2½	55690.80		
		1½	55691.90		
		0½	55692.40		
3*d*^5^4*s*(*a* ^7^S)8*s*	*g* ^8^S	3½	56144.16		
3*d*^6^(*a* ^5^D) 5*s*	*i* ^6^D	4½	56189.45	−166.76 −134.58 −77.14 −98.13	
		3½	56356.21		1.57
		2½	56490.79		
		1½	56567.93		
		0½	56666.06		
	*e* ^4^D	3½	56462.08	−99.87 −39.68	
		2½	56561.95		
		1½	56601.63		
		0½			
3*d*^5^4*s*(*a* ^7^S)7*d*	*h* ^8^D	1½ to 5½	} 56801.4?		
3*d*^5^4*p*^2^	*e* ^8^P	2½	57086.33	131.82 170.75	2.27
		3½	57218.15		
		4½	57388.90		1.767
3*d*^6^(*a* ^5^D)5*s*	*f* ^4^D	3½	57305.62	−180.35 −135.93 −83.93	
		2½	57485.97		1.372
		1½	57621.90		
		0½	57705.83		
Mn II (^7^S_3_)	***Limit***		**59970**		
3*d*^6^(*a* ^5^D)5*d*	*e* ^6^F	5½	61713.62	−316.56 −264.48 −611.15 −177.43	
		4½	62030.18		
		3½	62294.66		
		2½	62905.81		
		1½	63083.24		
		0½			
3*d*^6^(*a* ^5^D)4*d*	*e* ^6^G	6½	62001.09	−133.36 −166.18 −125.85 −88.11 −58.52	
		5½	62134.45		
		4½	62300.63		
		3½	62426.48		
		2½	62514.59		
		1½	62573.11		
3*d*^6^(*a* ^5^D) 4*d*	*e* ^4^G	5½	62295.36	−183.68 −153.73 −120.60	
		4½	62479.04		
		3½	62632.77		
		2½	62753.37		
3*d*^6^(*a* ^5^D)4*d*	*e* ^4^F	4½	63231.43	−192.57	
		3½	63424.00		
		2½			
		1½			
3*d*^5^4*s*(*a* ^5^G) 5*s*	*f* ^4^G	5½	68693.02	−23.20	
		4½	68716.22		1.17
		3½			
		2½			
3*d*^5^4*s*(*a* ^7^S)4*p*	*z* ^8^P°	2½	18402.46	129.18 173.73	2.284
		3½	18531.64		1.938
		4½	18705.37		1.779
3*d*^5^ 4*s* (*a* ^7^S)4*p*	*z* ^6^P°	1½	24779.32	8.73 14.20	2.364
		2½	24788.05		1.875
		3½	24802.25		1.714
3*d*^5^ 4*s*(*a* ^5^S)4*p*	*z*^4^P°	2½	31001.15	−75.27 −48.53	1.60
		1½	31076.42		1.732
		0½	31124.95		2.668
3*d*^5^ 4*s* (*a* ^5^S)4*p*	*y* ^6^P°	1½	35689.98	35.87 44.12	2.400
		2½	35725.85		1.886
		3½	35769.97		1.712
3*d*^6^(*a* ^5^D)4*p*	*z* ^6^D°	4½	41789.48	−143.16 −121.09 −89.84 −54.99	1.556
		3½	41932.64		1.587
		2½	42053.73		1.653
		1½	42143.57		1.867
		0½	42198.56		3.317
3*d*^6^(*a* ^5^D)4*p*	*z* ^6^F°	5½	43314.23	−114.35 −95.50 −71.42 −48.95 −28.21	1.464
		4½	43428.58		1.431
		3½	43524.08		1.395
		2½	43595.50		1.310
		1½	43644.45		1.068
		0½	43672.66		−0.602
3*d*^6^(*a* ^5^D) 4*p*	*z* ^4^F°	4½	44288.76	−234.69 −172.84 −118.44	1.317
		3½	44523.45		1.240
		2½	44696.29		1.030
		1½	44814.73		0.400
3*d*^6^(*a* ^5^D)4*p*	*x* ^6^P°	3½	44993.92	−162.19 −103.06	1.717
		2½	45156.11		1.885
		1½	45259.17		2.399
3*d*^6^(*a* ^5^D)4*p*	*z* ^4^D°	3½	45754.27	−186.66 −142.96 −86.04	1.427
		2½	45940.93		1.372
		1½	46083.89		1.200
		0½	46169.93		0.000
3*d*^5^ 4*s*(*a* ^7^S)5*p*	*y* ^8^P°	2½	45981.44	19.33 25.98	
		3½	46000.77		
		4½	46026.75		
3*d*^6^(*a* ^5^D)4*p*	*y* ^4^P°	2½	46901.13	−253.38 −144.78	1.595
		1½	47154.51		1.732
		0½	47299.29		2.666
3*d*^5^ 4*s* (*a* ^7^S)5*p*	*w* ^6^P°	3½	47387.62	−271.90 −122.91	1.713
		2½	47659.52		1.952
		1½	47782.43		2.666
3*d*^5^ 4*s*(*a* ^5^P)4*p*	*y* ^6^D°	0½	47452.16	14.50 287.33 20.53 129.28	3.174
		1½	47466.66		
		2½	47753.99		1.820
		3½	47774.52		1.594
		4½	47903.80		1.540
3*d*^5^ 4*s* (*a* ^5^G)4*p*	*y* ^6^F°	5½	48021.43	−146.58 −57.98 −44.92 −30.07 −17.14	1.460
		4½	48168.01		1.432
		3½	48225.99		1.043
		2½	48270.91		1.319
		1½	48300.98		1.068
		0½	48318.12		−0.496
3*d*^5^ 4*s*(*a* ^5^P)4*p*	*v* ^6^P°	3½	49888.08	−124.45 −86.63	1.711
		2½	50012.53		1.888
		1½	50099.16		2.398
3*d*^5^ 4*s* (*a* ^5^G)4*p*	*z* ^4^H°	3½	50065.46	7.13 8.72 13.29	
		4½	50072.59		
		5½	50081.31		
		6½	50094.60		1.22
3*d*^5^ 4*s*(*a* ^5^G)4*p*	*y* ^4^F°	4½	50341.30	−17.98 −13.95 −10.04	1.318
		3½	50359.28		1.242
		2½	50373.23		1.03
		1½	50383.27		
3*d*^5^ 4*s* (*b* ^5^D)4*p*	*x* ^6^F°	0½	50818.64	44.41 68.37 83.53 85.54 68.69	−0.62
		1½	50863.05		1.07
		2½	50931.42		1.316
		3½	51014.95		
		4½	51100.49		
		5½	51169.18		
3*d*^5^ 4*s* (*a* ^5^P)4*p*	*x* ^4^P°	2½	51305.41	−140.24 −107.13	1.591
		1½	51445.65		1.728
		0½	51552.78		2.664
3*d*^5^ 4*s*(*a* ^5^G)4*p*	*z* ^4^G°	2½	51515.63	14.98 15.66 14.66	
		3½	51530.61		
		4½	51546.27		
		5½	51560.93		1.273
3*d*^5^ 4*s*(*b* ^5^D)4*p*	*u* ^6^P°	1½	52015.00	113.65 124.59	
		2½	52128.65		
		3½	52253.24		1.71
3*d*^5^ 4*s*(*b* ^5^D)4*p*	*x* ^6^D°	4½	52758.11	−111.99 −13.77 0.00 0.77	1.552
		3½	52870.10		1.57
		2½	52883.87		
		1½	52883.87		
		0½	52883.10		
3*d*^5^ 4*s* (*a* ^7^S)4*f*	*z* ^8^F°	0½ to 6½	}52974.5?		
3*d*^5^ 4*s* (*a* ^7^S)4*f*	*w* ^6^F°	0½		0.10 −0.21 −0.07 0.14	
		1½	52977.93		
		2½	52978.03		
		3½	52977.82		
		4½	52977.75		
		5½	52977.89		
3*d*^5^ 4*s* (*a* ^5^P)4*p*	*y* ^4^D°	0½	53101.32	1.87 6.02 14.88	
		1½	53103.19		
		2½	53109.21		
		3½	53124.09		1.423
3*d*^5^ 4*s*(*a* ^7^S)6*p*	*t* ^6^P°	3½	53261.42	−30.16 −19.79	
		2½	53291.58		
		1½	53311.37		
3*d*^5^ 4*s* (*a* ^5^P)4*p*	*z* ^4^S°	1½	54218.71		1.770
3*d*^5^ 4*s* (*b* ^5^D)4*p*	*x* ^4^D°	3½	55107.52	−78.65 −93.74	1.407
		2½	55186.17		1.365
		1½	55279.91		0.826
		0½			
3*d*^5^ 4*s* (*b* ^5^D)4*p*	*w* ^4^P°	2½	55405.14	36.48 −88.54	
		1½	55368.66		
		0½	55457.20		2.28
3*d*^5^ 4*s* (*a* ^7^S)5*f*	*v* ^6^F°	0½		−0.38 0.15 0.44 0.22	
		1½	55491.95		
		2½	55491.57		
		3½	55492.08		
		4½	55492.52		
		5½	55492.74		
3*d*^5^ 4*s* (*a* ^7^S)5*f*	*y* ^8^F°	6½	55498.5	−0.6 0.0 −0.4 −0.4 0.15	
		5½	$\{\;\begin{array}{c} 55499.09\\ 55499.09 \end{array}$		
		4½			
		3½	55499.5		
		2½	55499.90		
		1½	55499.75		
		0½			
		2½	55923.81		
3*d*^5^ 4*s* (*a* ^5^S)5*p*	*s* ^6^P°?	1½	55996.62	11.29 4.51	
		2½	56007.91		
		3½	56012.42		
3*d*^5^ 4*s* (*a* ^7^S)6*f*	*u* ^6^F°	0½ to 6½	}56867.1		
3*d*^5^ 4*s*(*b* ^3^P)4*p*	*v* ^4^P°	0½	57228.30	132.48 126.30	2.671
		1½	57360.78		1.736
		2½	57487.08		1.590
3*d*^5^ 4*s*(*b* ^3^P)4*p*	*y* ^4^S°	1½	57512.16		2.000
3*d*^5^ 4*s* (*a* ^7^S)7*f*	*t* ^6^F°	0½ to 6½	}57697.2		
3*d*^6^(*a* ^3^H)4*p*	*y* ^4^G°	5½	58075.06	−35.18 −26.45 −23.04	1.269
		4½	58110.24		1.168
		3½	58136.69		0.980
		2½	58159.73		0.578
3*d*^6^(*a* ^3^H)4*p*	*y* ^4^H°	6½	58338.67	−88.63 −58.22 −34.38	1.228
		5½	58427.30		1.133
		4½	58485.52		0.968
		3½	58519.90		0.665
3*d*^6^(*a* ^3^H)4*p*	*z* ^4^I°	7½	58852.60	9.21 −8.10 −15.17	
		6½	58843.39		1.09
		5½	58851.49		
		4½	58866.66		0.73
3*d*^6^(*a* ^3^P)4*p*	*u* ^4^P°	2½	59116.60	−267.85 −183.84	1.558
		1½	59384.45		1.608
		0½	59568.29		1.94
3*d*^6^(*a* ^3^F)4*p*	*x* ^4^F°	4½	59257.44	−32.79 −70.49 −55.43	1.327
		3½	59290.23		1.325
		2½	59360.72		1.11
		1½	59416.15		0.39
3*d*^6^(*a* ^3^P)4*p*	*w* ^4^D°	3½	59339.55	−260.86 −389.42 −152.21	1.362
		2½	59600.35		1.277
		1½	59989.77		1.194
		0½	60141.98		0.17
3*d*^6^(*a* ^3^F)4*p*	*v* ^4^D°	3½	59470.14	−10.66 −47.09 0.53	1.386
		2½	59480.80		1.281
		1½	59527.89		
		0½	59527.36		
3*d*^6^(*a* ^3^H)4*p*	*z* ^2^I°	6½	59617.12	−210.76	1.074
		5½	59827.88		0.93
3*d*^6^(*a* ^3^F) 4*p*	*x* ^4^G°	5½	59652.90	−79.04 −52.37 −33.39	1.239
		4½	59731.94		1.169
		3½	59784.31		0.990
		2½	59817.70		0.584
Mn II (^7^S_3_)	***Limit***	………	**59970**		
3*d*^6^(^3^P)4*p*	*z* ^2^D°	2½	60101.65	−293.99	1.31
		1½	60395.64		0.91
3*d*^6^(*a* ^3^H)4*p*	*z* ^2^G°	4½	60668.49	−70.93	1.112
		3½	60739.42		
	*w* ^4^F°	1½	60760.87	59.48 82.45 36.17	
		2½	60820.35		
		3½	60902.80		
		4½	60938.97		
3*d*^5^ 4*s*(^3^I)4*p*	*x* ^4^H°	6½	60891.48	−42.25 −22.15 −1.33	1.228
		5½	60933.73		1.134
		4½	60955.88		
		3½	60957.21		
3*d*^5^ 4*s* (^3^I)4*p*	*y*^4^I°	7½	61204.54	−21.01 −0.22 14.34	1.20
		6½	61225.55		
		5½	61225.77		
		4½	61211.43		0.75
	*w* ^4^G°	5½	61469.21	−16.13 4.74 9.37	1.164
		4½	61485.34		1.020
		3½	61480.60		1.13
		2½	61471.23		
	*z* ^2^F°	3½	61710.98	−16.48	
		2½	61727.46		
	*y* ^2^G°	4½	61714.52	−71.42	
		3½	61785.94		0.93
		5½	61744.04		
	*y* ^2^I°	5½	61819.07	93.50	
		6½	61912.57		
	*y* ^2^F°	3½	62034.04	−40.98	
		2½	62075.02		0.58
3*d*^6^(^3^P) 4*p*	*z* ^2^P°	1½	62354.76	−36.29	1.24
		0½	62391.05		0.81
3*d*^6^(*a*^3^G)4*p*	*v* ^4^F°	1½	62390.20	97.16 17.93 −112.47	
		2½	62487.36		
		3½	62505.29		
		4½	62392.82		1.35
3*d*^5^ 4*s*(*a* ^5^F)4*p*	*w* ^6^D°	4½	62670.81	−180.66 90.14 −26.30 19.47	
		3½	62851.47		
		2½	62761.33		
		1½	62787.63		
		0½	62768.16		
	*y* ^2^D°	2½	63081.28	−32.83	1.24
		1½	63114.11		0.758
	*x* ^2^F°	2½	63139.70	149.08	
		3½	63288.78		
3*d*^6^(^3^H?)4*p*	*z* ^2^H°	5½	63288.78	−259.71	1.127
		4½	63548.49		0.92
		3½	63319.85?		
	*y* ^2^H°	4½	63347.91	101.22	
		5½	63449.13		
3*d*^6^(^3^G)4*p*	*w* ^4^H°	6½	63363.54	−94.31 13.24 49.16	1.231
		5½	63457.85		1.14
		4½	63444.61		
		3½	63395.45		0.70
		2½	63371.56		
		4½	63374.53		
		2½	63523.82		
		3½	63546.30		
		1½,2½	63583.84		
	*x* ^2^D°	2½	63764.90	−80.42	
		1½	63845.32		
	*x* ^2^H°	5½	64051.91	−3.46	
		4½	64055.37		
	*u* ^4^D°	3½	64409.69	−303.25 28.99 45.27	1.42
		2½	64712.94		
		1½	64683.95		1.22
		0½	64638.68		0.22
	*x* ^2^G°	4½	64585.44	−63.76	1.307
		3½	64649.20		
	*v* ^4^H°	6½	64731.88	−87.65 −68.47 −32.33	1.236
		5½	64819.53		1.137
		4½	64888.00		0.974
		3½	64920.33		
	*w* ^2^F°	2½	64823.21	165.01	
		1½	64988.22		
	*w* ^2^G°	4½	65262.28	−42.85	1.13
		3½	65305.13		
	*v* ^2^F°	3½	65617.37	−31.76	1.015
		2½	65649.13?		
		4½	65768.81		1.12
	*v* ^4^G°	2½	65873.40	2.94 32.58 −21.61	
		3½	65876.34		
		4½	65908.92		1.160
		5½	65887.31		1.259
	*w* ^2^D°	2½	65946.87	−15.03	1.30
		1½	65961.90		
	*u* ^2^F°	2½	66020.63	128.47	
		3½	66149.10		1.14
	*u* ^4^H°	3½	66334.47	21.93 62.15 150.03	0.764
		4½	66356.40		1.022
		5½	66418.55		
		6½	66568.58		1.23
	*u* ^4^G°	2½	66395.19	59.08 68.35 50.98	0.611
		3½	66454.27		0.932
		4½	66522.62		1.13
		5½	66573.60		1.24
		1½	66504.21		
		3½	66600.17		
	*v* ^2^G°	4½	66630.92	−106.90	1.13
		3½	66737.82		0.46
		2½	66654.65?		
	*u* ^4^F°	1½	66843.79	−6.15 −54.59 71.95	0.46
		2½	66837.64		
		3½	66783.05		1.21
		4½	66855.00		1.33
		2½?	66910.02		
		3½	66981.30		1.33
		2½	67008.54		
	*w* ^2^H°	5½	67504.90	−71.94	1.09
		4½	67576.84		0.90
	*t* ^4^G°	5½	67752.84	−66.33 −72.19 −73.51	1.266
		4½	67819.17		
		3½	67891.36		
		2½	67964.87		
	*u* ^2^G°	4½	68286.44	−52.15	1.320
		3½	68338.59		
3*d*^6^(^1^I)4*p*	*z* ^2^K°	7½	68797.56?	−44.96	1.07
		6½	68842.52?		0.93
3*d*^6^(^1^I)4*p*	*x* ^2^I°	6½	69560.88?	−68.97	1.07
		5½	69629.85?		0.924
3*d*^6^(^1^I)4*p*?	*v* ^2^H°	4½	69663.20?	59.76	
		5½	69722.96		1.10

**Table 3:** Energy levels and classified lines of *Mn I*

Year	Reference	Number of levels	Number of lines
			
1922	4	70	169
1926	9	91	257
1949	15	211	711
1952	21	356	1500
1962	This work	404	2030

## Footnotes

4 Figures in brackets indicate the literature references at the end of this paper.
